# Current Trends in Metallic Materials for Body Panels and Structural Members Used in the Automotive Industry

**DOI:** 10.3390/ma17030590

**Published:** 2024-01-25

**Authors:** Tomasz Trzepieciński, Sherwan Mohammed Najm

**Affiliations:** 1 Department of Manufacturing Processes and Production Engineering, Rzeszow University of Technology, Al. Powstańców Warszawy 8, 35-959 Rzeszow, Poland; 2 Kirkuk Technical Institute, Northern Technical University, Kirkuk 36001, Iraq; sherwan.mohammed@gpk.bme.hu; 3 Department of Manufacturing Science and Engineering, Budapest University of Technology and Economics, Műegyetemrkp 3, 1111 Budapest, Hungary

**Keywords:** aluminium alloy, automotive industry, body panel, steel, titanium alloy

## Abstract

The development of lightweight and durable materials for car body panels and load-bearing elements in the automotive industry results from the constant desire to reduce fuel consumption without reducing vehicle performance. The investigations mainly concern the use of these alloys in the automotive industry, which is characterised by mass production series. Increasing the share of lightweight metals in the entire structure is part of the effort to reduce fuel consumption and carbon dioxide emissions into the atmosphere. Taking into account environmental sustainability aspects, metal sheets are easier to recycle than composite materials. At the same time, the last decade has seen an increase in work related to the plastic forming of sheets made of non-ferrous metal alloys. This article provides an up-to-date systematic overview of the basic applications of metallic materials in the automotive industry. The article focuses on the four largest groups of metallic materials: steels, aluminium alloys, titanium alloys, and magnesium alloys. The work draws attention to the limitations in the development of individual material groups and potential development trends of materials used for car body panels and other structural components.

## 1. Introduction

Car manufacturers are guided by technological, material, and economic criteria when selecting materials for car body panels. Meeting all these conditions at the same time is often not possible, which is why many types of materials made of non-ferrous alloys and steel are currently used. Chronologically, steel was the first common construction material used in the automotive industry. With the development of sheet metal production and processing methods, wooden elements in cars were replaced with sections and sheets made of low-carbon (LC) steel with relatively low mechanical strength and low corrosion resistance. The desire to reduce the weight of vehicles and thus reduce fuel consumption and emissions of harmful substances into the atmosphere have resulted in an evolution in the materials of body panels and vehicle chassis components. Carbon steel sheets and sections are beginning to be replaced by their lightweight equivalents, such as aluminium alloys, titanium alloys, and magnesium alloys.

The first uses of steel sheets in the automotive industry were observed in the early 20th century when Henry Ford used them in the mass production of the Model T. In Europe, steel has been used in car production since the early 20th century when German engineer Carl Benz created the first car powered by an internal combustion engine in 1919. Carbon steel is a construction material ideal for the developing automotive industry and is characterised by adequate strength and durability at relatively low production costs, which is one of the main criteria for mass production. Even though carbon steel is not resistant to corrosion, it is one of the most popular materials used in the metal industry, which makes it relatively cheap compared to other materials. Over the years, increasingly better anti-corrosion protection coatings have been developed. Moreover, carbon steel shows very good plastic properties in cold sheet metal forming compared to aluminium or titanium alloys. In the 1970s, the production of steel with increased strength began, which resulted in a reduction in the weight of vehicles. This trend has continued to this day and has been intensified in recent years by climate problems and the need to reduce emissions of harmful substances into the natural environment. The global steel industry responded to the challenge of reducing vehicle weight by launching the Ultra-Light Steel Auto Body (ULSAB) project to reduce body weight by 25% [1,2]. This project initiated the extensive development of innovative Advanced High-Strength Steel (AHSS) grades with sufficient formability.

Conventional deep-drawing quality low-carbon steel sheets have a yield strength of less than 300 MPa and are used to build motorcar bodies. The carbon content in these steels does not exceed 0.12 wt.%. Among Low-Strength Steels (LLs), we can distinguish between Mild Steels (MSs) and Interstitial Free (IF) steels. High-Strength (HS) steels include Isotropic Steels (ISs), Interstitial Free–High-Strength (IF–HS) steels, Bake Hardenable (BH) steels, Carbon–Manganese (CMn) steels, Press Quenched Steels (PQSs), and High-Strength Low-Alloy (HSLA) steels. Dual Phase (DP) steels, Ferrite–Bainite (FB) steels, Complex Phase (CP) steels, Transformation Induced Plasticity (TRIP) steels, and martensitic (MS) steels are classified as Ultra-High-Strength (UHS) steels [3]. The third-generation AHSS (Dual Phase–High Ductility (DH) steels, Complex Phase–High Ductility (CH) steels, TRIP-Aided Bainitic Ferrite (TBF) steels, Carbide-Free Bainite (CFB) steels and Quenching & Partitioning (QP) steels) expand on the previously established first-generation AHSS (DP, CP, TRIP, MS, and Press Hardened Steels—PHSs) and the second-generation AHSS (TWIP) [4]. Sixty percent of the body structure of the Jeep Grand Cherokee L (2021) is made from AHSS and third-generation steels [5].

The development of HS and UHS steels was related to the need to reduce vehicle weight while maintaining the high stiffness of motorcar bodies [6]. One way to meet this criterion is to use steel sheets of increasingly smaller thickness and higher tensile strength while maintaining good formability [7]. In 2005, deep-drawing quality low-alloy steel sheets accounted for approximately 85% of vehicle weight. Meanwhile, in 2015, the share of steel from this group in the weight of vehicles was 40% [8]. According to the Automotive and Transportation Market Research Report (2023) [9], high-strength low-alloy steels currently constitute up to 60% of modern car body structures. HS steels have a tensile strength of up to 550 MPa. Some advanced third-generation steels have a tensile strength exceeding 1000 MPa while maintaining good formability [10]. Steels of increased quality (cold-rolled and hot-rolled) are commonly used for the production of vehicle frames and suspension elements. The use of HS steel ensures increased durability of vehicles and increased safety of passengers. Stainless steel is very rarely used for passenger car body parts due to its high price. In the case of public transport vehicles (buses, trams) and passenger trains, which are heavily used vehicles, the bodies are made of stainless steel.

Currently, aluminium is very important in the construction of car bodies. A body made of aluminium alloys is much lighter than one made of steel. The density of aluminium and its alloys is about 65% lower than that of steel. Reducing the weight of a mid-range vehicle can be as much as 300–400 kg without compromising its performance or safety level. It is assumed that reducing the vehicle weight by 100 kg allows for fuel savings of approximately 0.6 dm^3^ per 100 km. When the vehicle mass is reduced by 100 kg, the fuel consumption is reduced by about 0.4 dm^3^/100 km, and the CO_2_ emission decreases by 7.5 to 12.5 g/km [11]. Aluminium alloys are corrosion resistant and have high mechanical strength. Aluminium alloys with copper, magnesium, silicon, nickel, and manganese are most often used in body structural elements (doors, engine covers, roof elements, boot lids, etc.). Al–Mg and Al–Mg–Mn alloys are characterised by average mechanical strength, but they are highly resistant to corrosion. Al–Mg–Si series alloys have average mechanical strength but high corrosion resistance and good plasticity. Al–Zn–Mg and Al–Zn–Mg–Cu alloys have strengths similar to those of steel body sheets. The first car to use car body panels made of aluminium was the Bugatti 10. In the post-war years, the aluminium-bodied ‘1953 Porsche 356 1500 [12] was created. Nowadays, aluminium alloy sheets are used in the Audi A8, Land Rover Defender, and BMW 5 series. In 2015, Ford introduced the aluminium-bodied F-150. Aluminium and its alloys are eagerly used in the production of trailers and semi-trailers, delivery trucks, and motor tracks. Aluminium alloys are also a material for producing composite car bodies [13,14]. Basic aluminium-based laminates include glass-reinforced aluminium laminates (GLAREs), aramid-reinforced aluminium laminates (ARALLs), carbon fibre-reinforced aluminium laminates (CARALLs), kenaf fibre-reinforced aluminium alloy laminates (CAKRALLs), and flax fibre-reinforced aluminium alloy laminates (CAFRALLs) [15].

Titanium and titanium alloys are characterised by low density (4.43 g/cm^3^) while maintaining good mechanical properties and corrosion resistance. Titanium alloys are characterised by a high tensile strength of 1275 MPa for the Ti-6Al-6V-2Sn alloy. Currently, they are widely used in car production. The density of titanium alloy is only 60% of the density of steel, and the strength can reach more than 800 MPa. Titanium not only has high specific strength but also good toughness. Components made of titanium alloys used in the production of cars are mainly used in car exhaust systems, suspension springs, body parts, and car body frames [16]. Titanium suspension springs provide an opportunity for reducing the weight by upwards of 70% [17]. Crash elements made from Ti-6Al-4V titanium alloy are conceivable in the body because of titanium energy-absorbing deformation behaviour. The use of Ti and Ti alloys can at least be partially considered for the armouring of security vehicles [18]. A significant reduction in vehicle weight and thus fuel consumption in combustion vehicles or electricity consumption in electric vehicles can be achieved by replacing steel with light magnesium alloys. These alloys, compared to other metallic alloys, have the highest strength-to-weight ratio. Properties of magnesium alloys desired by the automotive industry include high mechanical strength and creep resistance. The main area of application of magnesium alloys is currently the drive systems and vehicle rims. The use of magnesium alloys for the production of body panels and structural members is very limited compared to steel or aluminium alloys. However, the use of Mg-based alloys is constantly growing and is focused on the production of brackets, profiles, and extruded interior door components. Plastic processing of magnesium alloys is a technological challenge because these materials are characterised by low plasticity due to their hexagonal close-packed crystal structure. Magnesium alloy sheets, for example the common AZ31 alloy, exhibit good plastic properties at temperatures above 220 °C. The heating of tools associated with hot forming, as well as limitations in the available high-temperature lubricants and the technological complexity of the forming process significantly limit the use of magnesium alloys for the production of body panels.

This article presents an up-to-date overview of the applications of metallic materials in car body panels and bearing components. The article focuses on the four largest groups of metallic materials: steels, aluminium alloys, titanium alloys, and magnesium alloys. Limitations in the development of individual material groups and potential development trends of materials used for car body panels and bearing components are presented.

## 2. Steels

### 2.1. Background

Currently, commonly used materials for body production are various grades of both deep-drawing steels and high-strength low-alloy steels [6,19]. Replacing low-carbon steels with high-strength steels allows for reducing the thickness of the sheets while maintaining or even increasing the strength properties of the car structure. For several decades, despite the optimisation of the design related to the reduction of pollutants emitted by combustion engines, the weight of ‘compact’ cars has been kept practically constant. The weight reduction resulting from the use of thinner, high-strength sheets is balanced by additional equipment in motor vehicles installed to improve the comfort and, above all, the safety of users [20]. A solution is to use composite materials, but due to difficulties with recycling and the high cost of production, their use is not common. Metallic materials continue to be the basis for the construction of mass-produced vehicles. In the context of the requirements of the automotive industry, modern sheet metals should [21]:

- be characterised by high specific strength, defined as the ratio of the material’s strength to its density,
- show high energy absorption capacity in the event of a collision,
- have properties that minimise technological problems in production (including springback) and ensure high efficiency,
- have good weldability,
- show high corrosion resistance.

Steel is coated with several methods to make it resistant to corrosion. The steel sheet metals for car body outer panels are supplied with four different main coatings: electrolytic galvanising, where the sheet is coated with zinc from a sulphate–acid electrolyte, zinc–magnesium coating, hot-dip galvanising (the most frequently used and galvannealed coatings where zinc coating is converted into a zinc–iron coating by heat treatment [22]. Zn–Be and Zn–Ni coatings are electrodeposited [23]. According to Close et al. [24], electrophoretic coating (E-Coat) is the most widespread solution to protect vehicles from corrosion. Many auto makers in Japan use galvannealed steel sheets. The mainstream product in North America is electrogalvanised sheets. In Europe, auto makers mainly use hot-dip galvanised sheets [25]. In the hot-dip galvanising process, coatings with a thickness of between 45 and 150 μm are obtained. The minimum thickness of zinc coatings in accordance with EN ISO 1461 [26] standard depends on the thickness of the galvanised element t and is 45 μm (t < 1.5 mm, coating weight 325 g/m^2^), 55 μm (1.5 mm ≤ t < 3 mm, coating weight 395 g/m^2^), 70 μm (3 mm ≤ t < 6 mm, coating weight 505 g/m^2^) and 85 μm (t ≥ 6 mm, coating weight 610 g/m^2^). A 15-μm-thick galvanised coating generally contains 0.2 wt.% Al and is deposited by continuous hot-dip galvanising with a bath temperature comprised between 400–495 °C [27]. At the vehicle production stage, the sheet metal is protected against corrosion thanks to the use of anodic and cathodic coatings [28]. The conventional finishing process of auto-body panels includes solvent-based surface primer + base coat + clear coat, which is featured by large volatile organic compound emission [29]. Therefore, waterborne coating systems composed of water-soluble melamine formaldehyde and water borne acrylic or alkyd resin eliminates the use of solvents and reduce the volatile organic compounds.

**Figure 1:**
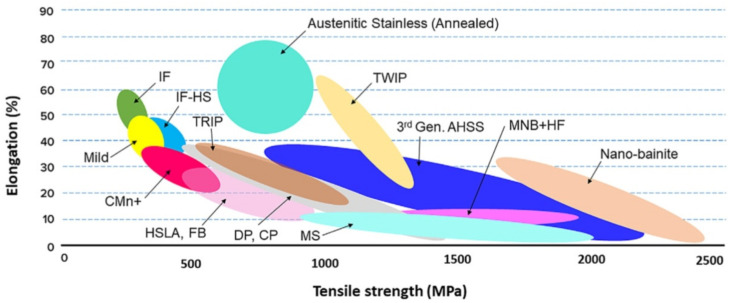
Classification of steels used in the automotive industry(reproduced with permission from Reference [4]; copyright © 2024 Acta Materialia Inc. Published by Elsevier B.V. All rights reserved).

The following material groups of steel are used in the automotive industry (Figure 1) [21,30]:

- soft steels, with the ultimate tensile strength (UTS) R_m_ below 300 MPa and elongation A_80_ above 30% (IF),
- conventional steels, with the UTS from 300 to 700 MPa and elongation A_80_ from 10% to 30% (HS, BH, HSLA),
- advanced steels, with very high UTS above 700 MPa and elongation A_80_ in the range of 5–30% (TRIP, DP, CP, MS).

Chronologically, according to the development of steel sheets for automotive applications, the following groups are distinguished [31]:

- low-carbon and conventional HS steels: LC, BH, HSLA, solid solution strengthened (SSS),
- first-generation AHSS: DP, stretch flangeable SF, TRIP, CP and MS,
- second-generation AHSS: TWIP, lightweight steel with induced plasticity (LS-IP).

According to Grosman and Piela [32], the yield strength of HSS sheets is 650 MPa, and for UHSS steels, the yield strength is between 550 MPa and 1500 MPa. Individual steel grades differ in their strengthening mechanisms.

Car body panels are most often produced using conventional sheet metal forming methods, such as bending and deep drawing. Stamping dies consist of three basic parts: a punch, a die, and a blankholder [33,34]. In an SMF process, a thin piece of metal sheet is stretched by a stamping tool into the desired shape without wrinkling [35,36]. The basic problems limiting the sheet metal forming processes are the springback phenomenon [37] and friction conditions [33,38], which determine the surface quality of the drawpiece and the possibility of obtaining large deformations of sheet metal. In unit and small-batch production, it is economical to produce a car body using single point incremental forming methods, which enable forming of the metallic [39,40] or bimetallic [41] sheets.

The body of a typical passenger car consists of many parts, most often welded [42,43,44], self-pierce riveted [45,46,47], or clinched [48] together. Moreover, the flow drill screwing process is the optimal process to realise the single-side connection of metal sheets [49]. Car body components can also be formed from tailor-welded blanks joined using laser welding technology. The advantages of tailor-welded blanks (TWBs) in the automotive industry include reductions in cost, weight, and noise with simultaneous increases in dimensional accuracy and crashworthiness [50].

The steel industry is seeing unprecedented growth in automotive applications of Advanced High-Strength Steels (AHSSs). Independent marketing research [51] suggests that these are the fastest growing materials for future automotive applications. Industrial interest resulted in the continuation of the ULSAB project by scientific–industrial consortia in the projects: ULSAB—Advanced Vehicle Concepts [52], UltraLight Steel Auto Closure (ULSAC) [53], UltraLight Steel Auto Suspensions (ULSAS) [54], and FutureSteelVehicle (FSV) [55].

In parallel with the optimisation of the chemical composition and microstructure of steels, there has been an increased interest in metal-based laminates, which provide the required stiffness while being lighter. In addition to the commonly known layered composites based on aluminium alloy sheets, there has been an increased interest in hybrid structures combining steel sheets and a polypropylene core. Steel–polymer laminates (steel/polymer/steel) demonstrate high fatigue strength and impact resistance [56]. Examples are Bondal and Litecor sandwich materials. Bondal laminate (ThyssenKrupp Steel Europe) in a 0.5/0.5/0.5 mm configuration is used for damping applications [57]. Litecor laminates (ThyssenKrupp Steel Europe) consist of two layers of HX220YD interstitial-free steel sheets (0.2–0.5 mm) with an intermediate layer of polyamide/polyethylene (PA6—52 wt.%, PE—36 wt.% and 12 wt.% of other additives) [58]. Significant advantages of this class of materials are their improved acoustic and thermal damping properties. Hybrix, a laminate developed by Lamera AB, consists of steel or polyamide microfibres sandwiched between two sheet metals (stainless steel, carbon steel, or aluminium). This material is produced with a thickness of 0.5–3.5 mm [59]. Steel/polymer/steel composites can be subjected to deep drawing and bending [60,61]. Since this review is devoted to the use of metallic materials in the production of body panels and structural members, the properties and application of steel-based hybrid structures will not be discussed in the following chapters.

### 2.2. Conventional Low-Carbon Steels

The most important technological property of sheets intended for cold metal forming in various forming operations is formability. Formability is the ability of a sheet metal to undergo plastic deformation without being damaged. According to the EN 10130 standard [62], cold-rolled sheet metals from low-carbon steels are produced in six grades: DC01, DC03, DC04, DC05, DC06, and DC07. The basic mechanical properties of low-carbon steels for cold forming are presented in Table 1.

Conventional low-carbon (CLC) steels are supplied in the form of cold-rolled sheets or strips. Due to the rolling production method, the mechanical properties of sheets and strips are anisotropic. Due to their high plastic properties and tendency to strain hardening, they are processed in plastic forming methods such as roll profiling, deep drawing and stretching. Good plastic properties result from the very low carbon and nitrogen content in this group of steels. CLC sheets are characterised by very good weldability and can be joined by arc welding and pressure welding. These methods are the basic methods of joining sheets and components in the automotive industry. The advantage of this technique is the high speed of the process and the lack of need to make holes for riveting or screw connections. Although CLC sheets have lower strength than steels with higher carbon content, they exhibit sufficient strength for many structural applications, such as body panels and stringers. Due to their susceptibility to corrosion, CLC sheet metal components must be covered with anti-corrosion coatings.

A non-ageing DC06 steel is suitable for the most demanding deep-drawing processes [63]. DC04 and DC05 deep-drawing quality steels are suitable for deep drawing with increased requirements and stretch-draw forming. These grades of steel sheets are not subject to ageing and are intended to make structural elements that allow for the execution of a very complex shape. In the case of conventional deep-drawing quality steel sheets, as the strength properties increase, the formability decreases, making it difficult to form car bodies with complex shapes. The change in sheet metal formability is closely related to the carbon content. Meanwhile, for the steel with the lowest drawability (DC01), the carbon content is 0.12%, and for the steel with the greatest susceptibility to deep-drawing (DC07), the carbon content does not exceed 0.01%.

### 2.3. Complex-Phase Steels

The concept of the development of CP steels initially emerged from the idea of replacing martensite with bainite in dual-phase steels [64]. The microstructure of CP steel is characterised by refined grains. The yield strength of these steels is much higher than that of two-phase steels with the same tensile strength. Ferritic–martensitic–bainitic steels have a yield strength of up to about 800 MPa, a tensile strength of up to about 1000 MPa, and an elongation at break of at least 7% [65]. The fine microstructure of high-strength phases (bainite, martensite) increases the yield strength [66]. Promising strength properties are demonstrated by steels with a multi-phase microstructure containing carbon-depleted martensite, carbide-free bainite, and retained austenite [67]. Their high ability to absorb energy and their bendability mean that complex-phase steels are used to produce bumpers and B-pillar reinforcements [68]. These steels are also used to form side beams as a kind of reinforcement supporting plastic cover plates. The combination of high-strength steel and the damping properties of plastics proves useful in absorbing collision energy. Components exposed to fatigue failure are tested in stress-controlled high cycle fatigue tests to determine the fatigue limit. Chaurasiya et al. [69] found that the fatigue limit of AHSS steel sheets is much higher than that of lower-strength structural steel and has comparable ductility. Due to their homogeneous microstructure, CP steels exhibit superior stretch–flangeability [70]. Graux et al. [71] investigated the effect of process parameters on final mechanical properties and microstructure. The microstructure of hot-rolled bainitic steel obtained consisted of a homogeneous lath/granular bainite mixture and exhibited a hole expansion ratio exceeding 70% and a UTS of 830 MPa. The lower recrystallisation promoted bainite transformation and provided more nucleation sites for bainite nucleation [72].

### 2.4. Interstitial Free Steels

Interstitial free steels are a class of steel with increased plasticity defined by the anisotropy coefficient *r* ≥ 1.8 and the strain hardening exponent *n* ≥ 0.22 [73]. The interstitial elements in these steels combine with stabilising elements, mainly titanium and/or niobium, to form various types of carbides, nitrides, sulphides, carbonitrides, and carbon sulphides. Although IF steels meet the stringent requirements of the automotive industry in terms of formability, their main disadvantage is low tensile strength (R_m_ < 360 MPa). Adding 1.18 wt.% of Cu can achieve a yield strength of 456 MPa and a tensile strength of approximately 566 MPa by post-annealing aging [74]. The effect of adding amounts of niobium, titanium, and phosphorus on the mechanical properties and microstructure of IF steel was studied by da Rocha Santos [75]. It was found that in order to utilise the full potential for IF steel strengthening, they must have their rolling process closely controlled to mitigate the effects of solid solution carbon and excessive hardening. An increase in the formability of sheet metals is achieved by using a high-purity metal charge and technological methods for obtaining nitrogen and carbon contents in IF steels [76]. A significant increase in the ductility of cold-rolled IF steels annealed according to the continuous hot-dip galvanising unit regime by giving a favourable existence form to the interstitial elements and impurities [77].

IF steels are characterised by very good formability with a yield strength between 140 and 260 MPa. Reducing the nitrogen and carbon content in these steels is achieved by the addition of niobium and titanium, which bind interstitial elements into durable compounds. Increasing the phosphorus content in these steels allows obtaining sheets with a yield strength of 275–350 MPa [78]. Increased strength is achieved by increasing the content of phosphorus, manganese, and silicon. IF steel and Drawing Quality Special Killed (DQSK) steel are mild steels with high plasticity. These steels are characterised by a total elongation of 30–60% [23]. Accumulative roll bonding (ARB) followed by annealing treatment enables the enhancement of the strength of IF steels without compromising toughness and ductility [79]. However, as reported by Tsuji et al. [80], ARB-processed IF steel showed high strength but limited elongation. IF steels are suitable for producing particularly complex stampings that require high mechanical strength, such as inner wheel arches and boot lid reinforcements.

### 2.5. Bake Hardenable Steels

**Figure 2:**
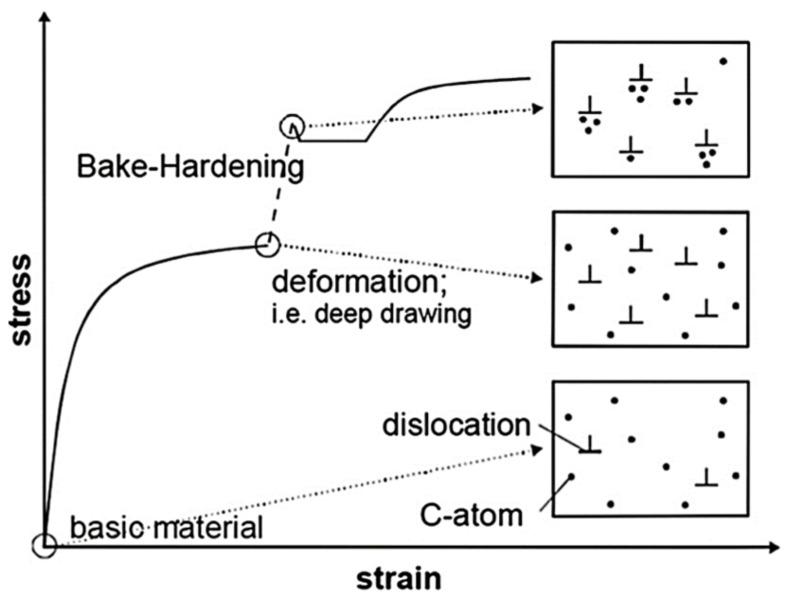
The influence of bake hardening on stress (reproduced with permission from Reference [78]; copyright © 2024 Elsevier Ltd. All rights reserved).

Bake hardenable steel in as-received state is characterised by good formability in cold forming conditions. After forming, the component is placed in an oven at a temperature of 150–250 °C for 15 min to harden. This increases the yield strength (Figure 2) as a result of the strengthening process. In BH steels, the strengthening is caused by the release of coherent carbides and the diffusion of free carbon atoms into dislocations. The steel strengthening process is a type of strain ageing causing the segregation of carbon and nitrogen atoms into dislocations generated during the sheet metal forming [78]. The bake hardening process is considered useful if the increase in yield stress is 40–60 MPa.

BH steels are used in the production of body parts that are required to be dent resistant. In addition to the phenomenon of strain hardening occurring during forming, car elements made of BH sheets (e.g., doors, boot lids) strengthen during bake hardening. These steels are solution hardened (Mn, P, Si), and the upper values of the yield strength obtained for sheets made of these steels are 320–450 MPa [81].

### 2.6. Dual-Phase Steels

**Figure 3:**
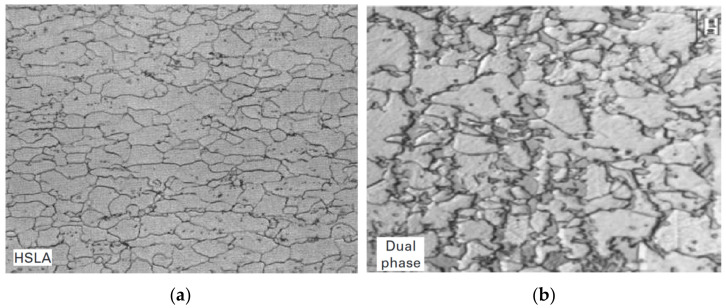
Typical microstructure of (**a**) HSLA and (**b**) DP steel (reproduced with permission from Reference [31]; copyright © 2024 Woodhead Publishing Limited. All rights reserved).

The microstructure of dual-phase steels includes ferrite as the matrix and hard martensite [82]. Martensite provides DP steel with high strength, and ferrite is responsible for its plastic properties. Reduced martensite content in steel may result in insufficient tensile strength and high yield strength [83]. The two-phase microstructure is obtained by annealing the sheet metal after cold rolling in the temperature range of ferrite and austenite occurrence. The structure of DP steels is formed by a ferrite matrix in which martensite (10–35%) and residual austenite (1–2%) are homogeneously dispersed [84]. Avoidance of the formation of pearlite or bainite is achieved by an appropriate rate of cooling of the sheet from the annealing temperature. During deformation, the unstable retained austenite transforms into martensite, thus increasing the ductility of the material. The difference in the microstructures of dual-phase and the precipitation hardened HSLA steels is shown in Figure 3. HSLA steel consists mainly of ferrite with the presence of fine and dispersed carbides of vanadium, titanium or niobium. However, the microstructure of DP steel generally contains 10–70% volume fraction of martensite and consists of irregular martensitic islands in a ferrite matrix. The higher the martensite content in DP steels, the greater their tensile strength [85].

DP steels do not exhibit a physical yield point. Two-phase steels are characterised by very good drawability; the ratio of yield strength to tensile strength of these steels is below 0.5. The strengthening of these steels during the bake hardening can reach 100 MPa [78]. Effect of forming strain on low cycle, high cycle and notch fatigue performance of DP steels has been discussed by Paul [86]. Llewellyn and Hudd [87] found that the optimum combination of strength and formability is obtained by a very fine distribution of martensite islands and a very fine ferrite grain size. Ding et al. [88] concluded that good deformation compatibility between lamellar martensite and lamellar ferrite increases strength and plasticity.

Ferrite–bainite steels are a variation of DP steel that combines ferrite with bainite as a second phase instead of martensite [89]. Bainite is a phase with lower strength than martensite; therefore, FB steels exhibit similar properties to ferritic–martensitic DP steels [89]. The tensile strength of ferrite–bainite steels is between 500 and 900 MPa [89]. FB steels were developed for edge-stretching applications due to the decreased likelihood of cracks forming in bainite during shearing operations [89]. DP steel has the largest share in the structure of a modern car (approximately 80%) [78].

DP grades are commonly designated by their tensile strength (e.g., the nominal minimum tensile strength of DP500 steel is 500 MPa [89]. The common range of DP grades is DP500 to DP1000 [89]. However, it is possible to obtain a strength of 1400 MPa [90]. DP steels with low carbon content are weldable. They increase safety in cars where they are used as seat guides, child seats, and windshield pillars [90]. DP steel is also ideal for use in complex structural components of light vehicles, such as car body panels and bumper reinforcements [83,90].

### 2.7. Transformation-Induced Plasticity Steels

Transformation-induced plasticity steels are first-generation steels with a fine-grained structure, which are characterised by high strength and high plasticity [91]. TRIP steels are defined as steels with increased ductility due to the phase transformation of retained austenite into martensite during the forming process [92,93]. The properties of TRIP steel are constituted by many strengthening mechanisms: solution, dispersion, work hardening, and phase transformation. The strengthening effect is based on the transformation of retained austenite into martensite during plastic deformation, which leads to a favourable combination of strength and plastic properties [94]. Transformation-strengthened steels obtain high strength because they contain a certain amount of transformation products such as martensite, bainite, and retained austenite. In order to ensure high ductility of the sheet material, austenite should be characterised by stability allowing its gradual transformation over the entire range of deformation during product formation. The factor that determines the stability of austenite is the carbon content in the steel. Austenite with a low carbon content can be completely transformed into martensite with only a small deformation. On the other hand, austenite with a very high carbon content is so stable that even large deformations will not cause its transformation. To stabilise the austenite at room temperature, the retained austenite is strengthened by dissolving the carbon and reducing the grain size, thereby avoiding the formation of martensite during cooling. Krizan [95] linked the effect of increasing strength in TRIP steels with the formation of additional mobile dislocations in ferrite in adjacent areas of deformed martensite.

The main components of multiphase TRIP steels are carbon and manganese (usually approximately 1.5 wt.%). Manganese promotes an increase in the content of retained austenite in steel. Multiphase TRIP steels containing ferrite, martensite, bainite, and retained austenite show even better formability than DP steels. Aluminium and silicon limit the release of carbides and cementite during the formation of bainitic ferrite, thus promoting the enrichment of austenite in carbon. An increase in ductility due to transformation is achieved in all steels containing metastable austenite, which undergoes a martensitic transformation during deformation [78]. The enrichment of austenite with carbon occurs during heating in the two-phase range and during the transformation of austenite into bainite. Too long annealing causes a decrease in the volume fraction of austenite in the steel at ambient temperature due to the increase of volume fraction of austenite transforming into bainite [78]. TRIP steels are characterised by high impact absorption energy; therefore, their typical applications are front longitudinal beams, A-pillar and B-pillar reinforcements, and in the crash zones of the car for their high energy absorption [96].

### 2.8. Twinning-Induced Plasticity Steels

The chemical composition of twinning-induced plasticity steels, which show mechanical twinning induced by deformation, is characterised by a high manganese content (between 15% and 35%). TWIP steels also typically contain 0.5–1 wt.% C [97]. Nickel and manganese are mainly used to obtain retained austenite in a microstructure even at room temperature. Additionally, the silicon content in the amount of 2–4% and/or aluminium gives the steel an austenitic microstructure, ensuring its high ductility and susceptibility to deep drawing [98]. These steels exhibit a unique combination of tensile strength and ductility (e.g., at tensile strengths above 1000 MPa, the material can exhibit ductility of up to 50%) [99]. TWIP steels have a regular face-centred cubic (FCC) structure with low stacking fault energy [1]. The properties of TWIP steels are related to the main mechanism of plastic deformation—twinning [100].

**Figure 4:**
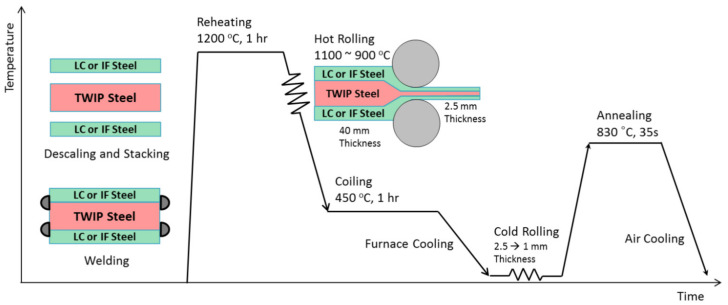
Fabrication procedure of the cold-rolled LC/TWIP/LC and IF/TWIP/IF sheets (reproduced with permission from Reference [102]; copyright © 2024 Elsevier B.V. All rights reserved).

Quenching and tempering of 35CrSiMn5-5-4 and 30NiMnSiCr7-5-4-4 steels involves the creation of an ultra-fine-grained, multi-phase microstructure consisting of carbide-free bainite with retained austenite in a ferritic matrix, which was the basis for the creation of (Ultrafine-Grained Transformation Induced Plasticity (UFG-TRIP) steels [101]. TWIP-cored three-layer steel sheets containing thin surface layers of low-carbon (LF) or IF steel can be fabricated by solid-state hot-roll bonding (Figure 4) followed by cold rolling [102]. TWIP-cored sheets cover a wide range of ductility levels required in automotive steel sheets by controlling the volume fraction of the TWIP-cored region.

The second generation of advanced TWIP steels is characterised by increased work hardening intensity and very high plasticity. The dominant deformation mechanism in these steels is twinning [103]. TWIP steel is a fully austenitic steel with high aluminium, manganese and carbon content. These steels are characterised by very high mechanical strength with very high drawability. As a result of the dynamically induced twinning mechanism, a very high strain hardening capacity can be achieved. This feature makes TWIP steel an excellent material for energy-absorbing elements and structurally responsible components with complex shapes [103].

### 2.9. Triplex Steels

Triplex steels have considerably huge amounts of carbon (up to 1.3%) and manganese (up to 30%) [104]. However, the amount of manganese should be less than 35% in order to avoid formation of brittle β-Mn. In austenite + ferrite + κ-carbide Triplex steel, the manganese content varies in medium to high levels, and the lightweight effect is 10.5% (in comparison with pure Fe) [105]. Triplex steels belong to the group of high-manganese steels with different shares in the structure of three phases: high-alloy austenite, high-alloy ferrite, and carbide precipitates, including dispersion carbides κ-(Fe,vMn)_3_AlC [106] responsible for the very good mechanical properties of these steels. The precipitation of M_3_C-(Fe, Mn)_3_AlC nanocarbides (so-called κ carbides) in this type of steel is influenced by the addition of Al (>5 wt.%). Triplex steels offer tensile strengths from 870 MPa to 1100 MPa with total elongation in the range of 25–70% [107]. They are also characterised by a specific density of 6400–7100 kg/m^3^, which is at least 10% lower compared to conventional steel grades (~7850 kg/m^3^). With increasing Al content, Fe–Mn–Al–C steels achieve high specific strength due to the combination of low density and intense work hardening [108]. The mechanical properties of these steels are determined primarily by the morphology of κ-(Fe, Mn)_3_AlC carbides, which may cause the steel to become brittle during cold plastic deformation at the grain boundaries in the form of large particles [106]. The advantage of Triplex steels in the automotive industry is their strength–elongation compromise coupled with a low density. The austenite stability and stacking fault energy determine the hardening capacity of low-density steels [109]. An outstanding strength–ductility balance of Triplex steels results from an optimum percentage of carbon, aluminium, and manganese [110,111]. The carbon content is crucial to achieving the outstanding comprehensive performance of Cr-containing low-density triplex steels [112].

### 2.10. Martensitic Steels

Martensitic steels (MSs) use the phenomenon of martensitic transformation that occurs when austenite reaches the initial transformation temperature (M_s_). After cooling, these steels are subjected to martensitic tempering to increase their formability. Steel with a higher martensite content shows greater strength. The disadvantage of this group of steels is the relatively low elongation. The tensile strength of this group of steels reaches 1700 MPa with an elongation not exceeding 10% [113]. Martensitic steels are commercially available with various tensile strengths ranging from 980–1700 MPa [114].

Hydrogen embrittlement (HE) is a potential issue for martensitic steels in auto service. The hydrogen embrittlement sensitivity of UHSSs is influenced by the concentration of diffusible hydrogen, stress, and strain hardening resulting from the cold forming process. Sub-critical cracking is associated with a reduction in the strength, toughness, and ductility of MS steel [115,116]. Sub-critical crack extension by HE includes the following mechanisms: hydrogen-enhanced strain-induced vacancies [117], hydrogen-enhanced decohesion [118], hydrogen-enhanced plasticity-mediated decohesion [119], adsorption-induced dislocation emission [120], and hydrogen-enhanced localised plasticity [121]. Tong et al. [122] proposed a method of determination of the critical conditions for a safe service and rapid evaluation of the hydrogen embrittlement sensitivity of martensite steel MS1500.

MS steel components have the highest potential for absorbing impact energy and are used for bumpers, door beams, the protective cage around passengers, including front and rear bumpers, roof cross members, and protection zones for electric vehicle batteries [123].

### 2.11. Press-Hardened Steels

Press-Hardened (PH) steels are typically carbon–manganese–boron alloyed steels that were developed in the mid 1980s for the automotive body in white construction [124]. A small amount of boron (~0.002 wt.%) is used to facilitate the quenching process. These steels are strengthened at the hot stamping stage, and their ultimate tensile strength reaches 2000 MPa (yield stress up to 1380 MPa). The hot-forming process is mainly divided into two different approaches, indirect and direct [125]. PH steels are also known as Hot Formed (HF) steels or hot press forming (HPF) steels. Heat treatment of these steels is a complex process and involves forming and tempering. The first stage of processing is full austenitisation by heating to a temperature of 880–950 °C. The workpiece is cooled down rapidly in the tool, applying the critical cooling rate (25–30 °C/s). It should be noted that HPF steels have a ferritic–pearlitic microstructure in as-received state. PH steels are characterised by very low springback and are suitable for forming components with complex shapes. Components formed by PH steels exhibit multi-strength performance (e.g., energy transfer and energy absorption) via tailored tempering [125]. Typical applications of PH steels include door reinforcements, A-pillar and B-pillar reinforcements, roof panels, and door and sill reinforcements.

### 2.12. Quenching and Partitioning Steels

Quenching and partitioning (QP) steels belonging to the third generation of AHSS exhibit a good combination of formability and strength [126]. These steels are based on the QP process which was first proposed by Speer et al. [127]. The QP steels contain manganese between 1.5 and 2.5%, carbon between 0.15 and 0.4%, and around 1.5 wt.% of Al + Si [128]. The addition of other alloying elements, such as phosphorus, aluminium, and silicon, plays an important role by delaying the carbide formation [129,130].

The microstructure of commercially available QP steels consists of martensite (50–80%) formed during hardening, ferrite (20–40%) formed during slow cooling of austenite and dispersed retained austenite (5–10%). The main components of QP steels are martensite and residual austenite, and ferrite may be present in some of these steels. High-strength QP steels have a reduced amount of ferrite [131]. The morphology, amount, and stability of retained austenite depend on the hardening temperature, partitioning temperature and partitioning time. During the plastic processing of components, austenite is transformed into newly formed martensite through the TRIP effect, thus increasing the strength and ductility of the steel.

**Figure 5:**
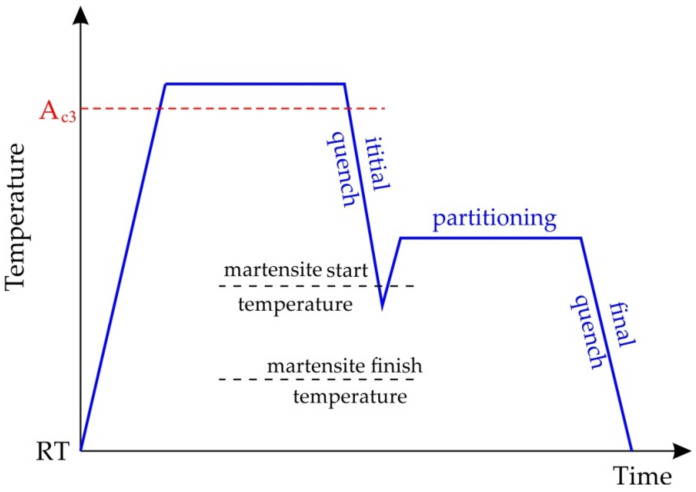
Thermal cycle for the quenching and partitioning process (A_c3_—transformation temperature, RT—room temperature), prepared with permission from Reference [132] (copyright © 2024 Acta Materialia Inc. Published by Elsevier Ltd. All rights reserved).

QP steels contain retained austenite which allows a significant energy absorption during deformation via the TRIP effect. The scheme of the thermal cycle of heat treatment of QP steel is shown in Figure 5 [132]. After first quenching from a fully austenitised or intercritical annealing temperature, the steel can be reheated to a higher temperature and then quenched to room temperature. Carpio et al. [133] observed a significant increment in the retained austenite at an increasing partitioning temperature.

Typical QP steels are QP980 and QP1180 [132]. The first application of QP980 was carried out in Chevrolet Sail (2016) [134]. In 2021, hot dip galvanised QP980 was used in five components of the front and rear floor assemblies in Ford Bronco [135].

### 2.13. Stainless Steels

Stainless steel (SS) is an alloy of iron and carbon containing up to 1.2 wt.% of carbon and at least 10.5 wt.% of chromium. Iron-carbon alloys with a concentration above 13 wt.% of chromium tend to create passive layers with a tendency to self-rebuild, providing steel corrosion resistance. Corrosion-resistant steels included in the EN 10088-1:2014 [136] standard include several dozen grades divided into the following groups: ferritic stainless steels, austenitic stainless steels, martensitic and precipitation-hardened stainless steels, ferritic-austenitic stainless steels, heat-resistant ferritic steels, heat-resistant austenitic steels, high-temperature creep resistance martensitic steels, high-temperature creep resistance austenitic steels.

**Figure 6:**
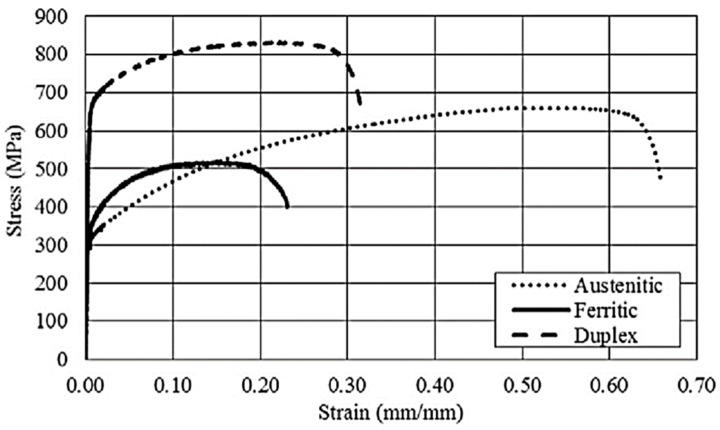
Comparison of tensile curves of selected corrosion-resistant steel families (reproduced with permission from Reference [139] (copyright © 2024 Elsevier Ltd. All rights reserved).

The main advantageous property of stainless steels, in addition to corrosion resistance, is a favourable strength-to-density ratio [137]. Stainless steels are susceptible to plastic forming and exhibit strain hardening phenomena, but their properties may vary significantly between corrosion-resistant steel families (Figure 6). The austenitic steel group provides more than half of the world’s demand for stainless steels. Austenitic steels are characterised by high susceptibility to plastic deformation and rather low strength, while martensitic steels show high strength and low ductility [138]. Particularly attractive is the high impact strength of austenitic stainless steels, which are used for elements of car crumple zones [139].

The density of stainless steels is similar to the density of conventional steels; therefore, replacing carbon steel with stainless steel does not bring any benefits that reduce the weight of vehicles. However, the strength to density ratio of SS steels is higher than HSS steels; they are more susceptible to deformation and absorb more energy on impact. High corrosion resistance means that they do not require additional anti-corrosion coatings. In passenger cars, stainless steel is used primarily in exhaust systems and fuel tanks, where the material’s resistance to corrosion and oxidation is required. Moreover, SSs have been successfully used on body structure reinforcements, dashboard casing (Porsche), front cross members (Audi), vertical pillars, and car seat elements.

Stainless steel is very rarely used for passenger car body parts due to its high price. In the case of public transport vehicles (buses, trams) and passenger trains, which are heavily used vehicles, the bodies are made of stainless steel [140,141]. The high cost of producing stainless steel is related to the content of expensive alloying elements (including chromium and nickel). In the long term, despite the high price, stainless steel has an advantage over conventional carbon steel.

Particularly for hydrogen fuel cell vehicles, an important condition for safety is the reliability of the vehicle-mounted hydrogen container. Nam et al. [142] examined the high-manganese steels for hydrogen-related properties. In a low-temperature hydrogen environment, materials can be affected by hydrogen embrittlement and low-temperature embrittlement (LTE), which involves the weakening of materials exposed to low temperatures. Moreover, ultra-high-strength and low-carbon martensitic steels exhibit relatively low fracture toughness and are susceptible to low-temperature embrittlement that may lead to sudden accidents [143,144]. It is well known that grain boundaries can act as obstacles for cleavage crack, and LTE can be suppressed by grain refinement [145,146]. According to Nam et al. [142] low temperature mechanical properties of steels have barely been reported. Table 2 summarises the applications of the most common advanced high-strength steels used to produce the body frames and structural members.

## 3. Aluminium and Aluminium Alloys

### 3.1. Characterisation of Aluminium and Aluminium Alloys

Aluminium is a metallic element with a density of 2698.9 kg/m^3^ at a temperature of 20 °C. The strength-to-weight ratio of aluminium alloys is greater than that of steel. It is distinguished by a comparatively lower density, measuring 2.7 g/cm^3^, in contrast to 7.9 g/cm^3^ for steel. It exhibits a propensity for malleability, along with notable electrical and thermal conductivity, as well as a remarkable resistance to corrosion. In coastal regions or other corrosive settings, sheet metal undergoes an electrolytic oxidation process despite its inherent propensity to develop a naturally oxidised coating on its surface.

Furthermore, it is noteworthy that the ductility of these materials is unaffected by lowering temperatures, resulting in better ductility compared to steel under low-temperature conditions. Aluminium alloys are experiencing growing usage in construction. This trend allows for maintaining equivalent strength while reducing the structure’s weight by approximately 50% compared to steel materials [206].

Aluminium alloys may be classified into two main categories: cast and wrought. The composition of the substance is represented by a numerical code consisting of four digits, which indicates the primary impurities present and, in some cases, the degree of purity. Placing a decimal point between the last two numbers is customary in cast alloys. After the numerical sequence, a hyphen is present, followed by the essential temperature classification. This classification consists of a letter and, potentially, a numerical value ranging from one to three digits. This designation signifies the specific mechanical and/or thermal treatment implemented on the alloy.

The basic designations for aluminium alloys are as follows [207]:

- F—as fabricated
- O—annealed
- H—strain hardened (cold worked)
- W—solution heat treated
- T—thermally treated

The first digit after ‘H’ identifies the basic condition:

- H1—strain hardened only
- H2—strain hardened and partially annealed
- H3—strain hardened and stabilised
- H4—strain hardened and painted

The ‘T1–T10’ designations are applied to those alloys that are age hardened. Details of the designations can be found in [207].

Forming aluminium alloy sheets at elevated temperatures is a challenging thermal-mechanical process, with friction conditions being a crucial factor affecting forming quality and tool life [208]. High-temperature (warm) forming is a common method for shaping aluminium alloys, involving the heating of input material and extrusion with hot tools. Wrought aluminium alloys that are not heat-treatable can be classified into one of three groups listed in Table 3 based on standard designations provided by the Aluminium Association [209].

### 3.2. Aluminium Alloy Families

Aluminium alloys are categorised into eight series, 1xxxx-9xxx, based on their chemical composition, as specified by the EN 573-3:2005 [210] standard. Table 4 displays a compilation of specific characteristics of wrought aluminium alloys.

Due to their advantageous characteristics, aluminium and its alloys have gained appeal for use in the aerospace and automotive sectors. Aluminium alloys used in the fabrication of automobile body components consist mainly of 5xxx-series (Al-Mg) and 6xxx-series (Al–Mg–Si), with a minor presence of 2xxx-series (Al-Cu) and 7xxx (Al-Zn-Mg-Cu). Alloys in the 5xxx series are valued for their excellent strength-to-weight ratio, high formability, and full recyclability. Meanwhile, the 6xxx series is versatile, amenable to heat treatment and welding, and exhibits excellent plasticity. Consequently, the 6xxx series alloys presently account for at least 80% of the aluminium alloys used by automotive manufacturers [212].

The addition of alloying elements into aluminium significantly enhances its strength qualities, sometimes resulting in a multiple-fold increase [213]. The Al-based alloys formed using metal forming processes have a low density and a high impact strength. Various elements, including molybdenum, magnesium, cobalt, manganese, tungsten, vanadium, nickel, titanium, copper, iron, zinc, and silicon, have been identified as significant contributors to the enhancement of aluminium hardness [214]. The inclusion of nickel and cobalt, together with magnesium and manganese, has been shown to enhance the strength characteristics of the material. Additionally, the presence of titanium and chromium has been found to influence the grain refining process [215]. The presence of copper has been shown to mitigate casting shrinkage. There are two distinct categories of aluminium alloys: casting alloys, denoted as [211], and wrought alloys, marked as [210]. The concentration of primary alloying elements in casting alloys may reach a maximum of 30 wt.%, but it typically ranges to around 10 wt.% in wrought alloys. Wrought alloys generally consist of alloying elements comprising a maximum of 5 wt.% and are often used in a form that has been strengthened and heat-treated. Cast aluminium alloys have the potential to undergo metal-forming techniques under certain circumstances [216]. Certain alloys can be used in both cast and wrought forms.

**Figure 7:**
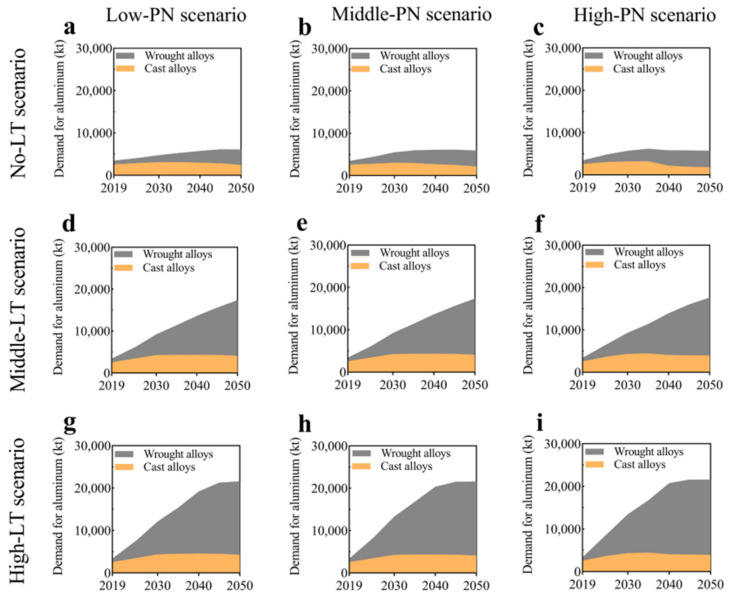
Aluminium alloy-specific Chinese automotive demand estimates. Top to bottom, the three rows show No-LT (**a**–**c**), Middle-LT (**d**–**f**), and High-LT (**g**–**i**). Three columns show Low-PN, Middle-PN, and High-PN situations from left to right. Where LT = light-weighting-trend and PN = penetration scenario (reproduced with permission from Reference [218]; copyright © 2024 The Author(s). Published by Elsevier B.V.).

Modern aluminium alloys are extensively used in various structural components within the aviation and automotive sectors because of their notable attributes, including a high power-to-weight ratio, cheap cost, and exceptional wear resistance [217]. Wang et al. [218] conducted a material flow analysis to identify the secondary aluminium flows in China, specifically focusing on the classification of these flows based on alloy type. The proportion of wrought alloys in secondary aluminium production is projected to decline from 30% in 2019 to 4% in 2050, assuming the current recycling system remains unchanged. Additionally, the study suggests that it is possible to recycle 87–183 kt of wrought alloys derived from End-of-Life Vehicles by enhancing the processes of collection, dismantling, and sorting. It is anticipated that there may be a potential decrease in primary aluminium ingot usage ranging from 10% to 37% throughout 2019 to 2050. In 2019, the automotive industry used 2599 kilotons of cast alloys and 870 kilotons of wrought alloys (see Figure 7). In the end, the study’s findings indicate an anticipated increase in the utilisation of cast alloys in the automotive industry, irrespective of the progress made in electric vehicle technology.

Automobile companies such as Volvo replaced stamped and extruded sheets into single castings, also known as ‘mega-giga casting technology’ (MGCT) [219]. Tesla, in future model Tesla Y., used MGCT to produce two parts instead of 171 parts. Volkswagen plans to speed up production including potential mega-casting solutions under the “Project trinity” [220]. The share of aluminium alloys in the overall weight of an average car constantly increases from 35 kg in the 1970s to 152 kg in 2023, and it is expected that by 2025, the Al-based alloy content in typical cars will reach 250 kg [221]. As a result of physical and mechanical properties, car body structures are most commonly made from aluminium alloys belonging to the following groups: 5xxx, 6xxxx, and 7xxx [222].

### 3.3. 1xxx-Series Aluminium Alloys

The predominant applications of 1xxx-series aluminium alloys encompass the production of packaging foil and strips, chemical equipment, tank car or truck bodies, spun hollowware, and intricate sheet metal work, primarily due to their commendable attributes of high corrosion resistance and formability [223]. The distinguishing features of 1xxx alloys revolve around their exceptional corrosion resistance, suitability for constructing chemical tanks and piping, and notable electrical conductivity for applications such as bus bars. However, it is pertinent to acknowledge that these alloys exhibit comparatively limited mechanical properties [224]. Floor components and structural components are fabricated from EN AW-1100 aluminium alloy, while EN AW-1050, EN AW-1100, and EN AW-1200 are used to produce heat insulators.

### 3.4. 2xxx-Series Aluminium Alloys

The 2xxx-series comprises aluminium alloys that include copper as the primary alloying element [209], often in concentrations of up to around 2 wt.%. These alloys are distinguished by their relatively high strength; nevertheless, including copper renders them more susceptible to corrosion. Typically, sheet metal products undergo a cladding process whereby they are coated with high-purity aluminium. Alloys of the 2xxx series, such as the widely used EN AW-2024 alloy, provide advantageous properties for machining processes. The set of alloys above finds use in several sectors, such as automotive, military, and aviation industries, where they are utilised in sheet metal structural components. The uses of 2xxx-series aluminium alloys in the automotive industry are presented in Table 5.

### 3.5. 3xxx-Series Aluminium Alloys

The aluminium alloys of the 3xxx series are well recognised for their exceptional formability, resistance to corrosion, and mild strength. The primary composition of this series mainly comprises aluminium-manganese alloys, whereby manganese serves as the principal alloying element, often falling within the range of 1.0 wt.% to 1.5 wt.% [229]. The alloys belonging to the 3xxx series exhibit a moderate level of resistance to corrosion, especially when exposed to marine and chemical conditions. The use of manganese significantly improves its capacity to withstand air corrosion. These materials have favourable weldability characteristics, rendering them appropriate for a range of welding methodologies including gas metal arc welding (GMAW) and resistance welding. These alloys are used in producing cookware and cooking equipment due to their malleability and ability to resist corrosion [207]. The 3xxx alloys are often used in heat exchanger applications due to their high thermal conductivity. Air conditioning systems often use alloys from the 3xxx series as constituent materials. The alloy that is most often seen in the 3xxx family is EN AW-3003, which finds extensive utilisation across several applications, particularly in the field of architecture [230]. These alloys provide favourable formability characteristics, rendering them appropriate for various shaping techniques, including rolling, extrusion, and drawing. The 3xxx-series Al-based alloys are recognised for their exceptional formability, good corrosion resistance, and satisfactory strength level. The uses of Al–Mn–Mg alloys in the automotive industry are presented in Table 6.

### 3.6. 4xxx-Series Aluminium Alloys

The 4xxx series of aluminium alloys is distinguished by including silicon as the principal alloying element, typically in the range of 4.5–6.0 wt.%. The most frequently used alloys, in addition to silicon, also contain additions of nickel, magnesium and copper, thanks to which they can be subjected to supersaturation and ageing, which increase their strength. The eutectic mixture in alloys containing 11.6% Si is formed from coarse acicular crystals consisting of a β solution with the addition of α solid solution crystals. Hypoeutectic alloys contain, in addition to the α solid solution, the precipitates of the eutectic mixture (α + β). However, in hypereutectic alloys, primary precipitates of β phases appear in the eutectic. Hypereutectic silumins improved with phosphorus are characterised by α + β eutecticity with a small dispersion of β solution. The group of 4xxx-series alloys includes wrought alloys and cast alloys. Moreover, the 4xxx series consists of non-heat-treatable and heat-treatable alloys.

The alloy that stands out the most in this series is EN AW-4043. The 4xxx-series aluminium alloys are renowned for their exceptional welding properties and are extensively used in situations where the ability to be welded is of utmost importance, mainly for welding and brazing electrodes and brazing sheets [233]. The use of silicon in welding materials has been shown to improve fluidity and mitigate cracking, making them well-suited for a wide range of welding techniques. The 4xxx-series alloys have recently been eagerly used to produce Li-ion battery compartments (Audi E-tron [234]). The uses of 4xxx-series aluminium alloys in car body structures are presented in Table 7.

### 3.7. 5xxx-Series Aluminium Alloys

The series of alloys known as 5xxx, which mainly consist of magnesium as the principal alloying element (ranging from 0.2 wt.% to 10.6 wt.%), has good resistance to corrosion and is also susceptible to plastic deformation and anodisation processes. The 5xxx-series aluminium alloys are supplied to automotive companies in an annealed condition characterised by a recrystallised grain structure influenced by insoluble Fe-based intermetallics and dispersoids. The crystallographic texture of these alloys has little effect on their formability. However, magnesium content and grain size are considered to be the main factors influencing the formability and strength of 5xxx-series alloys [246].

Aluminium alloys belonging to the 5xxx group find use in many sectors, such as shipbuilding, construction, chemicals, and rail vehicle production. Alloys with a magnesium concentration over 3.5 wt.% and operating at temperatures beyond roughly 65 °C are more susceptible to experiencing corrosion cracking [211]. Among the 5xxx-series alloy aluminium sheets, EN AW-5083, EN AW-5182, and EN AW-5754 aluminium sheets are commonly used in automobile manufacturing [247]. The 5xxx-series alloys are mainly used in body-in-white applications [248]. The uses of 5xxx-series aluminium alloys in car body structures are presented in Table 8.

### 3.8. 6xxx-Series Aluminium Alloys

The 6xxx series of aluminium alloys is distinguished by the incorporation of magnesium and silicon as the principal alloying constituents, with magnesium playing a predominant role in enhancing the strength of the alloy [230]. In the 6xxx aluminium alloys, the contents of Si and Mg are in the range of 0.5–1.2 wt.%, and the addition of these two alloying elements is done in the proper ratio of 1.73 to form an Mg_2_Si valence compound which is required for the formation of Mg_2_Si phase [230]. There are three strengthening methods of 6xxx-series aluminium alloys: grain-boundary strengthening, precipitation strengthening, and work hardening [230]. According to Baruah and Borah [255], the strengthening of Al–Mg–Si alloys is carried out in three steps: solution heat treatment, quenching and precipitation hardening or artificial ageing. The presence of magnesium excess increases the corrosion resistance but reduces formability and strength, while excess Si produces higher strength and higher formability [230]. According to the conventional theory of precipitation hardening in the 6xxx series of aluminium alloys, the hardening occurs via the precipitation and growth of Mg_2_Si [256]. Manganese is added to form α-AlMnSi dispersoids, which improve recrystallisation resistance and elevated-temperature strength [257].

The mechanical properties of typical precipitation hardening 6xxx-series alloys are characterised by the precipitation of hardening phases during production. Therefore, in order to obtain the desired crystallographic texture and grain size, it is necessary to control the processes of formation of precipitations and dispersions [246]. The Mg_2_Si phase is generated first since its forming free energy is lower than those of other precipitating phases [258]. In 6xxx-series alloys, the strengthening effect is usually achieved through β′ and β″ precipitates representing metastable variants of the equilibrium Mg2Si phase [257,259]. Work hardening increases the strength of these alloys. Additional dislocations are created during processing, and further clusters are formed during the hardening process. Al–Mg–Si alloys are characterised by the presence of the Q phase, which is stable only as a quaternary compound [260]. During artificial aging, metastable Q′-precipitates causing an additional strengthening effect [257]. The combination of precipitation hardening and work hardening during forming and paint curing is responsible for the increase in the strength of 6xxx-group alloys [246]. Recrystallisation in these alloys occurs simultaneously with the solutionising step. The formation of large sheet panels is facilitated when the sheet has isotropic properties closely related to the crystal allographic texture and grain shape. As shown by Burger et al. [246], the dependence of strength on grain size is very weak. In 6xxx-series alloys, control over the recrystallised microstructure obtained after solution heat treatment is achieved by using the effect of the distribution of soluble precipitates. In general, the iron content in Al–Mg–Si alloys adversely affects the mechanical properties and corrosion resistance by creating brittle β-AlFeSi lamellar phases in the microstructure [261,262]. However, a certain number of α-Fe dendrites can delay the propagation of microcracks and improve the tensile strength at elevated temperatures [263]. Wu et al. [261] stated that an iron content lower than 0.3–0.4% improves the mechanical properties of the aluminium alloy.

The utilisation of 6xxx-series aluminium alloys in diverse structural applications is extensive owing to their exceptional amalgamation of strength, weldability, and corrosion resistance [264]. One of the noteworthy alloys in this series is EN AW-6061, renowned for its adaptability and extensive use in extrusions, automotive components, and structural applications. The formability indexes of aluminium alloy sheets in the annealed state, specifically EN AW-6082, as presented in [265], suggest their suitability for various plastic forming processes.

**Figure 8:**
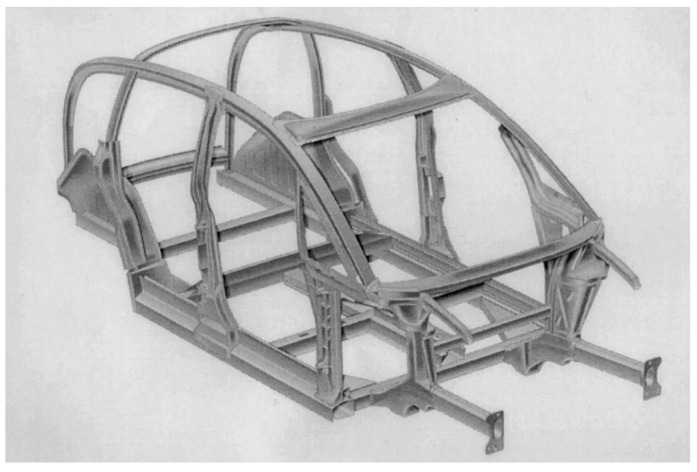
Audi AL2 aluminium body structure (reproduced with permission from Reference [251]; copyright © 2024 Elsevier Science S.A. All rights reserved).

The study by Rochet et al. [266] focused on investigating the deformation behaviour of an Al–Mg–Si aluminium alloy by implementing a two-pass equal-channel angular pressing (ECAP) technique. Using the ECAP technique resulted in notable enhancements in the alloy’s microstructure. These improvements included the fragmentation of more significant intermetallic compounds rich in iron (Fe-rich IMCs), a decrease in grain size, and an increase in the density of high-angle grain boundaries (HAGBs). Nevertheless, the results of the corrosion tests indicated that pitting corrosion was seen in all scenarios. However, it was observed that the samples subjected to ECAP had a higher number of pits, although shallower in depth. This observation may be attributed to the impact of the modified microstructure on the propagation of corrosion. Miller et al. [251] provided many instances of aluminium-intensive vehicles that included aluminium body components. The Audi A8 is equipped with an aluminium space frame, resulting in a notable reduction of 40% in the overall weight of the vehicle’s body. The object’s aluminium components weigh 385 kg, including 125 kg of sheet products, 70 kg of extrusions, 150 kg of castings, and 40 kg of miscellaneous aluminium forms. Similarly, the Ford AIV utilises a body structure composed of stamped aluminium, leading to a reduction in weight of 200 kg. This weight reduction is attributed to a decrease of 145 kg in the body structure itself and 53 kg in the closing panels. The Honda NSX has a body structure and outside panels manufactured using stamping. This process involves the shaping of the materials by the application of pressure. In the case of the NSX, around 210 kg of aluminium have been used for this purpose. About 100 kg is allocated for chassis components, while the remaining 130 kg is dedicated to various engine and drivetrain components. Several firms, including Chrysler, Reynolds Metals, Renault, Lotus, Jaguar, and GM, have developed prototypes and concept automobiles that prominently include aluminium components (see Figure 8 for Audi AL2 aluminium body structure).

Oana et al. [267] investigated the impact of the chemical composition of the additive material on the macroscopic and microscopic characteristics, as well as the primary defects, in heterogeneous welded joints in 6xxx-series aluminium-based alloys (EN AW-6063 and EN AW-6082). Different quantities of porosities are generated for filler materials under the given laser welding settings. Macro- and microcracks and porosities were observed in the welded root area of weld seams formed using AlMg5 and AlMg5Cr filler alloys. According to the findings obtained from experimental investigations, it is suggested that AlSi12 exhibits superior characteristics as a filler material for the laser beam welding process of 6xxx-series alloys. To sum up, the basic applications of 6xxx-series alloys include extruded components, body-in-white constructions [251,268] and body structures (sheet metals) [251]. The use of 6xxx series aluminium alloys in the automotive industry is presented in Table 9.

### 3.9. 7xxx-Series Aluminium Alloys

The 7xxx series of aluminium alloys is well recognised for its remarkable strength, making it highly suitable for situations that require high strength and lightweight properties [264]. The primary method of strengthening these alloys involves the incorporation of zinc as the principal alloying element [209]. In contrast, copper and minor quantities of other elements contribute to the qualities of the alloy. One of the notable alloys within this category is EN AW-7075, which is well-recognised for its outstanding ratio of strength to weight and extensive use in the aerospace industry.

The 7xxxx-series alloys contain the T phase (Al_2_Mg_3_Zn_3_), η, M phases (MgZn_2_) and the β phase (Al_3_Mg_5_), which have the ability to be precipitated and solubilised. The addition of Mg to AlZn alloys could greatly increase the mechanical strength of this group of alloys. The S-strengthening phase (CuMgAl_2_) can increase the strength of the alloy when the copper content is higher than the magnesium content and at the same time, Zn/Mg > 2.2 [273]. The MgZn_2_ precipitate is the compound responsible for the age hardening [215]. Magnesium/zinc mass ratio in the range of 5:2–7:1 can refine the precipitates and improve the strength of the 7xxx-series alloys [274]. The addition of manganese creates Al_6_Mn particles, which improves the stress-corrosion resistance of the 7xxx-series Al-based alloys [275]. However, excessive manganese content would lead to the emergence of the Al_20_Cu_2_Mn_3_ compound, which reduced strengthening phases in this group of aluminium alloys [276]. The increase in copper content favours the increase in the density of the precipitated phase. At the same time, the potential difference between the grain and grain boundary can be reduced [273]. The addition of zirconium (0.1–0.15%) can form an Al_3_Zr dispersion phase with a significant strengthening effect [277]. Reduction of the size of eutectic compounds and refining the secondary dendrites of the alloy can be achieved by the addition of yttrium. Such compounds as Y_12_Al_3_Zn and Al_3_Y as the core of heterogeneous nucleation have an obvious effect on grain refinement and alloy strengthening. The addition of 0.15% Y and 0.25% Er increases the dissolution temperature of the eutectic compound and enables the formation of fine-grained microstructure [278]. A comprehensive review of micro-alloying on the properties of 7xxx-series aluminium alloys can be found in [274].

**Figure 9:**
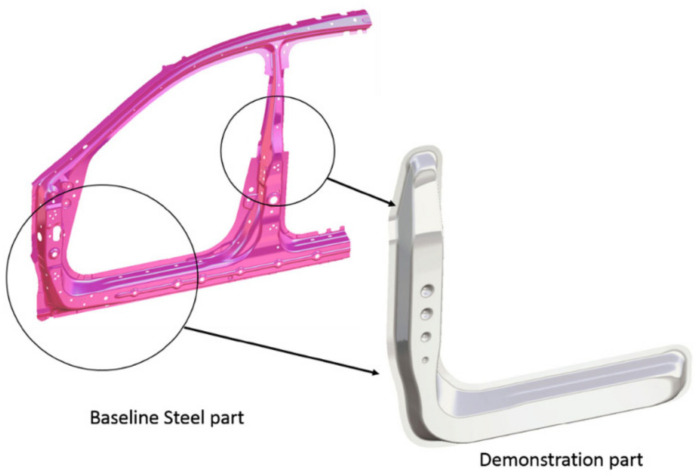
Baseline part and demonstration part geometry (reproduced with permission from Reference [279]; copyright © 2024, The Minerals, Metals & Materials Society).

**Figure 10:**
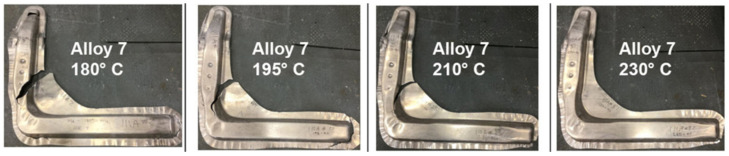
Forming trial results on 7xxx alloy at various forming temperatures (reproduced with permission from Reference [279]; copyright © 2024, The Minerals, Metals & Materials Society).

Through collaboration with industrial partners and with the help of the Department of Energy, Long et al. [279] have successfully created a number of experimental 7xxx-series alloys specifically designed for use in the automobile industry. The objective was to facilitate the creation of intricate structures at temperatures that do not exceed 225 °C. A prototype has been developed to showcase the difficulties encountered during the formation of a hot-stamped door ring component. The installation of tooling for this particular component has been completed inside a press line, and ongoing forming experiments are being conducted to assess the formability, strength, and corrosion resistance of the alloys [279] (Figure 9 and Figure 10). The optimum forming temperature for 7xxx alloys is 250 °C, as metastable precipitates dissolve at this temperature, contributing to high strength and low deformability [280]. During warm shaping of the AW-7075-T6 aluminium alloy, it was determined that the suitable processing temperature, enhancing the plastic properties of the charge while maintaining the strength properties of the car bracket, is 240 °C [281]. The authors of [282] fabricated a U-shaped element from AW-7075-T6 sheet metal at elevated temperatures and identified optimal process parameters. Forming sheets at 100 °C and 150 °C resulted in high-strength products (above 540 MPa), but significant springback led to pronounced shape geometry deviations beyond permissible limits. Employing an alternative heating method with sheet temperatures of 200 °C and 240 °C enabled the production of a motor vehicle B-pillar component that satisfied both geometric and strength requirements.

Research by Shin et al. [283] aimed to identify an aluminium alloy with excellent mechanical properties and favourable castability for near-net-shape casting of automotive structural components. To achieve this, the investigation primarily focused on studying the newly developed Cu-free medium Al-6Zn-(1.0–2.3) Mg-0.1Zr-(0–0.2)Ti alloy. The objective of this research was to create an aluminium alloy that demonstrates superior mechanical characteristics and is also easy to cast. This study focused on investigating the properties of the Cu-free medium Mg 7xxx Al-6Zn-(1.0–2.3) Mg-0.1Zr-(0–0.2)Ti alloys. According to the analysis by Svendsen [284], it is projected that the quantity of aluminium used in vehicles will increase to 514 pounds per car by 2026, representing a 12% rise compared to the levels seen in 2020. The use of aluminium sheets in various applications, such as closures (e.g., hoods, doors), body-in-white, and chassis, has emerged as a significant development area. Additionally, the rise in electric cars has contributed to this upward trend.

The uses of 7xxx-series aluminium alloys in the automotive industry are presented in Table 10.

### 3.10. 8xxx-Series Aluminium Alloys

The 8xxx series of aluminium alloys is a distinct classification distinguished by prominent characteristics, particularly renowned for their exceptional conductivity. The alloys being examined are characterised by including lithium and/or iron as the main alloying element, specifically for use in the aviation and aerospace sectors [207,264]. The development of aluminium–lithium alloys has been undertaken to enhance mechanical strength, decrease density, and achieve outstanding electrical conductivity. While the use of the 8xxx series has been seen in some industries, its level of acceptance is minimal when compared to other series of aluminium. According to the literature review, the 8xxx series of aluminium alloys are commonly used in the aerospace industry (i.e., AW-8090) and defence applications where high-performance properties are required. This group of aluminium alloy sheets is also used as packaging materials. The applicability of 8xxx-series Al-based alloys in the automotive industry is considerably limited. The authors found no applications of 8xxx-series alloys for the production of body panels and structural members in the automotive industry.

### 3.11. 9xxx-Series Aluminium Alloys

**Figure 11:**
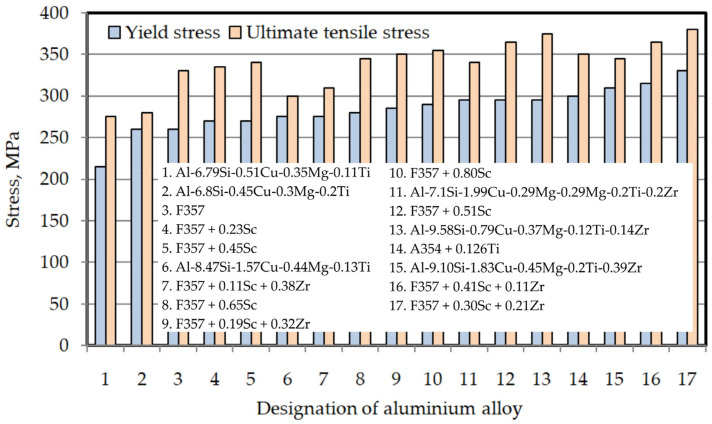
The strength of Al–Si alloys, prepared on the basis of data from [209,287,288,289,290].

The 9xxx series of aluminium alloys “Reserved for future use” [264] is renowned for its remarkable strength and superior mechanical properties, mainly attained by including scarce metals like zinc and copper. These alloys are well recognised for their exceptional mechanical qualities, rendering them well suited for aerospace and military applications that prioritise the need for robustness and lightweight materials [286]. Davies [209] finishes by offering an analysis of potential future advancements in the automobile industry regarding the development of lightweight Al–Si cast aluminium alloys. Figure 11, as shown in the work of [209] and sourced from [287,288,289,290], illustrates the mechanical properties exhibited by micro alloyed Al–Si cast aluminium alloy at the period of maximum ageing. The chemical composition of the F357 aluminium alloy is as follows (in wt.%): Si 6.5–7.5, Fe 0.10, Cu 0.20, Mn 0.10, Mg 0.40–0.7, Zn 0.10, Ti 0.04–0.20, Be 0.002, Al—balance. Xu et al. [287], Liu et al. [288], Rahimian et al. [289], and Mohamed et al. [290] investigated the influence of trace elements, including Zr, Ti, V, and Sc, on the mechanical characteristics of the Al–Si cast aluminium alloy. The study states that incorporating microalloying components into Al–Si cast aluminium alloy significantly improves its mechanical properties.

## 4. Titanium and Titanium Alloys

### 4.1. Characterisation of Titanium and Titanium Alloys

#### 4.1.1. Titanium

Titanium has been considered a prominent structural metal for more than two centuries, alongside magnesium, iron, and aluminium. The earliest discovery of Titanium may be traced back to the year 1791, and it is commonly attributed to the British mineralogist William Gregor [291]. Titanium has strength similar to certain types of steel. Titanium alloys exhibit a modulus of elasticity that is roughly 50% lower than steel and nickel alloys. The increased elasticity or flexibility of the material results in reduced bending and cycling loads in applications influenced by deflection. The main justification for the use of titanium-based goods is rooted in the remarkable corrosion resistance exhibited by titanium, together with its favourable combination of low density (about 4.5 g/cm^3^ or 0.16 lb/in.^3^) and high strength. The minimum yield strengths of various titanium grades exhibit a range of values, with certain commercial grades having a yield strength of 480 MPa (70 ksi), whereas structural titanium alloy products possess a yield strength of around 1100 MPa (160 ksi). Specialised forms such as wires and springs can surpass a yield strength of 1725 MPa (250 ksi). Moreover, some titanium alloys, specifically the low-interstitial alpha alloys, are commonly utilised in sub-zero and cryogenic environments owing to their ability to withstand the ductile-brittle transition [229]. Titanium manifests in two allotropic varieties, denoted as α and β. The α variety crystallises in the A3 hexagonal close-packed crystal structure, remaining stable up to a temperature of 882 °C. Within distinct titanium grades, the transformation temperature values may vary depending on impurity content (for instance, the stability temperature of the α variety falls within the range of 860–960 °C). On the other hand, the β variety crystallises in the cubic, spatially centred system A1 and maintains stability from 882 °C to the melting point. Due to its advantageous ratio of mechanical strength to density, referred to as specific strength, titanium is a fundamental material in the aviation industry [292]. The selection of suitable techniques for processing titanium alloys and aluminides, such as casting, wrought, or powder metallurgy (PM), is contingent upon cost and performance considerations. The high cost of wrought processes can be attributed to the material removal and slow machining, ultimately resulting in optimal material properties. Due to the ability to manufacture components in proximity to their ultimate form, castings offer a cost advantage, albeit large-scale production remains costly [293]. The mechanical properties of titanium are contingent upon its purity. With an increase in the content of admixtures (such as Fe, N, C, O, H, and Si), plasticity diminishes, while concurrently, strength parameters and hardness escalate [294]. The effects of strain hardening in titanium can be mitigated through annealing. Pure titanium’s mechanical properties can be altered through plastic forming. The yield strength of commercially pure titanium (CP) spans from approximately 170 MPa (Grade 1) to 480 MPa (Grade 4), with tensile strength ranging from 240 MPa (Grade 1) to 550 MPa (Grade 4) [294].

Grade 1 titanium stands out as the most ductile grade, renowned for its high corrosion resistance, making it particularly suitable for applications in the chemical and marine industries. Grade 2 titanium, the most popular weldable grade, finds use in construction and medical applications. Grade 3 titanium exhibits greater strength than Grade 1 and Grade 2 but is less susceptible to plastic deformation. Grade 4 Titanium is a commercially available titanium alloy with a high degree of purity. This alloy is composed of titanium as the base metal, with minor additions of aluminium (4 wt.%) and vanadium (1 wt.%). This alloy is classified within the category of alpha-beta titanium alloys.

Owing to their commendable susceptibility to plastic deformation, Grade 1 and Grade 2 titanium sheets undergo shaping through bending, drawing, deep embossing, and spinning technologies. Regrettably, titanium shaped under cold forming conditions exhibits substantial elastic deformations upon unloading.

#### 4.1.2. Titanium Alloys

**Figure 12:**
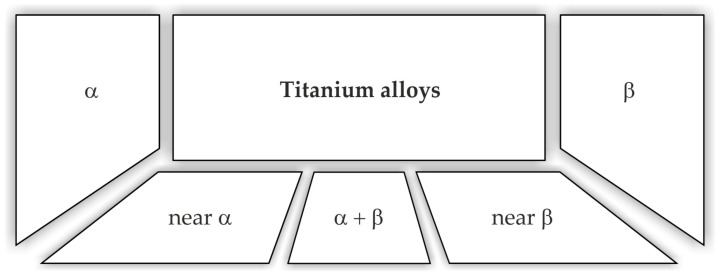
Classification of the titanium alloys.

Titanium alloys crystallise in two crystallographic systems, categorisable into single-phase α-type alloys, alloys proximate to the α-phase type, two-phase α + β alloys, β-type alloys, and alloys near the β-phase [295,296] (Figure 12). The α ↔ β transition temperature can be modified by incorporating alloying elements [297]. The α phase finds stabilisation from elements like carbon, nitrogen, and aluminium, while chromium, manganese, niobium, molybdenum, and vanadium stabilise the β phase. β-type alloys exhibit good ductility, but lower strength compared to α alloys [298]. The elastic modulus of this group of materials ranges from 105 GPa to 120 GPa. Owing to the high content of elements stabilizing the β phase [299] and retarding the ageing process, β-type alloys exhibit a low tendency toward strain hardening.

Grade 5 denotes a titanium alloy engineered to function as a versatile and extensively applicable substance. The alloy in question is classified as a highly stabilised alpha-beta alloy, with aluminium as the stabiliser for the alpha phase and vanadium as the stabiliser for the beta phase. The structural arrangement described exhibits significant durability and impressive resistance to high temperatures, particularly in temperatures reaching up to 750 °F (399 °C) [300].

Grade 6 titanium alloy consists of 5 wt.% aluminium and 2.5 wt.% tin. This alloy is commonly referred to as Ti-5Al-2.5Sn. The use of this alloy in airframes and jet engines is attributed to its favourable weldability, stability, and high strength under increased temperatures. Grades 7, 11, and 12 are technically pure titanium grades modified with a small addition of palladium. Grade 7 boasts the best corrosion resistance among all titanium alloys, commonly used in chemical equipment. Grade 11 displays excellent susceptibility to deep extrusion, akin to Grade 1, with the palladium content imparting resistance to corrosion in chemically aggressive acid environments. Grade 12, containing additions of molybdenum and nickel in addition to palladium, enhances corrosion resistance and material strength. It is used interchangeably with Grade 1 in environments requiring high corrosion resistance. Table 11 lists the titanium alloys grouped by their mechanical structure, with examples [301].

Titanium alloys are distinguished by a robust yield strength surpassing 1550 MPa for two-phase α + β and single-phase β alloys, coupled with a relatively low density of 4600 kg/m^3^. The tensile strength of titanium and its alloys spans from R_m_ = 290 MPa for pure Grade 1 titanium to approximately 1750 MPa for heat-treated β-type alloys [302]. Utilising titanium alloys enhances structural strength while concurrently reducing weight, exhibiting approximately 170% less density than high-strength steels [303]. β alloys demonstrate superior fatigue resistance compared to α alloys. The α + β alloys are crafted by introducing α (2–6%) and β (6–10%) phase stabilisers to facilitate the formation of α-Ti and β-Ti grains. In general, the microstructures of Ti-based alloys are generally described by the size and arrangement of α and β phases. Fan et al. [304] distinguished the extreme case of phase arrangement in titanium alloys.

Equiaxed microstructure resulting from globularisation, and recrystallisation provides high fatigue propagation resistance. The lamellar microstructure is the result of cooling from the β phase field and is characterised by lower ductility and lower strength compared with equiaxed microstructure [305]. The advantage of an equiaxed microstructure is better fatigue initiation resistance. The bimodal microstructure is a combination of equiaxed and lamellar microstructures and provides a well-balanced fatigue performance [306].

Components with complex shapes must be formed at elevated temperatures due to the limited deformability of titanium alloys in cold forming. Better mechanical properties of titanium alloys can be improved by either developing new alloy systems with different compositions or modifying the existing titanium alloys. Adding alloying elements in Ti-based alloys improves the key mechanical properties (e.g., Young’s modulus, formability, strength) and promotes the generation of ordered intermetallic compounds. Another way to improve the properties of titanium alloys and adapt them to specific design requirements is by introducing them into non-metallic particles and thus producing metal-matrix composites with improved performance [307].

Titanium alloys display excellent corrosion resistance to salt and seawater environments. The use of titanium is also expected in automobiles to protect against this de-icing salt. Titanium is also considered to be helpful in not only weight reduction but also size reduction [308]. However, automotive parts made of titanium alloys are generally too expensive, and the high costs make it difficult to popularise this group of materials in body panels and chassis. Typically, titanium alloys are used for responsible engine connecting rods, engine valves, valve spring seats, and exhaust systems.

The most prevalent of all titanium alloys is the α-β alloy designated Ti-6Al-4V, covering over 50% of the economy’s demand for titanium alloy sheets [309]. However, the widespread use of Ti-6Al-4V alloy sheets is constrained by its limited ductility at room temperature [310]. The Ti-6Al-4V alloy can be supersaturated, quenched, and aged to medium/high strength and is then characterised by good deformability due to the presence of the β phase. The β phase possesses a much higher diffusivity and more accessible slip planes than α alloys and alloys akin to α [309].

The formability of titanium alloys varies significantly depending on chemical composition and processing temperature. In circumstances necessitating extensive plastic deformations, titanium and its alloys are processed at elevated temperatures [311]. Titanium alloys in the supersaturated state can undergo cold forming, and the ageing of products made of titanium alloys permits the attainment of material strength within the range of 1300–1500 MPa [312].

### 4.2. Application of Titanium and Titanium Alloys

**Figure 13:**
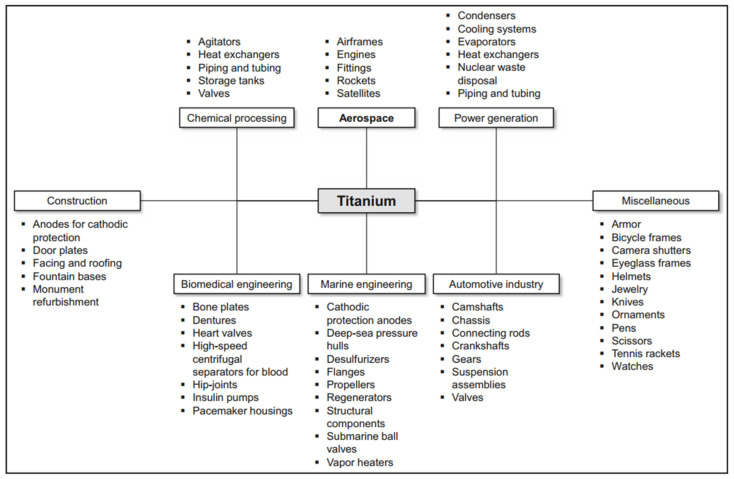
The main applications of titanium and titanium alloys (reproduced with permission from Reference [314]; copyright © 2024, Springer Nature Switzerland AG).

**Figure 14:**
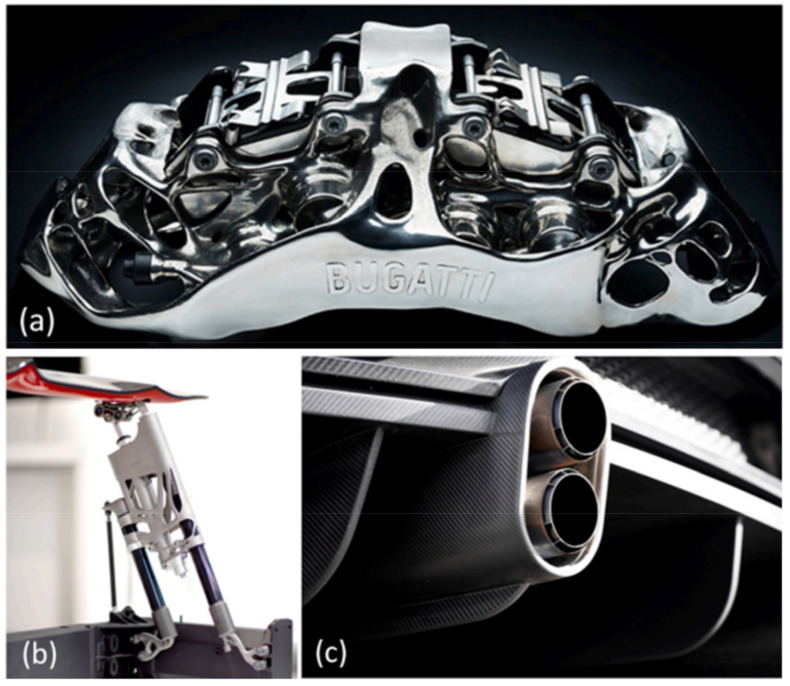
Bugatti titanium components: (**a**) eight-piston monobloc brake calliper, (**b**) active spoiler bracket, and (**c**) tailpipe trim covers (reproduced with permission from Reference [319]; copyright © 2024 The Authors. Published by Elsevier B.V.).

**Figure 15:**
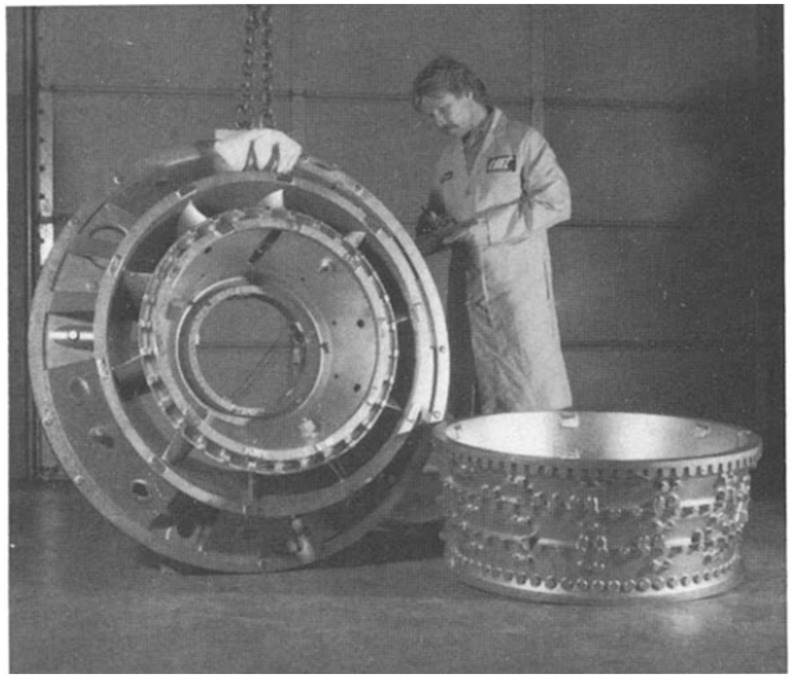
Large titanium engine component by Precision Castparts Corporation (reproduced with permission from Reference [320]; copyright © 2024 Published by Elsevier B.V.).

The varied uses of this material encompass a wide range of industries [313], including aerospace, automotive, marine, construction, chemical, and medical industries. The basic properties of titanium alloys render them highly suitable for a wide range of applications, including but not limited to springs, bellows, body implants, dental fittings, dynamic offshore risers, drill pipes, and sports equipment [301]. Figure 13 by Galanella and Malandruccolo [314] shows the main applications for titanium and titanium alloys based on [293,315]. Titanium and its alloys have been used to make many different kinds of automotive components. Based on [316,317], Table 12 shows a sample of these uses by [318], and Figure 14 shows some examples of Bugatti titanium components in the automobile sector [319]. Figure 15 shows Precision Castparts Corporation carried out the densification of a large engine component casting made of titanium [320]. Recently, there has been an observable increase in the popularity of motorbikes equipped with titanium exhaust pipes and their corresponding mufflers. The utilisation of titanium components in four-wheeled vehicles is predominantly observed in the aftermarket sector. However, the benefits associated with weight reduction and improved design have resulted in an increasing adoption of titanium in newly manufactured automobiles that are commercially accessible [321]. The incorporation of intake and exhaust valves made from titanium-based alloys into the engine of Toyota Motor Corporation’s Altezza model occurred in 1998. Both valves were manufactured using a novel and economically efficient powder metallurgy technique that had just been invented [322]. The application of titanium in exhaust systems initially emerged within the domain of motorcycles, and in recent years, there has been a substantial increase in the market for such designs. Several versions of high-performance sport bikes manufactured by Honda, Yamaha, and Kawasaki include titanium in their exhaust systems [323].

The plastic processing of titanium alloy sheets is pivotal in contemporary techniques for crafting high-quality products with intricate shapes [324]. Nevertheless, the plastic forming of titanium alloys proves challenging due to the limited formability of titanium sheets at ambient temperature. Titanium sheets tend to spring back upon unloading, and the titanium tends to adhere to the working surfaces of tools. Forming titanium alloys is integral to producing components for the automotive, aerospace, and sports equipment industries, as well as biomedical engineering. Given titanium’s relatively modest production volume and alloy products, the incremental forming method is frequently employed in unit and small-lot production. The forming of titanium alloys primarily occurs under elevated shaping temperatures, using various heating methods for the charge [325,326], including laser, hot air, electric heaters, or high-temperature-resistant hot fluids. A comprehensive discussion of strategies for incrementally shaping titanium and its alloys, encompassing machining accuracy, optimisation of tool movement trajectories, and friction conditions, can be found in the review paper [327]. The latest trends in the development of sheet processing for non-ferrous metal alloys, including titanium alloys, are expounded in [328].

**Figure 16:**
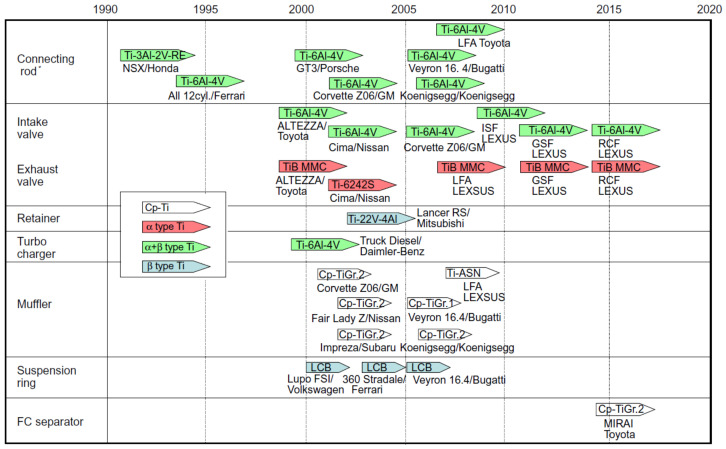
Development chronologically of titanium parts (reproduced with permission from Reference [331]; copyright © 2024 Elsevier Inc. All rights reserved).

Titanium and its alloys are used mainly for piston pins, brake calliper pistons, lug nuts, clutch discs, pressure plates, turbocharger rotors, fasteners, and engine elements [329]. However, the use of titanium and its alloys for body panels and suspension system elements is very limited (Figure 16) [330,331]. The uses of titanium and titanium alloys in the automotive industry are presented in Table 13.

## 5. Magnesium and Magnesium Alloys

### 5.1. Characterisation of Magnesium and Magnesium Alloys

Magnesium is a grey-white lightweight metal with an atomic weight of 24.305 and it crystallises in an A3 hexagonal close-packed crystal structure. Magnesium is 33% lighter than aluminium, 50% lighter than titanium, and 75% lighter than steel [338]. Magnesium is the eighth most abundant element on Earth; its share in the Earth’s crust is approximately 1.93%. The density of magnesium is 1738 kg/m^3^, and the density of magnesium alloys is in the range of 1400–1900 kg/m^3^ [339]. Magnesium deformation occurs as a result of the activity of slip and twinning mechanisms. Pure magnesium is practically not used as a construction material due to its low mechanical properties [340].

Magnesium alloys can be divided into two types including wrought magnesium alloys and cast magnesium alloys. The properties of magnesium alloys can be modified by selecting appropriate alloy additives. The main alloying components of magnesium alloys are aluminium (up to 10 wt.%), beryllium (up to 0.001 wt.%), cerium, copper, lithium, neodymium, manganese and rare earth (RE) elements [341]. Magnesium alloys also contain alloy additions such as calcium, cadmium, and nickel, but their content does not usually exceed 1 wt.%. The addition of 6 wt.% aluminium optimally improves the strength and hardness of the Mg alloy while making it easier to cast. Calcium added in small amounts increases grain refinement. Calcium in amounts below 0.3 wt.% enables welding of sheet metal without the risk of cracking [342]. The main mechanisms applied for strengthening the magnesium alloys include solution strengthening and precipitation strengthening. The decrease in the solubility of the alloying component in Mg-based alloys with decreasing temperature results in a reduction of the solution strengthening effect [340]. The effects of precipitation hardening in magnesium alloys are much smaller than in aluminium alloys.

Due to their chemical composition, several basic groups of cast Mg alloys can be distinguished, containing the main alloying elements such as: aluminium, zinc, manganese, zirconium, and rare earth metals [343]: Mg–Al and Mg–Al–Zn, Mg–Al–Mn, Mg–Al–Si, Mg–Zn–Cu, Mg–Zn–Zr, Mg–Zn–RE–Zr, Mg–Ag–RE, Mg–Y–RE, Mg–Th, Mg–Sc, Mg–Li.

Baseline wrought magnesium alloys contain Al (up to 8 wt.%) and the addition of Mn (up to 2 wt.%), Zn (usually up to 1.5 wt.%), Si (about 0.1 wt.%) and trace additions of copper, iron and nickel. There are three groups of wrought Mg-based alloys [343,344]:

- alloys with the addition of aluminium, zinc and manganese: Mg–Mn, Mg–Al–Zn, Mg–Zn– (Mn, Cu),
- alloys containing mainly zinc, yttrium, zirconium, thorium and RE elements: Mg–Zn–Zr, Mg–Zn–RE, Mg–Y–RE–Zr, Mg–Th,
- alloys containing lithium, for example Mg–Li–Al.

The number of magnesium grades produced in the form of sheet metals is limited to typical grades AZ31B, AZ61, HK31, HM21, and ZM21, usually available in annealed (O) or partially hardened (H24) temper [345]. One of the problems that limit the formability of magnesium alloy sheets, apart from crystallisation in the A3 lattice, is the anisotropy of mechanical properties. Nerite et al. [346] distinguished three factors that increase the plasticity of magnesium alloys, i.e., increased forming temperature, grain refinement, and the presence of elements with a low melting point, e.g., lithium or zinc. Hot forming of magnesium alloys is usually carried out in the temperature range of 200–350 °C [345].

### 5.2. Application of Magnesium Alloys in Car Body Components

To reduce fuel consumption, general efforts have been made to reduce the weight of automotive structures through the increased use of non-ferrous metal alloys, including magnesium alloys. Magnesium alloys have great potential as structural materials in the automotive industry due to their low density, high specific strength, high dimensional stability, high thermal conductivity, and high vibration damping properties [347]. In magnesium alloys with high strength [348], the damping capacity is generally 60 times higher than that of steel and 15 times higher than that of aluminium alloys [349]. The automotive industry is characterised by the highest share in the consumption of magnesium alloys (70%) [350].

Magnesium alloy was first used in the automotive industry in 1918, when Mg-based engine pistons in the Indy 500 were introduced [351]. The use of magnesium and its alloys in the automotive industry has been the subject of research almost since the beginning of the United States Automotive Materials Partnership (USAMP), established in 1993 by the consortium of Fiat-Chrysler, Ford and General Motors. The first USAMP project on magnesium alloy sheets was ‘Magnesium Front End Research and Development’, initiated in 2007, in which magnesium alloy AZ31B (Mg-3Al-1Zn) was tested for formability and crashworthiness [352]. Another USAMP project [353] found that the room temperature formability of typical magnesium alloys, such as AZ31B, did not meet automotive requirements in terms of acceptable costs and performance required for high-volume production. In 2016, USAMP was awarded a project by the U.S. Department of Energy, Low-Cost Magnesium Sheet Component Development and Demonstration Project, to investigate the formability of Mg-based sheet metals [354]. Luo et al. [354] presented the results of another project implemented by the University of Ohio as part of USAMP, in which the ZEK100 (Mg-1.2Zn-0.17Nd-0.35Zr), E-Form (POSCO) and E-Form Plus (POSCO) alloys brought optimistic formability results. The newly developed Mg-1.0Zn-1.0Al-0.5Ca-0.4Mn-0.2Ce alloy (USAMP Alloy 2 Plus) offers good plasticity (tensile elongation 31%) and formability at room temperature determined by the Erichsen method IE = 7.8 mm [354].

Currently, AM60B, AZ91D, and AM50 are the most dominant alloys in exterior and interior trim systems, body and chassis systems [355,356]. The uses of magnesium alloys in the automotive industry are presented in Table 14.

## 6. Future Developments Directions

The automotive industry is dynamic, so market demands, regulatory changes, and technological advances will likely influence material developments. Manufacturers must adapt to these changes to stay competitive and address global sustainability and safety issues. Furthermore, advanced driver-assistance systems (ADAS) and autonomous vehicles benefit from materials that can directly host sensors or electronics. It is developing supplementary automotive metal materials like composite materials and hybrid structures using metals and carbon fibre-reinforced polymers to balance strength, weight, and cost [391,392,393]. Use smart materials like shape memory alloys (SMAs) or self-healing materials to improve durability, safety, and performance. Over the past few decades, SMAs have been increasingly utilised in various industrial sectors, including automotive [394]. SMAs are smart materials that can remember their form when thermomechanical or magnetic gradients are stimulated. SMAs’ unique and outstanding features have garnered attention in various commercial applications [395,396]. Additive manufacturing has significantly enhanced several applications, particularly in the car industry. Various techniques have been developed to improve and expand its use throughout the manufacturing process for producing reliable automobile parts [397]. Advanced manufacturing (3D printing) allows complex geometries, reduced waste, and customised components [398]. Approximately 30% of the current worldwide market share of 3D printing technology is dedicated to serving the automotive industry, among other sectors [399]. The article in [400] provides a comprehensive analysis of the advantages and disadvantages of the 3D printing methods employed in the automotive industry. However, exploring new materials should focus on developing cost-effective alternatives to traditional materials to make the technology economically viable for mass production. Automotive manufacturers (including Volkswagen) strive for zero emissions not only in the context of the cars they offer, but also in the production process itself and the supply chain (‘go-to-zero’ strategy). The steel sheet metal industry has significantly reduced environmental pollution. Today, its production uses about 60% less energy than in the 1960s. In 2021, the Volvo Group developed a production technology for the so-called “green” steel. This process uses pure hydrogen, produced from electricity from renewable sources, instead of fossil fuels.

Intensive work is still underway to develop another generation of AHSS steels with even greater strength and satisfactory formability. New cold stamping technologies (instead of roll forming) open the possibility of designing components with complex shapes from martensitic steel with a strength of 1500 MPa or 1700 MPa. Cold-formed martensitic steels replace some grades of press hardened and DP steels.

Tailored blanks, which are a combination of different types of materials, enable the optimisation of crash performance and strength with minimal material consumption. These types of materials make it possible to eliminate or limit the joining of many components together, reducing production time, component manufacturing time, and assembly time. In this context, an interesting new technique is the replacement of stamped and extruded sheets with a single component manufactured using ‘mega-giga’ casting technology.

Until recently, the need to transform and optimise the forming process was a barrier to the use of completely new materials in the automotive industry. However, as the transformation begins in line with the e-mobility concept and the construction of new cars, there is the potential to introduce new lightweight materials for the production of body-in-white structures.

Lightweight metals such as aluminium and titanium alloys are becoming increasingly important in the automotive industry because they help reduce vehicle weight and lower fuel consumption or electric energy usage by battery electric vehicles. Every year, there is an increasing share of mainly aluminium alloys in the total weight of vehicles. Non-ferrous metals are also easier to recycle than conventional steels. The use of magnesium alloys, which have even greater potential to reduce the weight of vehicles than aluminium alloys, is limited due to low formability and low melting point, making safe machining difficult.

Although they offer high strength and excellent stiffness, composite materials allow for much more efficient weight reduction. However, due to the difficulties in recycling, the use of these materials is still limited to luxury vehicles. The development of mass technology for recycling epoxy composites may reverse the proportions of the use of metallic and composite materials in the automotive industry in the future.

## 7. Conclusions

With the exception of some cars, it appears that steel will remain a cheap and widely used construction material. Much will depend on the price of individual materials in the future, the development of material production processes, and market requirements in terms of reducing the weight of the car in order to reduce fuel consumption. Owing to the ease of recycling metallic materials, the widespread replacement of these materials with composite equivalents seems to be in the distant future.

HS steels in the automotive industry are among the most important materials used in the production of cars and other vehicles. This material is characterised by high durability, stress resistance, corrosion resistance, and lower weight compared to conventional steels. The factors that determine the use of steel as a construction material for the automotive industry are low production costs compared to other materials and the possibility of easy recycling. In general, the process of reducing the weight of vehicles can be carried out by reducing the thickness of steel elements. In this field, the group of stainless steels constitutes a concretion for low-alloy steels.

The areas requiring development in the near future include the development of technologies for the deep drawing of materials covered with metallic coatings, varnishes, and foils, as well as methods for producing deep-drawing steel sheets with the desired anisotropic features.

Methods of improving the strength of HS and AHSS steels, under the condition of not changing the composition, mainly include combing the grain refinement strengthening of the precipitates and the precipitation strengthening, using cyclic pre-quenching to prepare a uniform lamellar microstructure.

Car structural elements made of aluminium alloys are not only highly resistant to weather conditions, but also have high durability, ensuring safety in the event of a collision. Side beams in door systems, side members, and energy absorbers are increasingly common aluminium-based components. Aluminium alloys are also increasingly used in the body panels of cars, buses, and commercial trucks as an alternative to stainless steel.

Despite the advantages of titanium sheets, such as high specific strengths, low density, and good corrosion resistance, their use in the production of car bodies is limited to unit production and luxury vehicles manufactured in small series. The high price of titanium sheets and the difficulty in forming titanium alloy sheets are the main limitations in increasing the share of these sheets in sheet metal stamping for the automotive industry. However, as demand for more fuel efficient and environmentally friendly cars increases, affordability will become less of an issue, increasing the automotive industry’s interest in titanium sheets.

## Figures and Tables

**Table 1:** Selected properties of cold-rolled flat products from low-carbon steel, prepared on the basis of PN-EN 10130 [62] standard.

Grade	Yield Stress R_p0.2_ (max.), MPa	Ultimate Tensile Strength R_m_, MPa	Elongation A_50_ (min.), %
DC01	280	270–410	28
DC03	240	270–370	34
DC04	210	270–350	38
DC05	180	270–330	40
DC06	180	270–350	38
DC07	150	250–310	44

**Table 2:** Main applications of the steel sheets used for body panels and structural members; (*) grade not specified.

Steel Group/Grade	Components	Reference
PHS	press hardened body parts	[147]
PHS	press hardened front and/or rear bumper beam	[148]
PHS	A- and B-pillars	[149]
PHS	transmission tunnel and the firewall (Volkswagen Passat)	[150]
PHS	B-pillar (Audi A8)	[151]
PHS	body panels (Dongfeng Voyah iFree)	[152]
PHS	press hardened door beams (Jaguar XJ40)	[153]
PHS	door beams (VW Polo)	[154]
PHS	press hardened bumper beam (Renault Safrane)	[148]
PHS	press hardened bumper beams (Volvo S80)	[155]
PHS	A-pillar (BMW series 3)	[156]
PHS	3rd row seating support (Volvo V70)	[157]
PHS	front bumper beam, and the right/left A-pillars (Citroën C5)	[149]
PHS	2nd row seat frame (Volvo XC90)	[158]
PHS	“form fixture hardened” front and rear bumpers (Ford Mustang 5th generation)	[157]
PHS	tailor-rolled and press hardened components (Dodge Caliper, BMW X5)	[159,160]
PHS	tailor welded B-pillar (Audi A4)	[161]
PHS	B-pillar reinforcement, A-pillar reinforcement, rear rail, tunnel reinforcement, upper B-pillar reinforcement (BMW 7 series 5th generation)	[162]
PHS	modular transverse platform (Volkswagen Golf, Audi A3)	[163]
PHS	swing doors in the front and two sliding rear doors (Ford B-Max)	[164]
PHS	A- and B-pillars, hinge pillar, and front portion of the rocker reinforcement (Honda Acura MDX, 3rd generation)	[165]
PHS	A-pillars (Honda Acura NSX)	[166]
PHS	a tailor rolled tube (Ford Focus fourth gen., Jeep Wrangler 4th generation)	[167]
PHS	A-pillar reinforcements (BMW E46 Cabrio, Citroën C5)	[156,168]
PHS	B-pillar reinforcements and roof rail (Peugeot 307)	[169]
PH1500	B-pillar reinforcement (Chrysler Pacifica)	[170]
PHS1800	bumper beam reinforcements (Mazda CX-5)	[171]
PHS2000	hot stamped door ring (third generation Haval H6)	[172]
Boron steel (*)	B roof bow, rear seat frame (Volvo XC90)	[173]
MBW 1900 (PHS)	seat crossbeams (Volkswagen ID.3)	[174]
22MnB5	hot stamped B-pillars (Audi A5 Sportback, Volkswagen Tiguan)	[175]
PQS450	front side members, B-pillar reinforcements, rear side members (Volvo XC90)	[158]
PQS450	rear side member (Fiat Tipo)	[163]
PQS450	laser-welded tailored rear side member (Fiat 500X)	[176]
PQS450	B-Pillar (Jaguar I-Pace)	[176]
PQS550	front door ring, B-pillar (Chrysler Pacifica, Chrysler RAM)	[177,178]
PQS550	tailored B-pillar (Renault Scenic 3)	[179]
PQS550 + PHS1500	patchwork B-pillar (Mercedes C-class)	[180]
HSLA	the energy absorbing components	[181]
Rephos (a phosphorus alloy high-strength steel)	A-pillar upper, W-screen member (Volvo XC90)	[173]
TRIP steels	front longitudinal beams, A-pillar and B-pillar reinforcements	[96]
TRIP 350/600	rail reinforcements, frame rails	[182]
TRIP 400/700	crash box, side rails	[182]
TRIP 450/800	roof rails, dash panels	[182]
TRIP 600/980	engine cradle, roof rail, front and rear rails, B-pillar upper, seat frame	[182]
TRIP780	bumper cross member, B-pillar reinforcement	[183]
MS steels (*)	front and rear bumpers, roof cross members, door beams	[123]
MS 950/1200	bumper beams, cross members, side intrusion beams	[184]
MS 1150/1400	bumper reinforcements, side intrusion beams	[184]
MS 1250/1500	bumper reinforcements, side intrusion beams, bumper beams	[184]
CR1200Y1470T-MSteel	centre roof reinforcement (Lexus NX)	[185]
DP steels	seat guides, child seats, windshield pillars	[186]
DP steels	bumper reinforcements, car body panels	[86,90]
DP90	rocker, B-pillar (Volvo XC90)	[175]
DP 300/500	floor panel, door outer, roof outer	[187]
DP 350/600	body side outer, fender, floor panel	[187]
DP 500/800	rear rails, body side inner	[187]
DP 600/980	floor panels, B-pillar, front sub-frame	[187]
DP 700/1000	roof rails	[187]
DP 800/1180	B-pillar upper	[187]
CP steels	tunnel stiffeners, pillar reinforcements, bumper beams, side beams, frame rails, rocker panels	[68,188]
FB steels	engine sub-frames, upper and lower control arms, seat cross members, longitudinal beams	[189]
IF steels	complex stampings, inner wheel arches, boot lid reinforcements	[79]
BH steels	doors, boot lids	[81]
TBF980	structural reinforcements (Infiniti QX50)	[190]
TBF 1180—TRIP-assisted bainitic ferrite steel	A- and B-pillar reinforcements, side reinforcement, roof rail (Infiniti Q50)	[191]
TBF 1180	A-pillar inner and reinforcements (Nissan Murano)	[192]
TBF 1180	A- and B-pillar reinforcements (Nissan Maxima)	[193]
QP980	front and rear floor assemblies (Ford Bronco)	[135]
QP980	A-pillar inner lower, A-pillar inner upper, hinge pillar inner, kick down lower (General Motors vehicles)	[194]
TWIP	energy-absorbing elements and structurally responsible components with complex shapes	[99]
TWIP	A-pillar, B-pillar, crash box, bumper beam, dash lower reinforcement, tunnel, floor cross-members, door hinge reinforcement, wheelhouse, front side member, rear side member, door impact beam, shock absorber housings (i.e., General Motors, Daewoo, Ssangyong, Renault)	[195,196]
TWIP1000	bumper beam (Fiat Nuova Panda)	[196]
TWIP 450/950	bumper reinforcements (Jeep Renegade BU/520)	[197]
TWIP980	sill side outer, A-pillar lower (Renault EOLAB concept)	[198]
stainless steel (*)	buses’ frames, side members, bumper beams, self-supporting body, panels, outer covering body, body panels, structural frameworks of buses and coaches	[199,200,201]
stainless steel (*)	energy absorbing components (Volvo)	[199]
stainless steel (*)	auto body components (Audi A6), bumper system and collision boxes (Saab), frame (Hyundai Mobis), fuel tanks (Volkswagen Beetle)	[199]
AISI 304, AISI 304L	fuel tanks	[202]
AISI 304, AISI 304L, AISI 321, AISI 3167, AISI 316L, ASTM S30415	housings for turbochargers and catalytic converters	[202]
AISI 304, AISI 304L, ASTM S41050	chassis for buses and trucks, structural components	[202]
AISI 304, AISI 304L, AISI 430, AISI 434, AISI 436	door scuff plates, bumpers, headlight bezels	[202]
AISI 304L	automobile frames, A-pillar structural tubes,	[203]
AISI 304	whole frame (Pininfarina Nido)	[199]
H400 stainless steel	rear and front side members, lower rear axle wishbone (Porsche Carrera GT)	[199]
Fe-15Cr-10Mn-0.35Ni-1.6Cu-0.12 stainless steel	bumper (Ashok Leyland)	[204]
AISI 304	fuel tank (Fiat Barchetta)	[204]
Nitronic 30 (15Cr-1.5Ni-8Mn-0.18N) stainless steel	urban bus frames (Autocinetics Inc.)	[205]

**Table 3:** Non-heat-treatable wrought aluminium alloys.

Alloy Series	Alloy Additions
1xxx	Al (99% pure)
3xxx	Al-Mn
5xxx	Al-Mg

**Table 4:** The classification and basic properties of aluminium alloys for plastic forming, prepared on the basis of [211].

Alloy Series	Type of Alloy	Ultimate Tensile Strength, MPa	Basic Properties
1xxx	Al (impurity content < 1%)	70–150	good plasticity in cold and elevated temperature forming, low strength, good corrosion resistance, high electrical and thermal conductivity
2xxx	Al–Cu–Mg Al–Cu–Mg–Si	170–530	low corrosion resistance
3xxx	Al–Mn–Mg	140–280	good plasticity but low strength, good weldability, and corrosion resistance
4xxx	Al–Si	100–360	high strength and corrosion resistance, good casting properties
5xxx	Al–Mg Al–Mg–Mn	140–350	good saltwater corrosion resistance, good weldability and analysability
6xxx	Al–Mg–Si	160–370	high corrosion resistance, good formability, very good anodising ability
7xxx	Al–Zn–Mg Al–Zn–Mg–Cu	360–610	the highest strength of all aluminium alloys, low and medium corrosion resistance
8xxx	various alloying elements, Al–Li, Al–Fe, Al–Li–Cu–Mg	260–580	properties depending on the chemical composition
9xxx	zinc and copper	Not specified	remarkable strength and superior mechanical properties depending on the specific alloy composition

**Table 5:** Main applications of the 2xxx-series aluminium alloys in car body structures.

Aluminium Alloy Grade	Components	Reference
EN AW-2008, EN AW-2010	outer and inner body panels	[225]
EN AW-2013	seat shells, load floors, outer and inner body panels	[226]
EN AW-2016	external body panels	[227]
EN AW-2024	wheel spokes, structural components	[228]

**Table 6:** Main applications of the 3xxx-series aluminium alloys in car body structures.

Aluminium Alloy Grade	Components	Reference
EN AW-3003	trailer and truck panels, awning slats, pressure vessels, piping	[231]
EN AW-3004	truck trailer sheet, horse trailers, interior panels and components	[212]
EN AW-3105	floor components	[232]

**Table 7:** Main applications of the 4xxx-series aluminium alloys in car body structures.

Aluminium Alloy Grade	Components	Reference
EN AC-43500	suspension strut dome (Audi A7, BMW 5 Gran Turismo)	[235]
A356 (AlSi7Mg0.3)	suspension–cradle interface (Chevrolet Corvette Stingray C7)	[235]
C448 (AlSi9Mg)	hinge pillars, brackets, B-pillar reinforcements, shock towers	[236]
AlSi10MgMn(Sr)	front longitudinal member (Audi A8), engine torsion support	[237,238]
(AlSi10MgMnFe)	front sections, shock tower, engine cradle	[239,240,241,242]
Aural^®^-2 (AlSi10MgMnFe)	one-piece B-pillar, assembled structural elements (Audi A2)	[235]
Castasil^®^-37 (AlSi9MnMoZr)	rear longitudinal beam (Audi A8 D4) components of retractable roofs	[235,243]
Castasil^®^-37 (AlSi9MnMoZr)	shock tower, front sections, engine cradle	[239,240,241,242]
Silafont^®^-36 (EN AC-43500)	engine bracket (BMW N52), front end carrier (BMW 3 series), front crash management system (Audi A2)	[237,244]
Silafont^®^-36 (EN AC-43500)	shock tower, engine cradle, front sections, space-frame members, suspensions, B-pillar reinforcements, shock towers, brackets, hinge pillars	[236,240,245]

**Table 8:** Main applications of the 5xxx-series aluminium alloys in car body structures.

Aluminium Alloy Grade	Components	Reference
AF350 (AlMg1/AlMg5.7/AlMg1)	shell doors (BMW 5 and 7) series	[249]
C446 (AlMg3Mn)	B-pillar reinforcements, shock towers, brackets, hinge pillars	[236]
EN AC-51500	suspension strut dome (BMW 5 series, Porsche Panamera)	[237]
EN AC-AlMg5Si2Mn	inner door frames (Mercedes Benz S-class)	[249]
EN AW-5022	pillars, floors, roofs, doors, oil pans, rear fenders, bonnets	[250]
EN AW-5042, EN AW-5182	weight-reduced front end (BMW E60)	[235]
EN AW-5051A-O, EN AW-5182-O	inner panels	[251]
EN AW-5052	floor components, truck trailers	[231,232]
EN AW-5052	bumpers, body panels, interior panels	[226]
EN AW-5052, EN AW-5182, EN AW-5454	internal body parts	[227]
EN AW-5052, EN AW-5083	car doors, automobile plates	[252]
EN AW-5083, EN AW-5456	complex automotive components	[212]
EN AW-5083	boot lid (Cadillac STS)	[250]
EN AW-5083	lashing rails	[250]
EN AW-5110A	reflective panels	[250]
EN AW-5154	underbody components, drivetrain components, suspension components	[250]
EN AW-5182	dust covers, seat frames, air cleaner cases, inner panels	[212,235,250]
EN AW-5182	rear fenders, car hoods, car front, and car doors	[252]
EN AW-5182	reinforcement members, inner body panels	[226]
EN AW-5182	structural panels (Audi A8 D2)	[235]
EN AW-5182	inner panels (Saab 9-3, Renault Vel Satis, BMW 3 series)	[249]
EN AW-5182	hood inner (Jaguar XE)	[253]
EN AW-5182	one-piece inner panel (Jaguar XJ353)	[235]
EN AW-5182-O	inner panel (Chevrolet GMT 830)	[249]
EN AW-5182-O	roof structure (Renault Avantime)	[243]
EN AW-5182, EN AW-5457	internal structural parts	[254]
EN AW-5754	structural sheet applications	[212]
EN AW-5454	suspension components	[250]
EN AW-5454, EN AW-5182	structural panels (Rolls Royce Phantom)	[235]
EN AW-5454	engine brackets and mounts	[226]
EN AW-5456	armour plates	[226]
EN AW-5454-O, EN AW-5754-O	body structures (sheets)	[251]
EN AW-5754	automobile plates, structural sheets, load floors, inner body panels	[226,235,252]
EN AW-5754	extrusions (Lotus Evora)	[235]
EN AW-5754	instrument panel support (Volkswagen Polo, Skoda Fabia, Seat Ibiza)	[237]
EN AW-5754	cockpit carrier (Audi A6, Audi A7)	[235]
EN AW-5745	B-pillar assembly, floor panels (Chevrolet Corvette Z06)	[235]
EN AW-5754	door inner, hood inner, B-pillar, rocker, brackets, structural reinforcements	[236]
EN AW-5754, EN AW-5182	structural sheets, inner panels (BMW Z8)	[235]
Formall^®^-545 (EN AW-5083)	inner front door (Maybach)	[249]
Magsimal^®^-59 (AlMg5Si2Mn)	doors (Range Rover L322)	[249]
Magsimal^®^-59 (AlMg5Si2Mn)	components of retractable roofs	[243]
Magsimal^®^-59 (AlMg5Si2Mn)	door frames (Rolls Royce Phantom)	[235]

**Table 9:** Main applications of the 6xxx-series aluminium alloys in car body structures.

Aluminium Alloy Grade	Components	Reference
A356 (EN AC-42000)	B-pillar reinforcements, lift gate outer, body side, shock towers	[236]
Advanz^TM^ C300-T61	long members (Jaguar XE)	[253]
Advanz^TM^ E170	fender, hood outer (Jaguar XE); doors outer (Ford F150); roof, bodyside (GM CT6)	[253]
Advanz^TM^ S600	roof, strength parts inner (Jaguar XE), fender, roof (Ford F150)	[253]
Alcoa C611 Mercalloy^®^ 367	front sections, shock tower, engine cradle	[239,240,241,242]
Anticorodal^®^-120 (EN AW-6016-PX)	outer body panels (Audi A2, Audi A8)	[235]
Anticorodal^®^-121 (EN AW-6061)	outer panel (Audi A2)	[249]
Anticorodal^®^-170 (EN AW-6014)	one piece body side (Range Rover)	[235]
Anticorodal^®^-170 (EN AW-6014), Anticorodal^®^-600 (EN AW-6451-T4)	body shell (Ferrari 548)	[235]
Anticorodal^®^-300 (EN-AW 6014-T61)	structural body applications with high crash performance (Mercedes SL R231)	[237]
Anticorodal^®^-300 (EN-AW 6014-T61)	tunnel (Mercedes SL R231), front longitudinal beam (Land Rover L405)	[237]
Anticorodal^®^-300 (EN-AW 6014-T61)	long crash members or bumper beams (Land Rover)	[269]
Anticorodal^®^-600 PX (EN AW-6181A)	exterior body panels side (Range Rover)	[235]
Anticorodal^®^-600 PX (EN AW-6181A)	roof (Range Rover Evoque)	[243]
Anticorodal^®^-600 (EN AW-6451-T4)	inner and outer panels (Range Rover)	[269]
Ecodal^®^-608 (EN AW-6181A)	inner and structural panels (Audi A2, Audi A8), cover sheet for the tunnel (Audi R8), reinforcements, inner panels (Audi A2)	[235,249]
EN AW-6005	seat frame components (extruded)	[226]
EN AW-6005C	seat frames, space frames, bumpers, shock absorbers, side sills	[250]
EN AW-6008, EN AW-6060	front longitudinal beam (BMW E60)	[235]
EN AW-6009	inner panels (Audi A8)	[235]
EN AW-6009	bumper face bars, load floors, inner and outer body panels, bumper reinforcements	[226]
EN AW-6009, EN AW-6010, EN AW-6016	inner and outer body panels	[226]
EN AW-6009, EN AW-6010, EN AW-6016	outer and inner panels	[270]
EN AW-6009, EN AW-6016, EN AW-6061, EN AW-6111	outer panels, frames	[227]
EN AW-6013	suspension arms	[250]
EN AW-6014	front longitudinal member (Jaguar XK & F)	[237]
EN AW-6014	crash-sensitive components (Range Rover), hydroformed lateral roof frame (Audi A2)	[235]
EN AW-6016	body-in-white applications (Audi A8), outer panels (BMW Z8, Rolls Royce Phantom)	[235,268]
EN AW-6016	outer panels (Lamborghini Gallardo)	[235]
EN AW-6016	external structural parts	[254]
EN AW-6016	outer panel (Saab 9-3, Renault Vel Satis, BMW 3 series, Mercedes Benz S class, Mercedes Benz SL, BMW Z8)	[249]
EN AW-6016A-T4	inner panels	[251]
EN AW-6016-T4, EN AW-6016-T4P	outer panels	[251]
EN AW-6022-T6, EN AW-6082-T6, EN AW-6181A-T6	space frame (Ferrari 548)	[235]
EN AW-6060	extrusions (Lotus Evora), space frame (Audi A8 D2), extruded structural components (Audi A8 D3, Lamborghini Gallardo)	[235]
EN AW-6060	instrument panel support (Mercedes-Benz A class)	[237]
EN AW-6060	front bumper (Opel Corsa)	[244]
EN AW-6060	sunroof channels (BMW 7 series), top rear end, transverse and cant rail assembly (Ford Think)	[243]
EN AW-6060, EN AW-6063, EN AW-6082	frame structure (BMW Z8)	[235]
EN AW-6060, EN AW-6063, EN AW-6082 (T5 or T7)	extruded components (Rolls Royce Phantom)	[235]
EN AW-6060-T51, EN AW-6106-T51	roof structure (Renault Avantime)	[243]
EN AW-6061	car steering knuckles	[212]
EN AW-6061	door outers, roof panels, body side, hood outers	[236]
EN AW-6061	brackets, suspension parts, bumper reinforcements	[226]
EN AW-6061	cross members, links, arms	[250]
EN AW-6061-T4, EN AW-6061-T6, EN AW-6063-T4	engine cradle (Chevrolet Monte Carlo)	[237]
EN AW-6061, EN AW-6063	rear frame (Corvette ZR-1/LT-1)	[237]
EN AW-6061-T6	panel applications	[271]
EN AW-6061-T6	extruded seatback beam (Chevrolet Corvette Z06)	[235]
EN AW-6063	seat frames, roof railings	[250]
EN AW-6063	body components	[226]
EN AW-6063	bumpers, hinge pillars, A-pillars, roof bows	[236]
EN AW-6063	bumper reinforcements, the roof reinforcement bar, A-pillar (Chevrolet Corvette Z06)	[235]
EN AW-6069-T7	frame rail (Chevrolet Corvette Z06)	[235]
EN AW-6070	extruded structural components	[216]
EN AW-6082	structural elements (extruded) (BMW 6) series, Rolls-Royce Phantom, Jaguar XJ, Range Rover, Jaguar XK	[272]
EN AW-6082-T6	rear bumper beam (Citroen C4 Picasso)	[244]
EN AW-6082-T6	post/cant rail aluminium extrusion assembly, door reinforcements (Jaguar XJ350)	[235]
EN AW-6106	extruded tunnel member (Porsche 9 × 1), upper A post panel (Audi R8)	[237]
EN AW-6111	closure panels (Jaguar), outer skin (Jaguar XJ353)	[212,235]
EN AW-6111	outer skins	[235]
EN AW-6111	body panels	[226]
EN AW-6111-T4	outer panels	[251]
EN AW-6111-T4P	reinforcements (Chevrolet GMT 830)	[249]
EN AW-6111-T4PD	outer panel (Chevrolet GMT 830)	[249]
EN AW-6181	inner panels (Lamborghini Gallardo)	[235]
EN AW-6181A	Inner panels	[251]
EN AW-6181A, EN AW-6022	outer panels	[212]
Novelis Advanz e600 (EN AW-6451-T6)	external panels, skin and structural applications	[212]
Novelis Fusion^TM^ AS250	floor structure (Audi A8)	[237]

**Table 10:** Main applications of the 7xxx series aluminium alloys in car body structures.

Aluminium Alloy Grade	Components	Reference
EN AW-7003	door impact beams, frames, seat sliders, bumper reinforcement	[250]
EN AW-7003	folding crash components (BMW E38), bumper systems (Renault Megane)	[244]
EN AW-7006, EN AW-7004	bumper reinforcements, seat tracks	[226]
EN AW-7020	bumper beams, complex automotive components	[212,254]
EN AW-7021	bumper reinforcements, brackets, bumper face bars	[226]
EN AW-7033	forged suspension arms	[226]
EN AW-7046	impact beams, motorbike frames, bumper reinforcement	[250]
EN AW-7071	car bumper brackets, bumper enhanced supports	[285]
EN AW-7075	links, seatbelt hinges, bars	[212,250]
EN AW-7108	rear seats for roll-over protection (Porsche 911, 966)	[244]
EN AW-7108	bumper beams (BMW E38, Volkswagen Passat B5, Audi TT), rear bumper (Opel Corsa)	[244]
EN AW-7108-T6	front beam (Jaguar XJ350)	[235]
EN AW-7204	cross members, steering components	[250]

**Table 11:** Titanium alloys grouped by their mechanical structure, with examples.

**Alloy**	**Example**
Alpha (α) alloys	Commercially pure Ti—ASTM Grades 1, 2, 3, and 4 Ti/Pd alloys—ASTM Grades 7 and 11
Alpha (α) + compound	Ti-2.5%Cu—IMI 230
Near Alpha alloys	Ti-8%Al-1%Mo-1%V Ti-6%Al-5%Zr-0.5%Mo-0.2%Si—IMI 685 Ti-6%Al-2%Sn-4%Zr-2%Mo-0.08%Si Ti-5.5%Al-3.5%Sn-3%Zr-1%Nb-0.3%Mo-0.3%Si—IMI 829 Ti-5.8%Al-4%Sn-3.5%Zr-0.7%Nb-0.5%Mo-0.3%Si—IMI 834 Ti-6%Al-3%Sn-4%Zr-0.5%Mo-0.5%Si—Ti 1100
Alpha-Beta (α—β) alloys	Ti-6%Al-4%V Ti-4%Al-4%Mo-2%Sn-0.5%Si Ti-4%Al-4%Mo-4%Sn-0.5%Si—IMI 551 Ti-6%Al-6%V-2%Sn Ti-6%Al-2%Sn-4%Zr-%Mo
Metastable Beta (β) alloys	Ti-3%Al-8%V-6%Cr-4%Zr-4%Mo—Beta C Ti-15%Mo-3%Nb-3%Al-0.2%Si—Timetal 21 S Ti-15%V-3%Cr-3%Sn-3%Al

**Table 12:** Some titanium products and how they are used in the automotive industry.

Systems and Parts Materials	
Frame structures	
Suspension springs	Ti-6.8Mo-4.5Fe-1.5Al
	Ti-6Al-4V
Armor	Ti-6Al-4V
Body	CP-Ti (Grade 4)
	Ti-6Al-4V
Engines	
Outlet valves	outlet valves γ(TiAl), Grade 2
	Ti-6Al-4V
	2Sn-4Zr-2Mo-0.1Si
Intake valves	intake valves Ti-6Al-4V
Turbocharger rotors	γ(TiAl)
Connecting rods	Ti-6Al-4V
Exhaust system	Grade 2

**Table 13:** Main applications of titanium and titanium alloys in car body panels and structure components, (*)—grade not specified.

Titanium and Titanium Alloys	Components	Reference
titanium alloys (*)	car door into the beam, car stop bracket	[332]
pure titanium (*)	shock absorber centre, door projecting beam, suspension systems	[333]
pure titanium (*)	panels (roof, hood)	[334]
pure titanium (*)	door beams	[329]
pure titanium (*)	car body frames	[330]
pure titanium (*)	crash elements	[301]
pure titanium (*)	active spoiler bracket, tailpipe trim covers (Bugatti)	[319]
pure titanium (*)	body panels (Thrust Super Sonic Car)	[335]
pure titanium (*)	muffler (Volkswagen Golf)	[330]
pure titanium (Grade 1)	The outer shell of the muffler (Bugatti Veyron 16.4)	[336]
pure titanium (Grade 2)	heat shields (Bugatti Veyron 16.4)	[336]
pure titanium (Grade 2)	exhaust system (Nissan Fair lady Z, Subaru Impreza, Koenigsegg)	[336]
pure titanium (Grade 2)	exhaust systems	[301]
pure titanium (Grade 2)	exhaust system (Bugatti Veyron)	[336]
pure titanium (Grade 4), Ti-6Al-4V	body panels	[301]
pure titanium (Grade 4)	car bodies	[337]
pure titanium (Grade 4), Ti-6Al-4V	car bodies	[337]
Ti-6Al-4V	bumpers	[301]
Ti-6Al-4V	armours	[337]
Ti-6Al-4V	exhaust systems (Chevrolet Corvette Z06)	[301]
Ti-6Al-4V	axle suspension, crash clamps (Bugatti Veyron 16.4)	[336]
Ti-6Al-4V, Ti-6.8Mo-4.5Fe-1.5Al	suspension springs	[337]
Ti-6Al-4V, Ti-6.8Mo-4.5Fe-1.5Al	suspension springs	[301]
Ti-5Al-2.5Sn	exhaust systems	[16]
Ti-6.8Mo-4.5Fe-1.5Al	suspension spring, (Bugatti Veyron 16.4, Ferrari 360 Stradale)	[336]
Ti-6.8Mo-4.5Fe-1.5Al	suspension springs (Volkswagen Lupo FSI)	[301]
Ti-1 Cu-1 Sn-0.35 Si-0.2 Nb, Ti-0.5Si-Fe (Ti-Exhaust XT), Ti-1Cu, Ti-0.1Fe-0.35Si, Ti-1.5Al, Ti-1 Cu-0.5 Nb, Ti-0.5Al-0.45Si-0.2Nb	exhaust systems	[317]
Ti-10V-2Fe-3Al, Ti-6Al-2Sn-4Zr-2Mo, Ti-6Al-4V, Ti-6Al-6V-2Sn, Ti- 8Al-1Mo-1V	high-strength performance components	[337]
Ti-1Cu-0.5Nb, Ti-0.45Si-0.25Fe, Ti-1.5Al, Ti-0.5Al-0.45Si-0.2Nb, Ti-0.9SA, Ti-1Cu-0.5Nb	mufflers	[331]

**Table 14:** Main applications of magnesium alloys in car body panels and structural components, (*)—grade not specified.

Magnesium Alloy	Components	Reference
A2401-002	steering wheel (Shanghai GMC)	[349]
AE44 (Mg-4Al-4RE)	engine bracket	[357]
AE44 (Mg-4Al-4RE)	cradle (Chevrolet Corvette Z06)	[358]
AM20 (*Mg*-2Al-0.5Mn), AM50 (Mg5.0Al0.3Mn)	seat frames (Mercedes Benz Roadster, Mercedes Benz SL500)	[359,360]
AM50 (Mg5.0Al0.3Mn)	seat frame, inside plate	[361]
AM50 (Mg5.0Al0.3Mn)	steering wheel	[362]
AM50 (Mg5.0Al0.3M)	car seat backrest	[363]
AM50 (Mg5.0Al0.3Mn)	trunk lid (Mercedes Benz E-series)	[349]
AM50 (Mg5.0Al0.3Mn)	cross car beam (Voyah Free)	[349]
AM50A (MgAl5Mn)	central control bracket (Volvo XC60)	[349]
AM50A (MgAl5Mn)	front- end carrier (Porsche Panamera G2)	[349]
AM50 (Mg5.0Al0.3Mn)	side door (Aston Martin DB9)	[364]
AM60B (Mg-6Al-0.4Mn)	cross car beam	[365]
AM60B (Mg-6Al-0.4Mn)	seat frame	[361]
AM60B (Mg-6Al-0.4Mn)	front-end carrier (Tesla S)	[366]
AM60B (Mg-6Al-0.4Mn)	inside door panel	[367]
AM60B (Mg-6Al-0.4Mn)	front-end frame	[368]
AM60B (Mg-6Al-0.4Mn)	folding roofs (Mercedes Benz SLK)	[349]
AM60B (Mg-6Al-0.4Mn)	body structure (Chrysler)	[369]
AM60B (Mg-6Al-0.4Mn)	front- end carrier (Range Rover)	[349,370]
AS41B (Mg-4Al-0.3Mn-1Si)	crankcase housing	[371]
AS41B (Mg-4Al-0.3Mn-1Si)	transmission housing	[372]
AS21 (MgAl2.2Si1Mn0.3), AS41 (MgAl4.5Si1Mn0.3)	transmission housing, crank case (Volkswagen)	[358]
AZ31 (Mg-3Al-1Zn-0.2Mn)	inner panel	[373]
AZ31B (Mg-3Al-1Zn)	upper rail (USAMP project)	[354]
AZ31B (Mg-3Al-1Zn)	Inner panel (Cadillac STS)	[374]
AZ31B-H24 (Mg-3Al-1Zn)	luggage retainer for Renault-Samsung SM7), roof panel (Porsche Carrera GT)	[354]
AZ61 (MgAl6Zn1)	column beam, luggage rack skeleton	[375]
AZ91 (MgAl9Zn1)	rear suspension, subframe members, clutch housing, transmission housing (Volvo LCP 2000)	[376]
AZ91 (MgAl9Zn1)	rear subframe (Audi A8)	[349]
AZ91B (Mg-2.5Al-0.7Zn-0.2Mn)	clutch Housing (Ford Ranger)	[360]
AZ91D (MgAl9Zn1(A))	inner panel (Ford F150)	[377]
AZ91D (MgAl9Zn1(A))	transmission housing	[378]
AZ91D (MgAl9Zn1(A))	steering column bracket	[379]
AZ91D (MgAl9Zn1(A))	clutch housing	[380]
AZ91D (MgAl9Zn1(A))	swivel plate	[381]
AZ91D (MgAl9Zn1(A))	central control bracket	[382]
AZ91D (MgAl9Zn1(A))	starter housing	[383]
AZ91D (MgAl9Zn1(A))	car dashboard member	[363]
AZ91D (MgAl9Zn1(A))	steering column (Ford Aerostar)	[360]
AZ91D (MgAl9Zn1(A))	truck gearbox housing (Volkswagen Passat, Audi A4 and A6)	[384]
cast magnesium alloy (*)	cabin bracket (Audi A8)	[349]
cast magnesium alloy (*)	roof frame, hardtop roof (Cadillac XLR)	[349]
cast magnesium alloy (*)	steering wheel (Dongfeng Nissan)	[349]
cast magnesium alloy (*)	tubular subframe (BMW 5- and 7-series)	[385]
cast magnesium alloy (*)	intake manifold cover (Audi A8)	[349]
cast magnesium alloy (*)	one-piece inner panel (Mercedes S-class)	[386]
cast magnesium alloy (*)	liftgate inner (Mercedes E-Class T)	[349]
cast magnesium alloy (*)	lift doors, side doors (Toyota Venza)	[387]
cast magnesium alloy (*)	side door, inner panels (Aston Martin Vanquish S)	[349]
cast magnesium alloy (*)	rear back doors (Jeep Wrangler)	[349]
cast magnesium alloy	bezel-less doors (Volkswagen)	[388]
magnesium alloys (*)	cross car beams (BMW X3, BMW X5, BMW X6, Chrysler Pacifica, Honda Ridgeline, Honda Acura MDX, Jaguar F-type, Jaguar XF, Jeep Grand Cherokee, Jeep Wrangler, Land Rover Discovery, Range Rover Evoque)	[389]
magnesium alloys (*)	lift gate inner (Chrysler Pacifica, Lincoln MKT), front deck (Dodge Viper, Mercedes AMG), ABS mounting bracket (Chrysler), trunk lid (Cadillac SLS), front end carrier (Tesla Model S)	[390]

## References

[1] Samek L., Krizan D. Steel—Material of choice for automotive lightweight applications. Proceedings of the International Conference Metal’2012.

[2] (1998). Ultra Light Steel Auto Body.

[3] Perka A.K., John M., Kuruveri U.B., Menezes P.L. (2022). Advanced high-strength steels for automotive applications: Arc and laser welding process, properties, and challenges. Metals.

[4] Kumar A., Singh A. (2021). Mechanical properties of nanostructured bainitic steels. Materialia.

[5] All-New 2021 Jeep® Grand Cherokee Breaks New Ground in the Full-Size SUV Segment. https://www.media.stellantis.com/uk-en/jeep/press/all-new-2021-jeep-grand-cherokee-breaks-new-ground-in-the-full-size-suv-segment-uk.

[6] Galán J., Samek L., Verleysen P., Verbeken K., Houbaert Y. (2012). Advanced high strength steels for automotive industry. Rev. Metal..

[7] Kuziak R., Kawalla R., Waengler S. (2008). Advanced high strength steels for automotive industry. Arch. Civ. Mech. Eng..

[8] Siczek K., Siczek K. (2017). Lekkie rozwiązania w przemyśle samochodowym. Autobusy.

[9] According to the Automotive and Transportation Market Research Report. https://mobilityforesights.com/product/automotive-ahss-market/.

[10] Schneider R., Heine B., Grant R.J., Waldemar A.M. (2014). Mechanical Behaviour of Commercial Aluminium Wrought Alloys at Low Temperatures, Light Metal Alloys Applications.

[11] Bielefeldt K., Papacz W., Walkowiak J. (2011). Environmentally friendly car plastics in automotive engineering. Arch. Motoryz..

[12] This Aluminum-Bodied 1953 Porsche 356 1500 Pre-A Cabriolet Is Shrouded in Mystery. https://www.hagerty.com/media/automotive-history/this-aluminum-bodied-1953-porsche-356-1500-pre-a-cabriolet-is-shrouded-in-mystery/.

[13] Choi C.H., Park S.S., Hwang T.W. (2001). Development of composite body panels for a lightweight vehicle. SAE Trans..

[14] Fantuzzi N., Bacciocchi M., Benedetti D., Agnelli J. (2021). The use of sustainable composites for the manufacturing of electric cars. Compos. Part C Open Access.

[15] Cieniek Ł. Anizotropia i Tekstura Krystalograficzna. Starzenie po Odkształceniu. https://docplayer.pl/56885357-Cwiczenie-nr-4-anizotropia-i-tekstura-krystalograficzna-starzenie-po-odksztalceniu.html.

[16] Sherman A.M., Allison J.E. (1986). Potential for Automotive Applications of Titanium Alloys.

[17] Titanium for Automotive Applications. https://www.azom.com/article.aspx?ArticleID=553.

[18] Schauerte O. (2003). Titanium in automotive production. Adv. Eng. Mater..

[19] Blicharski M. (2004). Inżynieria Materiałowa, Stal..

[20] Evaluation Technologies on High Strength Steel Sheet for Automobiles. https://www.kobelcokaken.co.jp/en/example/c/index.html.

[21] Senkara J. (2009). Współczesne stale karoseryjne dla przemysłu motoryzacyjnego i wytyczne technologiczne ich zgrzewania. Prz. Spaw..

[22] Best Surfaces for Cat Body Panels. https://www.thyssenkrupp-steel.com/en/industries/automotivetrucks/surfaces-for-car-body-panels/best-surfaces.html.

[23] Kubińska-Jabcoń E., Niekurzak M. (2019). Wykorzystanie nowoczesnych materiałów stosowanych w motoryzacji w celu poprawy jakości i bezpieczeństwa użytkowania pojazdów mechanicznych. Autobusy.

[24] Close D., Lallement R., Feuser P., Bold J. (2014). Challenges in Corrosion Protection for Press-Hardened Steels. Tagungsband Zum 9. Erlanger Workshop Warmblechumformung.

[25] Abotani K., Hirohata K., Kiyasu T. (2003). Hot-Dip Galvanized Sheet Steel with Excellent Press Formability and Surface Quality for the Automotive Panels. Kawasaki Steel Tech. Rep..

[26] (1994). Hot Dip Galvanized Coatings On Fabricated Iron And Steel Articles. Specifications and Test Methods.

[27] Close D. (2018). Alternative Protective Coatings for Hot Stamped Automotive Body Parts. Ph.D. Thesis.

[28] Ulbrich D., Kowalczyk J., Stachowiak A., Sawczuk W., Selech J. (2021). The Influence of Surface Preparation of the Steel during the Renovation of the Car Body on Its Corrosion Resistance. Coatings.

[29] Santos D., Raminhos H., Costa M.R., Diamantino T., Goodwin F. (2008). Performance of finish coated galvanized steel sheets for automotive bodies. Prog. Org. Coat..

[30] Wyrobek K. (2017). Modelowanie procesu tłoczenia części nadwozia samochodu ze stali superwysoko wytrzymałej. Inż. Masz..

[31] Horvath C.D., Mallick P.K. (2010). Advanced steels for lightweight automotive structures. Materials, Design and Manufacturing for Lightweight Vehicles.

[32] Grosman F., Piela A. (1991). Zastosowanie nowej metody badań do wstępnej oceny właściwości użytkowych blach dla motoryzacji. Inż. Mater..

[33] Trzepieciński T., Najm S.M. (2022). Application of artificial neural networks to the analysis of friction behaviour in a drawbead profile in sheet metal forming. Materials.

[34] Trzepieciński T., Szwajka K., Szewczyk M. (2023). Pressure-assisted lubrication of DC01 steel sheets to reduce friction in sheet-metal-forming processes. Lubricants.

[35] Kajal G., Tyagi M.R., Kumar G. (2023). A review on the effect of residual stresses in incremental sheet metal forming used in automotive and medical sectors. Mater. Today Proc..

[36] Ma L., Wang Z. (2021). The effects of through-thickness shear stress on the formability of sheet metal—A review. J. Manuf. Proc..

[37] Trzepieciński T., Lemu H.G. (2020). Improving prediction of springback in sheet metal forming using multilayer perceptron-based genetic algorithm. Materials.

[38] Szewczyk M., Szwajka K. (2023). Assessment of the tribological performance of bio-based lubricants using analysis of variance. Adv. Mech. Mater. Eng..

[39] Trzepieciński T., Najm S.M., Sbayti M., Belhadjsalah H., Szpunar M., Lemu H.G. (2021). New advances and future possibilities in forming technology of hybrid metal-polymer composites used in aerospace applications. J. Compos. Sci..

[40] Trzepieciński T., Najm S.M., Oleksik V., Vasilca D., Paniti I., Szpunar M. (2022). Recent developments and future challenges in incremental sheet forming of aluminium and aluminium alloy sheets. Metals.

[41] Żaba K., Puchlerska S., Kuczek Ł., Trzepieciński T., Maj P. (2023). Effect of step size on the formability of Al/Cu bimetallic sheets in single point incremental sheet forming. Materials.

[42] Rajarajan C., Sivaraj P., Sonar T., Raja S., Mathiazhagan N. (2022). Resistance spot welding of advanced high strength steel for fabrication of thin-walled automotive structural frames. Forces Mech..

[43] Ahmed M.M.Z., El-Sayed Selaman M.M., Fydrych D., Cam G. (2023). Review on friction stir welding of dissimilar magnesium and aluminum alloys: Scientometric analysis and strategies for achieving high-quality joints. J. Magnes. Alloys.

[44] Tomków J., Fydrych D., Rogalski G. (2020). Dissimilar underwater wet welding of HSLA steels. Int. J. Adv. Manuf. Technol..

[45] André V., Costas M., Langseth M., Morin D. (2023). Behavior and Large-Scale Modeling of Multi-Sheet Aluminum Connections With Self-Piercing Rivets. J. Manuf. Sci. Eng..

[46] Çavuşoğlu O., Bakırcı A., Dinkçi H., Yılmazoğlu A.G. (2022). Triple joining of different sheets with self-pierce riveting method. Sci. Technol. Weld. Join..

[47] Zhou Z.-J., Huang Z.-C., Jiang Y.-Q., Tang N.-L. (2022). Joining Properties of SPFC440/AA5052 Multi-Material Self-Piercing Riveting Joints. Materials.

[48] Cmorej D., Kaščák Ľ. (2023). Numerical simulation of mechanical joining of three DP600 and DC06 steel sheets. Adv. Mech. Mater. Eng..

[49] Huang Z.C., Huang G.H., Shan F.W., Jiang Y.Q., Zou Y.Q., Nie X.Y. (2023). Forming quality and microstructure evolution of AA6061-T6 aluminum alloy joint during flow drill screwing process. Adv. Eng. Mater..

[50] Kinsley B.L., Kinsley B.L., Wu X. (2011). 7—Tailor welded blanks for the automotive industry. Tailor Welded Blanks for Advanced Manufacturing.

[51] Keeler S., Kimchi M. (2014). Advanced High-Strength Steels. Application Guidelines Version 5.0, World Auto Steel. https://www.worldautosteel.org/projects/advanced-high-strength-steel-application-guidelines/.

[52] ULSAB-AVC. https://www.worldautosteel.org/projects/ulsab-avc-2/.

[53] ULSAC. https://www.worldautosteel.org/projects/ulsac-2/.

[54] ULSAS-UltraLight Steel Auto Suspensions Report. https://www.worldautosteel.org/ulsas-ultralight-steel-auto-suspensions-report/.

[55] FutureSteelVehicle. https://www.worldautosteel.org/projects/future-steel-vehicle/.

[56] Richter J., Kuhtz M., Hornig A., Harhash M., Palkowski H., Gude M. (2021). A mixed numerical-experimental method to characterize metal-polymer interfaces for crash applications. Metals.

[57] Structure-Borne-Damping Composite Material with Customized Properties. https://www.thyssenkrupp-steel.com/en/products/composite-material/overview-composite-material.html.

[58] Kustroń P., Korzeniowski M., Piwowarczyk T., Sokołowski P. (2021). Development of resistance spot welding processes of metal–plastic composites. Materials.

[59] Hybrix™ = ∑[214Lightweight, Formable, Strong, Eco-Friendly]. https://www.lamera.se/.

[60] Hammarberg S., Kajberg J., Larsson S., Moshfegh R., Jonsén P. (2021). Novel methodology for experimental characterization of micro-sandwich materials. Materials.

[61] Sokolova O.A., Kühn M., Palkowski H. (2012). Deep drawing properties of lightweight steel/polymer/steel sandwich composites. Arch. Civ. Mech. Eng..

[62] (2009). Cold Rolled Low Carbon Steel Flat Products for Cold Forming—Technical Delivery Conditions.

[63] Trzepieciński T., Szwajka K., Szewczyk M. (2023). An investigation into the friction of cold-rolled low-carbon DC06 steel sheets in sheet metal forming using radial basis function neural networks. Appl. Sci..

[64] Sudo M., Hashimoto S.I., Kambe S. (1983). Niobium bearing ferrite-bainite high strength hot-rolled sheet steel with improved formability. Trans. Iron Steel Inst. Jpn..

[65] Complex Phase Steels. https://automotive.arcelormittal.com/products/flat/first_gen_AHSS/CP.

[66] Dias E., Horimoto L., dos Santos Pereira M. (2014). Microstructural characterization of CP steel used in automotive industry. Mater. Sci. Forum.

[67] Santofimia M.J., van Bohemen S.M.C., Sietsma J. (2013). Combining bainite and martensite in steel microstructures for light weight applications. J. S. Afr. Inst. Min. Metall..

[68] Advanced High-Strength Steels—A Collision Repair Perspective. https://i-car.co.nz/wp-content/uploads/2018/10/general-technical-info-advanced-high-strength-steels-july-aug-2006.pdf.

[69] Chaurasiya R., Maji P., Mukhopadhyay G. (2023). High cycle fatigue behaviour of advanced high strength steel sheet (HS1000) in automotive application. Mater. Today Proc..

[70] Fonstein N. (2015). Complex phase steels. Advanced High Strength Sheet Steels.

[71] Graux A., Cazottes S., Castro D.D., San-Martín D., Capdevila C., Cabrera J.M., Molas S., Schreiber S., Mirković D., Danoix F. (2020). Design and development of complex phase steels with improved combination of strength and stretch-flangeability. Metals.

[72] Chu X., Zhao Y., Yang Y., Zhou F., Liu L., Zhao Z. (2023). Effect of recrystallization on bainite transformation and mechanical properties of complex phase steel with high formability (CH steel). J. Mater. Res. Technol..

[73] Hoile S. (2000). Processing and properties of mild interstitial free steel. Mater. Sci. Technol..

[74] Rana R., Bleck W., Singh S.B., Mohanty O.N. (2007). Development of high strength interstitial free steel by copper precipitation hardening. Mater. Lett..

[75] Da Rocha Santos A.P., Da Mata T.C., Segundo H.V.G., de Almeida L.H., Araújo L.S., da Cunha Rocha A. (2018). Texture, microstructure and anisotropic properties of IF-steels with different additions of titanium, niobium and phosphorus. J. Mater. Res. Technol..

[76] Zhang L., Zhi J., Mei F., Zhu L., Jiang X., Shen J., Cui J., Cai K., Thomas B.G. (2006). Basic oxygen furnace based steelmaking processes and cleanliness control at Baosteel. Ironmak. Steelmak..

[77] Zaitsev A.I., Rodionova I.G., Koldaev A.V. (2020). Study of methods for increasing ductility and formability of cold-rolled Ti-stabilized IF steels. IOP Conf. Ser. Mater. Sci. Eng..

[78] Ramazani A., Bruehl S., Gerber T., Bleck W., Prahl U. (2014). Quantification of bake hardening effect in DP600 and TRIP700 steels. Mater. Des..

[79] Oliaei M., Jamaati R. (2022). Improvement of the strength-ductility-toughness balance in interstitial-free steel by gradient microstructure. Mater. Sci. Eng. A.

[80] Tsuji N., Ito Y., Saito Y., Minamino Y. (2002). Strength and ductility of ultrafine grained aluminum and iron produced by ARB and annealing. Scr. Mater..

[81] Kuziak R. (2011). Technologia ciągłego wyżarzania blach cienkich. Pr. Inst. Metal. Żelaza.

[82] Pouranvari M., Marashi S.P.H. (2010). Key factors influencing mechanical performance of dual phase steel resistance spot welds. Sci. Technol. Weld. Join..

[83] Kuang S., Kang Y.L., Yu H., Liu R.D. (2009). Effect of continuous annealing parameters on the mechanical properties and microstructures of a cold rolled dual phase steel. Int. J. Miner. Metall. Mater..

[84] Dulucheanu C., Severin T.L., Cerlinca D.A., Irimescu L. (2022). Structures and Mechanical Properties of Some Dual-Phase Steels with Low Manganese Content. Metals.

[85] Shaw J., Zuidema B. (2001). New High Strength Steels Help Automakers Reach Future Goals for Safety, Affordability, Fuel Efficiency and Environmental Responsibility.

[86] Paul S.K. (2023). Effect of forming strain on low cycle, high cycle and notch fatigue performance of automotive grade dual phase steels: A review. Forces Mech..

[87] Llewellyn D.T., Hudd R.C. (1998). Steels—Metallurgy and Applications.

[88] Ding C., Liu J., Ning B., Huang M., Wu H. (2023). Enhanced strength-plasticity matching of lamellar 1 GPa-grade dual-phase steels via cyclic intercritical quenching. J. Mater. Res. Technol..

[89] Hilditch T.B., de Souza T., Hodgson P.D., Shome M., Tumuluru M.M. (2015). 2—Properties and automotive applications of advanced high-strength steels (AHSS). Welding and Joining of Advanced High Strength Steels (AHSS).

[90] Li Z., Chang Y., Rong J., Min J., Lian J. (2023). Edge fracture of the first and third-generation high-strength steels: DP1000 and QP1000. IOP Conf. Ser. Mater. Sci. Eng..

[91] Papadioti I., Bellas I., Tzini M.-I.T., Christodoulou P.I., Aravas N. (2020). TRIP Steels: A multiscale computational simulation and experimental study of heat treatment and mechanical behavior. Materials.

[92] Spišák E., Majerníková J., Kaščák Ľ., Mulidrán P., Rohaľ V., Bidulský R. (2022). Experimental and numerical thickness analysis of TRIP steel under various degrees of deformation in bulge test. Materials.

[93] Pantilimon M.C., Berbecaru A.C., Gherghescu I.A., Coman G., Ciucă S., Grecu A., Sohaciu M.G., Dumitrescu R.E., Predescu C. (2022). Comparative Evaluation of the TRIP Effect in Steels with Different Contents of Mn and Al. Metals.

[94] Campbell J. (2022). The Hot Ductility of TWIP and TRIP Steels—An Alternative Interpretation. Metals.

[95] Krizan D. TRIP steels: Advanced high strength multiphase steels for automotive applications. Proceedings of the 14th International Scientific Conference COM-MATTECH.

[96] Hu X., Feng Z. (2021). Advanced High-Strength Steel—Basics and Applications in the Automotive Industry.

[97] Chen L., Zhao Y., Qin X. (2013). Some aspects of high manganese twinning-induced plasticity (TWIP) steel: A review. Acta Metall..

[98] Dobrzański L.A. (2006). Materiały Inżynierskie i Projektowanie Materiałowe.

[99] Imandoust A., Hanzaki A.Z., Heshmati-Manesh S., MoMoemeni S., Changizian M.P. (2014). Effects of ferrite volume fraction on the tensile deformation characteristics of dual phase twinning induced plasticity steel. Mater. Des..

[100] Kliber J., Kursa T., Schindler I. (2008). Hot rolling of steel with TWIP effect. Hut.—Wiad. Hut..

[101] Gołaszewski A. (2015). Nowa generacja stali ultradrobnoziarnistych z efektem TRIP. Stal Met. Nowe Technol..

[102] Park J., Kang M., Sohn S.S., Kim J.S., Kim H.S., Cho W.T., Lee S. (2017). Tensile properties of cold-rolled TWIP-cored three-layer steel sheets. Mater. Sci. Eng. A.

[103] Gronostajski Z., Kuziak R. (2010). Metalurgiczne, technologiczne i funkcjonalne podstawy zaawansowanych wysokowytrzymałych stali dla przemysłu motoryzacyjnego. Pr. Inst. Metal. Żelaza.

[104] Ghosh M., Ghosh A., Roy A. (2019). Renewable and sustainable materials in automotive industry. Encyclopedia of Renewable and Sustainable Materials.

[105] Song H., Kwon Y., Sohn S.S., Koo M., Kim N.J., Lee B.J., Lee S. (2018). Improvement of tensile properties in (austenite+ferrite+κ-carbide) triplex hot-rolled lightweight steels. Mater. Sci. Eng. A.

[106] Sozańska-Jędrasik L., Mazurkiewicz J., Borek W., Dobrzański L.A. (2018). Structure and mechanical properties of newly-developed high-strength TRIPLEX type steel. Inż. Mater..

[107] Frommeyer G., Brüx G. (2006). Microstructure and mechanical properties of high-strength Fe-Mn-Al-C TRIPLEX steels. Steel Res. Int..

[108] Moon J., Ha H.Y., Kim K.W., Park S.J., Lee T.H., Kim S.D., Jang J.H., Jo H.H., Hng H.U., Lee B.H. (2020). A new class of lightweight, stainless steels with ultra-high strength and large ductility. Sci. Rep..

[109] Kalantari A.R., Zarei-Hanzaki A., Abedi H.R., Jalali M.S., Park S.J., Park J.Y. (2021). The high temperature deformation behavior of a triplex (ferrite+ austenite+ martensite) low density steel. J. Mater. Res. Technol..

[110] Sohn S.S., Choi K., Kwak J.H., Kim N.J., Lee S. (2014). Novel ferriteeaustenite duplex lightweight steel with 77% ductility by transformation induced plasticity and twinning induced plasticity mechanisms. Acta Mater..

[111] Abedi H.R., Hanzaki A.Z., Ou K.L., Yu C.H. (2017). Substructure hardening in duplex low density steel. Mater. Des..

[112] Zhang J., Hu C., Liu Y., Zhang Y., Song C., Zhai Q. (2023). Microstructure, mechanical properties and deformation behavior of Cr-containing triplex low-density steels with different C content. J. Mater. Res. Technol..

[113] Black J.T., Kohser R.A. (2012). Materials and Processes in Manufacturing.

[114] Venezuela J., Lim F.Y., Liu L., James S., Zhou Q., Knibbe R., Zhang M., Li H., Dong F., Dargusch M.S. (2020). Hydrogen embrittlement of an automotive 1700 MPa martensitic advanced high-strength steel. Corros. Sci..

[115] Liu Q., Atrens A. (2023). A critical review of the influence of hydrogen on the mechanical properties of medium strength steels. Corros. Rev..

[116] Venezuela J., Blanch J., Zulkiply A., Liu Q., Zhou Q., Zhang M., Atrens A. (2018). Further study of the hydrogen embrittlement of martensitic advanced high-strength steel in simulated auto service conditions. Corros. Sci..

[117] Nagumo M., Takai K. (2019). The predominant role of strain-induced vacancies in hydrogen embrittlement of steels: Overview. Acta Mater..

[118] Venezuela J., Hill T., Zhou Q., Li H., Shi Z., Dong F., Knibbe R., Zhang M., Dargusch M.S., Atrens A. (2021). Hydrogen-induced fast fracture in notched 1500 and 1700 MPa class automotive martensitic advanced high-strength steel. Corros. Sci..

[119] Djukic M.B., Bakic G.M., Zeravcic V.S., Sedmak A., Rajicic B. (2019). The synergistic action and interplay of hydrogen embrittlement mechanisms in steels and iron: Localized plasticity and decohesion. Eng. Fract. Mech..

[120] Lynch S. (2012). Hydrogen embrittlement phenomena and mechanisms. Corros. Rev..

[121] Birnbaum H.K., Sofronis P. (1994). Hydrogen-enhanced localized plasticity—A mechanism for hydrogen related fracture. Mater. Sci. Eng. A.

[122] Tong Y., Li W., Zhou Q., Li J. (2023). Rapid evaluation of the critical condition for hydrogen-induced cracking in ultrahigh-strength automotive steel sheets: A semiquantitative investigation based on U-bend specimens. Eng. Fail. Anal..

[123] Hall J.N., Fekete J.R., Rana R., Singh S.B. (2017). Steels for auto bodies: A general overview. Automotive Steels.

[124] Press Hardening Steels (PHS) for Complex Shapes. https://www.ssab.com/en/brands-and-products/docol/automotive-steel-grades/press-hardening-steel.

[125] Press Hardened Steels (PHS). https://www.totalmateria.com/page.aspx?ID=CheckArticle&site=kts&LN=PL&NM=565.

[126] Fonstein N. (2015). Advanced high strength sheet steels. Advanced High Strength Sheet Steels.

[127] Speer J.G., De Moor E., Findley K., Matlock D.K., De Cooman B.C., Edmonds D.V. (2011). Analysis of microstructure evolution in quenching and partitioning automotive sheet steel. Met. Mater. Trans. A.

[128] Schmitt J.H., Iung T. (2018). New developments of advanced high-strength steels for automotive applications. C. R. Phys..

[129] Speer J.G., Assunção F.C.R., Matlock D.K., Edmonds D.V. (2005). The “quenching and partitioning” process: Background and recent progress. Mater. Res..

[130] Hidalgo J., Celada-Casero C., Santofimia M. (2019). Fracture mechanisms and microstructure in a medium Mn quenching and partitioning steel exhibiting macrosegregation. Mater. Sci. Eng. A.

[131] Wang L., Speer J.G. (2013). Quenching and partitioning steel heat treatment. Metallogr. Microstruct. Anal..

[132] Pierce D.T., Coughlin D.R., Clarke K.D., De Moor E., Poplawsky J., Williamson D.L., Mazumder B., Speer J.G., Hood A., Clarke A.J. (2018). Microstructural evolution during quenching and partitioning of 0.2C-1.5Mn-1.3Si steels with Cr or Ni additions. Acta Mater..

[133] Carpio M., Calvo J., García O., Pedraza J.P., Cabrera J.M. (2021). Heat treatment design for a QP steel: Effect of partitioning temperature. Metals.

[134] Horvath C.D., Enloe C.M. Opportunities and Challenges for 3rd Generation Advanced High-Strength Steels in Automotive Body Structures. Presented at 2017 Great Designs in Steel, Sponsored by American Iron and Steel Institute. http://www.sawchina.cn/en/Automobilemanufacturing/708.mhtml.

[135] Ford’s Hot New Bronco Built with Alabama Steel. https://www.al.com/news/mobile/2020/11/fords-hot-new-bronco-built-with-alabama-steel.html.

[136] (2014). Stainless Steels—Part 1: List of Stainless Steel.

[137] Huang G.L., Matlock D.K., Krauss G. (1989). Martensite formation, strain rate sensitivity, and deformation behavior of type 304 stainless steel sheet. Metall. Trans. A Phys. Metall. Mater. Sci..

[138] Xu L., Barlat F., Ahn D.C., Bressan J.D. (2011). Forming limit and fracture mechanism of ferritic stainless steel sheets. Mat. Sci. Eng. A-Struct..

[139] Arrayago I., Real E., Gardner L. (2015). Description of stress–strain curves for stainless steel alloys. Mater. Des..

[140] Podatność Stali Nierdzewnych na Przeróbkę Plastyczną. http://www.stalenierdzewne.pl/84/podatnosc-stali-nierdzewnych-na-przerobke-plastyczna.

[141] Zastosowanie Stali Nierdzewnej w Produkcji Samochodów Osobowych. https://www.stalenierdzewne.pl/1076/zastosowanie-stali-nierdzewnej-w-produkcji-samochodow-osobowych.

[142] Nam Y.H., Park J.S., Baek U.B., Suh J.Y., Nahm S.H. (2019). Low-temperature tensile and impact properties of hydrogen-charged high-manganese steel. Int. J. Hydrogen Energy.

[143] Mishnev R., Borisova Y., Kniaziuk T., Gaidar S., Kaibyshev R. (2023). Quench and Tempered Embrittlement of Ultra-High-Strength Steels with Transition Carbides. Metals.

[144] Tsuboi M., Shibata A., Terada D., Tsuji N. (2017). Role of Different Kinds of Boundaries Against Cleavage Crack Propagation in Low-Temperature Embrittlement of Low-Carbon Martensitic Steel. Met. Mater. Trans. A.

[145] Song R., Ponge D., Raabe D. (2005). Mechanical Properties of an Ultrafine Grained C-Mn Steel Processed by Warm Deformation and Annealing. Acta Mater..

[146] Tsuji N., Okuno S., Koizumi Y., Minamino Y. (2004). Toughness of Ultrafine Grained Ferritic Steels Fabricated by ARB and Annealing Process. Mater. Trans..

[147] Fahlblöm P. (1997). Analys för val av Emballagesystem: En Studie Gjord för att Underlätta Valet av Emballagesystem (Analysis for Choice of Packaging: A Study Done on Choosing of Packaging Methods, in Swedish). Master’s Thesis.

[148] Bano X., Laurent J.P. Heat treated boron steels in the automotive industry. Proceedings of the 39th Mechanical Working and Steel Processing Conference.

[149] Reinhardt A. Development of hot stamped Ultra High Strength Steel parts on the Peugeot 307 and the Citroën C5. Proceedings of the EuroCarBody 2001—3rd Global Car Body Benchmarking Conference.

[150] Weigert P. Challenges in mass production of press hardened components focusing CO_2_ reduction. Proceedings of the Insight Edition Conference.

[151] Fidorra A., Baur J. The Art of Progress: Audi—The New A8. Proceedings of the EuroCarBody 2010.

[152] Wuhan Economic and Technological Development Zone, Dongfeng Moves Up-Market in Electric Vehicle Segment. http://en.whkfq.gov.cn/2020-11/11/c_566509.htm.

[153] Lanzerath H., Bach A., Oberhofer G., Gese H. (2007). Failure Prediction of Boron Steels in Crash.

[154] Lapsien R. Hot Forming at Benteler—Current applications and trends for the future. Proceedings of the Materials in Car Body Engineering 2014.

[155] Taylor T., Clough A. (2018). Critical review of automotive hot-stamped sheet steel from an industrial perspective. Mater. Sci. Technol..

[156] Staeves J. Höherfeste Stähle für die Karosserie (High strength steels for car body—In German). Proceedings of the Technical University of Munich.

[157] Gier A. (2005). Hot Forming and Hardening of Rollformed Sections. Aluminum and Steel Forming in Automotive Engineering.

[158] Lindberg H. Advanced High Strength Steel Technologies in the 2016 Volvo XC90. Presented at 2016 Great Designs in Steel, Sponsored by American Iron and Steel Institute. https://docplayer.net/42205170-Advanced-high-strength-steel-technologies-in-the-2016-volvo-xc90.html.

[159] Kröning A. Lightweight solutions for body applications. Proceedings of the Insight Edition Conference.

[160] Pfestorf M., Rensburg J. (2006). Functional Properties of High Strength Steel in Body in White.

[161] Hilfrich E., Seidner D. Crash safety with high strength steels. Proceedings of the International Automotive Congress.

[162] Pfestorf M. Multimaterial lightweight design for the body in white of the new BMW 7 series. Proceedings of the International Conference of Innovative Developments for Lightweight Vehicle Structures.

[163] Billur E. (2019). Hot Stamping of Ultra High-Strength Steels: From a Technological and Business Perspective.

[164] Ludlow D. (2012). Ford B-Max preview. Expert Reviews.

[165] PHS Automotive Applications and Usage. https://ahssinsights.org/forming/press-hardened-steels/phs-automotive-applications-and-usage/.

[166] Horner K. (2018). Strategic Steel Application in the Acura NSX Space Frame.

[167] Büchner J., Oberlander T., Benneker B. Ford Focus. Proceedings of the EuroCarBody 2018.

[168] Vaissiere L., Laurent J.P., Reinhardt A. (2002). Development of Pre-Coated Boron Steel for Applications on PSA Peugeot Citroën and RENAULT Bodies in White.

[169] Plassart G., Philip G. (2002). Materials Criteria Selection and Certification Process for the Body in White in PSA PEUGEOT CITROËN.

[170] Decker L., Truskin J. The All-New 2017 Chrysler Pacifica. Proceedings of the Strategies in Car Body Engineering 2017.

[171] Matsuoka H., Fujihara K. Mazda CX-5. Proceedings of the EuroCarBody 2011.

[172] VAMA China When Usibor®2000 Meets China’s Legendary SUV model Haval H6. http://www.vamachina.com/en/usibor2000-meets-haval-h6/.

[173] Bernquist J. Safety Cage Design in the Volvo XC90. http://worldautosteel.wpengine.com/wp-content/uploads/GDIS-Prez/B-44_22%20-%20Safety%20Cage%20Design%20in%20the%20Volvo%20XC90-2004.pdf.

[174] Lüken I., Tenneberg N. Volkswagen ID.3. Proceedings of the Aachener Karosserietage.

[175] Press Hardened Steels. https://ahssinsights.org/metallurgy/steel-grades/phs-grades/.

[176] D’Aiuto F., Tedesco M.M. Development of New Structural Components with Innovative Materials and Technological Solutions. Proceedings of the Materials in Car Body Engineering 2015.

[177] Tobita S., Shinmiya T., Yamasaki Y., Hiramoto J. (2021). Development of Forming Technology to Reduce Dimensional Scattering of Automotive Parts with Cambers by Using Bauschinger Effect. Mater. Trans..

[178] Reed D., Belanger P. Hot Stamped Steel One-Piece Door Ring in the All-New 2019 Ram 1500. https://www.steel.org/wp-content/uploads/2021/02/Track-2-Belanger-and-Reed.pdf.

[179] Fossati B., Machado-Baglietto A., Cappelaere M. Hot Stamping Industrialization at Renault. Proceedings of the Forming in Car Body Engineering 2014.

[180] Black S., Rowlings S., Dietrich C. Jaguar I-PACE. Proceedings of the EuroCarBody 2018.

[181] Bilur E., Rana R., Singh S.B. (2017). Hot formed Steels. Automotive Steels Design, Metallurgy, Processing and Applications.

[182] Transformation Induced Plasticity (TRIP). https://ahssinsights.org/metallurgy/steel-grades/3rdgen-ahss/transformation-induced-plasticity-trip/.

[183] Christodoulou P.I. (2017). Effect of Retained Austenite Transformation on the Fatigue Behaviour of Aluminum Containing TRIP Steels. Ph.D. Thesis.

[184] Martensite. https://ahssinsights.org/metallurgy/steel-grades/1stgen-ahss/martensite/.

[185] JFE Steel Corporation (2021). Toyota Lexus Adopts 1.5 GPa High-Strength Cold-Rolled Steel Sheet to Structural Part by Unique Cold Press Forming Technology.

[186] Krajewski S., Nowacki J. (2011). Mikrostruktura i właściwości stali o wysokiej wytrzymałości AHSS. Prz. Spaw..

[187] Dual Phase. https://ahssinsights.org/metallurgy/steel-grades/ahss/dual-phase/.

[188] Complex Phase. https://ahssinsights.org/metallurgy/steel-grades/complex-phase-steel/.

[189] Ferrite-Bainite. https://ahssinsights.org/metallurgy/steel-grades/ferrite-bainite-steel/.

[190] Nissan Japan Motors Infiniti QX50. Proceedings of the EuroCarBody 2001—20th Global Car Body Benchmarking Conference.

[191] 3rd Generation Steels. https://ahssinsights.org/metallurgy/steel-grades/3rd-generation-steels/.

[192] Coakley D. (2015). 2015 Nissan Murano.

[193] Coakley D., Zischke J. (2015). The 2016 Nissan Maxima.

[194] Wang L., Bian J., Wang J., Ye Y. Development and Application of New Generation AHSS Based on Q&P Process. Proceedings of the Materials in Car Body Engineering 2019.

[195] Twinning Induced Plasticity. https://ahssinsights.org/metallurgy/steel-grades/ahss/twinning-induced-plasticity/.

[196] Nam J.B. Development of New Auto Steels and Application Technology. https://docplayer.net/48872013-Development-of-new-auto-steels-and-application-technology.html.

[197] D’Aiuto F. Innovative materials and solutions for automotive components. Proceedings of the ANFIA—Associazione Nazionale Fra Industrie Automobilistiche (National Association of the Automobile Industry).

[198] Renault Press Release EOLAB Concept Showcases Renault’s Pursuit of Ultra-Low Fuel Consumption. https://www.press.renault.co.uk/en-gb/releases/1707.

[199] Capelli F., Boneschi V., Viganò P., Inox C. Stainless Steel: A new structural automotive material. Proceedings of the 9th International Conference & Exhibition, FLORENCE ATA 2005.

[200] Applications and Uses of Stainless Steel in Automotive Industry. https://www.vishwastainless.com/stainless-steel-applications-and-uses-in-automotive-industry/.

[201] Snelgrove P. Stainless Steel Automotive and Transport Developments. https://www.worldstainless.org/Files/issf/non-image-files/PDF/Stainlesssteelautomotiveandtransportdevelopments.pdf.

[202] Stainless Steel Applications—Automotive. https://www.worldstainless.org/Files/issf/non-image-files/PDF/Automotiveapplications.pdf.

[203] Application of Stainless Steel in Automobile Industry. https://www.ronscopipe.com/infodetail/application-of-stainless-steel-in-automobile-industry.html.

[204] Podder A.S., Bhanja A. (2013). Applications of stainless steel in automobile industry. Adv. Mater. Res..

[205] Emmons J.E., Blessing L.J. (2001). Ultra-light Stainless Steel Urban Bus Concept.

[206] Kwiatkowski L. (2009). Podatność na korozję i skuteczność aktualnych metod ochrony przed korozją stopów aluminium stosowanych w budownictwie. Inż. Powierz..

[207] Callister W.D., Rethwisch D.G. (2018). Materials Science and Engineering: An Introduction.

[208] Kondratiuk J., Kuhn P. (2011). Tribological investigation on friction and wear behaviour of coatings for hot sheet metal forming. Wear.

[209] Davies J.R. (2014). ASM Specialty Handbook: Aluminum and Aluminum Alloys.

[210] (2005). Aluminium and Aluminium Alloys—Chemical Composition and Form of Wrought Products—Part 3: Chemical Composition and Form of Products.

[211] Davis J.R., Davis J.R. (2001). Aluminium and aluminium alloys. Alloying: Understanding the Basics.

[212] Djukanovic G. Aluminium Alloys in the Automotive Industry: A Handy Guide. https://aluminiuminsider.com/aluminium-alloys-automotive-industry-handy-guide/.

[213] Huber G., Djurdjevic M.B., Manasijevic S. (2019). Determination some thermo-physical and metallurgical properties of aluminum alloys using their known chemical composition. Int. J. Heat Mass Transf..

[214] Vijayakumar M.D., Dhinakaran V., Sathish T., Muthu G., Bupathiram P.M. (2021). Experimental study of chemical composition of aluminium alloys. Mater. Today Proc..

[215] Hattori C.S., Almeida G.F.C., Gonçalves R.L.P., Santos R.G., Souza R.C., da Silva W.C., Cunali J.R.C., Couto A.A. (2021). Microstructure and fatigue properties of extruded aluminum alloys 7046 and 7108 for automotive applications. J. Mater. Res. Technol..

[216] Puga H. (2020). Casting and forming of advanced aluminum alloys. Metals.

[217] Stojanovic B., Bukvic M., Epler I. (2018). Application of aluminum and aluminum alloys in engineering. Appl. Eng. Lett. J. Eng. Appl. Sci..

[218] Wang B., Zhang Z., Xu G., Zeng X., Hu W., Matsubae K. (2023). Wrought and cast aluminum flows in China in the context of electric vehicle diffusion and automotive lightweighting. Resour. Conserv. Recycl..

[219] Baser T.A., Umay E., Akinci V. (2022). New Trends in Aluminum Die Casting Alloys for Automotive Applications. Eurasia Proc. Sci. Technol. Eng. Math..

[220] Ducker C. Mega-Casting Trends for Automotive Manufacturers in 2022. https://www.linkedin.com/pulse/mega-casting-trends-automotive-manufacturers-2022-ducker-worldwide.

[221] Aluminium in Transport. https://www.aluminiumleader.com/application/transport/.

[222] Szczucka-Lasota B., Węgrzyn T., Jurek A. (2020). Aluminum alloy welding in automotive industry. Transp. Probl..

[223] Kaufman J.G. (2000). Applications for aluminum alloys and tempers. Introd. Alum. Alloys Tempers.

[224] Understanding the Alloys of Aluminium. http://www.alcotec.com/us/en/education/knowledge/techknowledge/understanding-the-alloys-of-aluminum.cfm.

[225] Benedyk J.C., Mallick P.K. (2010). Aluminum alloys for lightweight automotive structures. Materials, Design and Manufacturing for Lightweight Vehicles Materials, Design and Manufacturing for Lightweight Vehicles.

[226] The Aluminum Association Auto & Light Truck Group (ALTG). https://www.southadams.k12.in.us/site/Default.aspx?PageType=3&DomainID=101&PageID=531&ViewID=5c8b25c6-c8f8-4bd5-923b-8a7c70a93dda&FlexDataID=1671.

[227] Fridlyander I.N., Sister V.G., Grushko O.E., Berstenev V.V., Sheveleva L.M., Ivanova L.A. (2002). Aluminum alloys: Promising materials in the automotive industry. Met. Sci. Heat Treat..

[228] Why Are Aluminum Alloys Used in the Auto Industry?. https://www.howardprecision.com/why-are-aluminum-alloys-used-in-the-auto-industry/.

[229] Rooy E.L. (1990). Introduction to Aluminum and Aluminum Alloys. ASM Handbook Committee. Volume 2: Properties and Selection: Nonferrous Alloys and Special-Purpose Materials.

[230] Mukhopadhyay P. (2012). Alloy designation, processing, and use of AA6XXX series aluminium alloys. Int. Sch. Res. Not..

[231] Aluminum Alloys Used in the Automotive Industry!. https://www.metalsupermarkets.com/aluminum-alloys-used-in-the-automotive-industry/.

[232] What Is Aluminum Used for: Automotive Edition. https://www.kloecknermetals.com/blog/what-is-aluminum-used-for-automotive-edition/.

[233] Polmear I.J. (2005). Light Alloys: From Traditional Alloys to Nanocrystals.

[234] Constellium Supplying Aluminum Solutions for Audi E-Tron GT EV. Constellium Supplying Aluminum Solutions for Audi E-Tron GT EV. Retrieved 2023-3-31.

[235] Applications—Car Body—Body Structures. https://european-aluminium.eu/wp-content/uploads/2022/11/1_aam_body-structures.pdf.

[236] Koganti R., Weishaar J. (2009). Aluminum Vehicle Body Construction and Enabling Manufacturing Technologies. SAE Int. J. Mater. Manuf..

[237] Applications—Car Body—Body Components. https://european-aluminium.eu/wp-content/uploads/2022/11/2_aam_body-components.pdf.

[238] Hofer-Hauser P., Gschwandtner R. Influence of Die Evacuation on Mechanical Properties and Heat Treatability of HPD-Castings. https://www.fondarex.com/media/fdx_hofer_gschwandtner_en.pdf.

[239] Luo A.A., Sachdev A.K., Apelian D. (2022). Alloy Development and Process Innovations for Light Metals Casting. J. Mater. Process. Technol..

[240] Hartlieb M. (2013). Aluminum Alloys for Structural Die Casting. Die Cast. Eng..

[241] Pezda J., Jezierski J. (2020). Non-Standard T6 Heat Treatment of the Casting of the Combustion Engine Cylinder Head. Materials.

[242] (2021). Aluminium in Cars: Unlocking the Lightweighting Potential.

[243] Applications—Car Body—Roof and Trim. https://european-aluminium.eu/wp-content/uploads/2022/11/5_6_aam_roof-and-trim.pdf.

[244] Applications—Car Body—Crash Management Systems. https://european-aluminium.eu/wp-content/uploads/2022/11/4_aam_crash-management-systems1.pdf.

[245] Rheinfelden Alloys GmbH & Co. KG. (2015). Alloys Alloys for High Pressure Die Casting.

[246] Burger G.B., Gupta A.K., Jeffrey P.W., Lloyd D.J. (1995). Microstructural control of aluminum sheet used in automotive applications. Mater. Charact..

[247] Application of Aluminum Alloy in Automobile Manufacturing. https://hw-alu.com/blog/application-of-aluminum-alloy-in-automobile-manufacturing.html.

[248] Uno T., Baba Y. (1987). Neue Aluminiumlegierung für Karosseriebleche. Aluminium.

[249] Applications—Car Body—Hang-On Parts. https://european-aluminium.eu/wp-content/uploads/2022/11/3_aam_hang-on-parts.pdf.

[250] Types and Applications of Aluminum Alloys for Vehicles. https://uacj-automobile.com/types_and_applications.html.

[251] Miller W.S., Zhuang L., Bottema J., Wittebroad A.J., De Smet P., Haszler A., Vieregge A. (2000). Recent development in aluminium alloys for the automotive industry. Mater. Sci. Eng. A.

[252] What Are the Aluminum Alloys Used in Cars. https://www.autoaluminumsheet.com/a/what-are-the-aluminum-alloys-used-in-cars.html.

[253] Olandersson H. Novelis—Overview of Flat Rolled Aluminium Products. https://swedsoft.se/wp-content/uploads/sites/24/2016/12/161108_Novelis-presentation-SAMS.pdf.

[254] Panda S.S. (2015). Aluminum Alloys in Automotive Application.

[255] Baruah M., Borah A. (2020). Processing and precipitation strengthening of 6xxx series aluminium alloys: A review. Int. J. Mater. Sci..

[256] Couper M.J., Edwards G.A. (2013). 6xxx Series Aluminium Alloy. Canadian Patent.

[257] Zupanič F., Klemenc J., Steinacher M., Glodež S. (2023). Microstructure, mechanical properties and fatigue behaviour of a new high-strength aluminium alloy AA 6086. J. Alloys Compd..

[258] Lin C.W., Hung F.Y., Lui T.S. (2017). Microstructural characteristics and mechanical behaviors of new type SIMA processed aluminum alloy. Aluminium Alloys—Recent Trends in Processing, Characterization, Mechanical Behavior and Applications.

[259] Wang C., Wang Z., Xu H., Zhang G. (2022). Decreased dislocation density as an origin for the quench sensitivity of the Al-Si-Mg alloys with high Si kontent. J. Alloys Compd..

[260] Chakrabarti D., Laughlin D.E. (2004). Phase relations and precipitation in Al–Mg–Si alloys with Cu additions. Prog. Mater. Sci..

[261] Wu Y., Wang H., Ban C. (2021). Effect of Fe content on the microstructure and properties of hot-extruded 6061 aluminum alloy. J. Phys. Conf. Ser..

[262] Lin X.Z., Yin F., Sun B.D. (1999). lnfluence of Fe on the properties of Al Si alloy and methods of neutralizing the effect of Fe. Foundry Technol..

[263] Xu Z., Zhang X.Y., Wang H.B., Gao A., Ma T., Song H. (2020). Effect of Mn/Fe ratio on the microstructure and properties of 6061 sheets obtained by twin-roll cast. Mater. Charact..

[264] Kaufman J.G. (2019). Corrosion of aluminum and aluminum alloys. Properties and Selection of Aluminum Alloys.

[265] Muzykiewicz W., Rękas A., Kosmalski G. (2006). Badania walidacyjne blachy w gatunku 6082 w stanie ‘0’ pod kątem jej zastosowań do procesów tłoczenia. Rudy Met. Nieżelazne.

[266] Rochet C., Veron M., Raucvh E.F., Lowe T.C., Arfaei B., Laurino A., Harouard J.P., Blanc C. (2020). Influence of equal-channel angular pressing on the microstructure and corrosion behaviour of a 6xxx aluminium alloy for automotive conductors. Corros. Sci..

[267] Oana S.A., Karancsi O., Mitelea I., Uţu I.D., Craciunescu C.M. (2023). The role of filler material selection in the laser welding process of deformable 6xxx series aluminum alloys. Mater. Today Proc..

[268] Hirsch J. (1997). Aluminium alloys for automotove application. Mater. Sci. Forum.

[269] Novelis and Jaguar Land Rover to Supply Lightweight SUVs. https://www.ai-online.com/2012/11/novelis-and-jaguar-land-rover-to-supply-lightweight-suvs/.

[270] Sun J. (2018). Research on Situation and Application Prospect of Automotive Body Sheets Al-Mg-Si Based (6000series) Alloy. IOP Conf. Ser. Mater. Sci. Eng..

[271] Swapna D., Rao C.S., Kumar D.S., Radhika S. (2019). AHP and TOPSIS based selection of aluminium alloy for automobile panels. J. Mech. Energy Eng..

[272] Tisza M., Lukács Z. (2018). High strength aluminum alloys in car manufacturing. IOP Conf. Ser. Mater. Sci. Eng..

[273] Li Y. (2019). Effect of Alloy Elements on Microstructure and Hot Tearing Susceptibility in Direct-Chill Casting of 7xxx Series Aluminum Alloys. Ph.D. Thesis.

[274] Dai Y., Yan L., Hao J. (2022). Review on micro-alloying and preparation method of 7xxx series aluminum alloys: Progresses and prospects. Materials.

[275] Bhuiyan M.S., Toda H., Uesugi K., Takeuchi A., Watanabe Y. (2020). Damage micromechanisms in high Mn and Zn content 7xxx series aluminum alloys. Mater. Sci. Eng. A.

[276] Valeev I.S., Barykin N.P., Trifonov V.G., Valeeva A.K. (2014). Effect of powerful current pulses on the structure and mechanical properties of the aluminum alloy Al-6%Mg-0.6%Mn. J. Mater. Eng. Perform..

[277] Fang H.C., Yang H.L., Zhu J.M., Xiao P., Chen Z., Liu T. (2020). Effect of minor Cr, Mn, Zr or Ti on recrystallization, secondary phases and fracture behaviour of Al-Zn-Mg-Cu-Yb Alloys. Rare Met. Mater. Eng..

[278] Li G.F., Zhang X.M., Zhu H.F. (2010). Effect of minor Er and Y additions to Al-Zn-Mg-Cu-Zr alloy on homogenizing behavior. Hangkong Cailiao Xuebao/J. Aeronaut. Mater..

[279] Long R.S., Boettcher E., Crawford D. (2017). Current and future uses of aluminum in the automotive industry. JOM.

[280] Kumar M., Poletti C., Degischer H.P. (2013). Precipitation kinetics in warm forming of AW-7020 alloy. Mater. Sci. Eng. A.

[281] Polak S., Kaczyński P., Gronostajski Z., Jaśkiewicz K., Krawczyk J., Skwarski M., Zwierzchowski M., Chorzępa W. (2017). Warm forming of 7075 aluminum alloys. Procedia Eng..

[282] Jaśkiewicz K., Skwarski M., Kaczyński P., Gronostajski Z., Polak S., Trzpis P. (2022). Warm sheet metal forming of en-ergy-absorbing elements made 7075 aluminum alloy in the hardened state T6. Int. J. Adv. Manuf. Technol..

[283] Shin J., Kim T., Kim D., Kim D., Kim K. (2017). Castability and mechanical properties of new 7xxx aluminum alloys for automotive chassis/body applications. J. Alloys Compd..

[284] Svendsen A. Aluminum Continues Unprecedented Growth in Automotive Applications—Light Metal Age Magazine. Light Metal Age Magazine 2020. https://www.lightmetalage.com/news/industry-news/automotive/aluminum-continues-unprecedented-growth-in-automotive-applications/.

[285] The Application of Aluminum Alloy in Automotive Industry. https://www.aluminiummanufacturer.com/blog/the-application-of-aluminum-alloy-in-automotive-industry/.

[286] Gray Square 9000 Series Aluminum, Grade: Good. https://www.indiamart.com/proddetail/9000-series-aluminum-25920719997.html.

[287] Xu C., Xiao W., Hanada S., Yamagata H., Ma C. (2015). The effect of scandium addition on microstructure and mechanical properties of Al–Si–Mg alloy: A multi-refinement modifier. Mater. Charact..

[288] Liu G., Blake P., Ji S. (2019). Effect of Zr on the high cycle fatigue and mechanical properties of Al–Si–Cu–Mg alloys at elevated temperatures. J. Alloys Compd..

[289] Rahimian M., Amirkhanlou S., Blake P., Ji S. (2018). Nanoscale Zr-containing precipitates; a solution for significant improvement of high-temperature strength in Al-Si-Cu-Mg alloys. Mater. Sci. Eng. A.

[290] Mohamed A.M.A., Samuel F.H., Al Kahtani S. (2013). Microstructure, tensile properties and fracture behavior of high temperature Al–Si–Mg–Cu cast alloys. Mater. Sci. Eng. A.

[291] Pushp P., Dasharath S.M., Arati C. (2022). Classification and applications of titanium and its alloys. Mater. Today Proc..

[292] Boyer R.R., Briggs R.D. (2005). The use of β titanium alloys in the aerospace industry. J. Mater. Eng. Perform..

[293] Donachie J., Matthew J. (2000). Titanium: A Technical Guide.

[294] Wang K., Kopec M., Chang S., Qu B., Liu J., Politis D.J., Wang L., Liu G. (2020). Enhanced formability and forming efficiency for two-phase titanium alloys by fast light alloys stamping technology (FAST). Mater. Des..

[295] Elias C.N., Meyers M.A., Valiev R.Z., Monteiro S.N. (2013). Ultrafine grained titanium for biomedical applications: An overview of performance. J. Mater. Res. Technol..

[296] Shahmir H., Langdon T. (2017). An evaluation of the hexagonal close-packed to face-centered cubic phase transformation in a Ti-6Al-4V alloy during high-pressure torsion. Mater. Sci. Eng. A.

[297] Kolli R.P., Devaraj A. (2018). A review of metastable beta titanium alloys. Metals.

[298] Wood J.R., Russo P.A., Welter M.F., Crist E.M. (1998). Thermomechanical processing and heat treatment of Ti–6Al–2Sn–2Zr–2Cr–2Mo–Si for structural application. Mater. Sci. Eng. A.

[299] Huang S., Zhao Q., Wu C., Lin C., Zhao Y., Jia W., Mao C. (2021). Effects of β-stabilizer elements on microstructure formation and mechanical properties of titanium alloys. J. Alloys Compd..

[300] Muraca R.F., Whittick J.S. (1972). Materials Data Handbook. Titanium 6Al-4V.

[301] Salihu S.A., Suleiman Y.I., Eyinavi A.I. (2019). Classification, Properties and Applications of titanium and its alloys used in automotive industry—A Review. Am. J. Eng. Res..

[302] Wang Z., Liu L., Zhang L., Sheng J., Wu D., Yuan M. (2019). Effect of heat treatment on the microstructure and mechanical properties of high-strength Ti–6Al–4V–5Fe alloy. Mater. Trans..

[303] Brice D.A., Samimi P., Ghamarian I., Kiu Y., Brice R.M., Reidy R.F., Cotton J.D., Kaufman M.J., Collins P.C. (2016). Oxidation behavior and microstructural decomposition of Ti-6Al-4V and Ti-6Al-4V-1B sheet. Corros. Sci..

[304] Fan Y., Tian W., Guo Y., Sun Z., Xu J. (2016). Relationships among the microstructure, mechanical properties, and fatigue behavior in thin Ti6Al4V. Adv. Mater. Sci. Eng..

[305] Loier C., Thauvin Hazotte G.A., Simon A. (1985). Influence of deformation on the β→α+β transformation kinetics of Ti-6 wt.%Al-4 wt.%V alloy. J. Less Common Met..

[306] Leyens C., Peter M. (2003). Titanium and Titanium Alloys: Fundamentals and Applications.

[307] Kang L.M., Yang C. (2019). A review on high-strength titanium alloys: Microstructure, strengthening, and properties. Adv. Eng. Mater..

[308] Yamashita Y., Takayama I., Fujii H., Yamazaki T. (2002). Applications and features of titanium for automotive industry. Nippon Steel Tech. Rep..

[309] Bieler T.R., Trevino R.M., Zeng L. (2005). Alloys: Titanium. Encyclopedia of Condensed Matter Physics.

[310] Sbayti M., Ghiotti A., Bahloul R., Belhadjsalah H., Bruschi S. (2016). Finite element analysis of hot single point incremental forming of hip prostheses. MATEC Web Conf..

[311] Sornsuwit N., Sittisakuljaroen S., Sangsai N., Suwankan P. Effect of heat treatment on single point incremental forming for titanium Grade 2 sheet. Proceedings of the 2018 Third International Conference on Engineering Science and Innovative Technology (ESIT).

[312] Mechanical Properties of Titanium Alloy. https://www.kobelco.co.jp/english/titan/files/details.pdf.

[313] You S.H., Lee J.H., Oh S.H. (2019). A Study on Cutting Characteristics in Turning Operations of Titanium Alloy used in Automobile. Int. J. Precis. Eng. Manuf..

[314] Gialanella S., Malandruccolo A., Gialanella S., Malandruccolo A. (2020). Titanium and Titanium Alloys. Aerospace Alloys.

[315] Drossou-Agakidou V., Kanakoudi-Tsakalidou F., Sarafidis K., Taparkou A., Tzimouli V., Tsandali H., Kremenopoulos G. (1998). Administration of recombinant human granulocytecolony stimulating factor to septic neonates induces neutrophilia and enhances the neutrophil respiratory burst and β2 integrin expression results of a randomized controlled trial. Eur. J. Pediatr..

[316] Leyens C., Peters M. (2003). Titanium and Titanium Alloys.

[317] Takahashi K., Mori K., Takebe H. (2020). Application of Titanium and its Alloys for Automobile Parts. MATEC Web Conf..

[318] Wollmann M., Kiese J., Wagner L. Properties and applications of titanium alloys in transport. Proceedings of the 12th World Conference on Titanium, China National Convention Center (CNCC).

[319] Nyamekye P., Rahimpour Golroudbary S., Piili H., Luukka P., Kraslawski A. (2023). Impact of additive manufacturing on titanium supply chain: Case of titanium alloys in automotive and aerospace industries. Adv. Ind. Manuf. Eng..

[320] Froes F.H. (1994). Advanced metals for aerospace and automotive use. Mater. Sci. Eng. A.

[321] Fujii H., Takahashi K., Yamashita Y. (2003). Application of titanium and its alloys for automobile parts. Nippon Steel Tech. Rep..

[322] Saito T. (2004). The automotive application of discontinuously reinforced TiB-Ti composites. JOM.

[323] Kosaka Y., Fox S.P., Faller K. (2004). Newly developed titanium alloy sheets for the exhaust systems of motorcycles and automobiles. JOM.

[324] Oldenberger E.L., Oldenburg M., Thilderkvist P., Stoehr T., Lechler J., Merklein M. (2011). Tool development based on modelling and simulation of hot sheet metal forming of Ti–6Al–4V titanium alloy. J. Mater. Process. Technol..

[325] Göttmann A., Diettrich J., Bergweiler G., Bambach M., Hirt G., Loosen P., Poprawe R. (2011). Laser-assisted asymmetric incremental sheet forming of titanium sheet metal Parts. Prod. Eng. Res. Dev..

[326] Gagliardi F., Ambrogio G., Filice L. (2017). Incremental forming with local induction heating on materials with magnetic and non-magnetic properties. Procedia Eng..

[327] Oleksik V., Trzepieciński T., Szpunar M., Chodoła Ł., Ficek D., Szczęsny I. (2021). Single-point incremental forming of titanium and titanium alloy sheets. Materials.

[328] Trzepieciński T., Oleksik V., Pepelnjak T., Najm S.M., Paniti I., Maji K. (2021). Emerging trends in single point incremental sheet forming of lightweight metals. Metals.

[329] Application of Titanium Alloy in Automobile. https://titanium.net/application-of-titanium-alloy-in-automobile/.

[330] Titanium and Its Alloy Used for Automotive Applications. https://energy-ti.com/titanium-and-its-alloy-used-for-automotive-applications/.

[331] Furuta T., Froes F., Qian M., Niinomi M. (2019). Automobile applications of titanium. Titanium for Consumer Applications Real World Use of Titanium.

[332] Applications of Titanium Alloy in the Automobile Industry. https://www.refractorymetal.org/applications-of-titanium-alloy-in-automobile-industry/.

[333] Application of Titanium in the Automotive Industry. https://www.yunchtitanium.com/news/application-of-titanium-in-the-automotive-indu-34966228.html.

[334] Isaka M., Takebe H., Kawakami A., Takahashi K. (2022). Applications of Titanium for the Automotive Sector. Nippon Steel Tech. Rep..

[335] Bloodhound Car Begins to Take Shape. https://www.bbc.com/news/science-environment-31694204.

[336] Wagner L., Schauerte O., Ninomi M., Akiyama S., Ikeda M., Hagiwara M., Maruyama K. (2007). Status of Titanium and Titanium Alloys in Automotive Applications. Ti-2007 Science and Technology.

[337] Veiga C., Davim J.P., Loureiro A.J.R. (2012). Properties and applications of titanium alloys: A brief review. Rev. Adv. Mater. Sci..

[338] Czerwinski F. (2021). Current Trends in Automotive Lightweighting Strategies and Materials. Materials.

[339] Dziadoń A., Mola R. (2023). Magnez. Kierunki kształtowania własności mechanicznych. Obróbka Plast. Met..

[340] Dziadoń A. (2012). Magnez i Jego Stopy.

[341] Roberts C.S. (1960). Magnesium and Its Alloy.

[342] Ghali E., Revie R.W. (2011). Magnesium and magnesium alloys. Uhlig’s Corrosion Handbook.

[343] Kuczmaszewski J., Zagórski I., Kuczmaszewski J., Zaleski K. (2015). Magnez i jego stopy. Obróbka Skrawaniem Stopów Magnezu.

[344] Hadasik E., Kuc D., Szuła A. (2010). Kształtowanie plastyczne stopu magnezu AZ31. Rudy Met. Nieżelazne.

[345] Powell B.R., Luo A.A., Krajewski P.E., Rowe J. (2012). Magnesium alloys for lightweight powertrains and automotive bodies. Advanced Materials in Automotive Engineering.

[346] Neite G., Kubota K., Higashi K., Hehmann F., Cahn R.W., Haasen P., Kramer E.J. (2005). Magnesium-based alloys. Materials Science and Technology.

[347] Gray J.E., Luan B. (2002). Protective coatings on magnesium and its alloys—A critical review. J. Alloys Compd..

[348] Zhao D.B. (2014). The FEA Comparison of the Front Sub Frame in a Car between JDM2 Magnesium and steel. Appl. Mech. Mater..

[349] Liu B., Yang J., Zhanmg X., Yang Q., Zhang J., Li X. (2023). Development and application of magnesium alloy parts for automotive OEMs: A review. J. Magn. Alloys.

[350] Powell B.R., Krajewski P.E., Luo A.A. (2021). Magnesium Alloys for Lightweight Powertrains and Automotive Structures. Materials, Design and Manufacturing for Lightweight Vehicles.

[351] Luo A.A. (2013). Applications: Aerospace, Automotive and Other Structural Applications of Magnesium. Fundamentals of Magnesium Alloy Metallurgy.

[352] Joost W.J., Krajewski P.E. (2007). Towards magnesium alloys for high-volume automotive applications. Scr. Mater..

[353] Luo A.A., Quinn J.F., Wang Y.M., Lee T.M., Verma R., Wagner D.A., Forsmark J.H., Su X., Zindel J., Li M. (2012). The USAMP magnesium front end research and development project: Focusing on a demonstration structure. Light Met. Age.

[354] Luo A.A., Shi R., Miao J., Avey T. (2021). Review: Magnesium sheet alloy development for room temperature forming. JOM.

[355] Tan J., Ramakrishna S. (2021). Applications of Magnesium and Its Alloys: A Review. Appl. Sci..

[356] Golroudbary S.R., Makarava I., Repo E., Kraslawski A., Luukka P. (2022). Magnesium life cycle in automotive industry. Procedia CIRP.

[357] Xue Y., Horstemeyer M.F., McDowell D.L., El Kadiri H., Fan J. (2007). Microstructure-based multistage fatigue modeling of a cast AE44 magnesium alloy. Int. J. Fatigue.

[358] Khademian N., Peimaei Y. Magnesium alloys and applications in automotive industry. Proceedings of the 5th International Conference on Science and Development of Nanotechnology.

[359] Hector B., Heiss W. (1990). Magnesium Die-Castings as Structural Members in the Integral Seat of the New Mercedes-Benz Roadster.

[360] Kumar D.S., Sasanka C.T., Ravindra K., Suman K.N.S. (2015). Magnesium and Its Alloys in Automotive Applications—A Review. Am. J. Mater. Sci. Technol..

[361] Aune T.K., Westengen H., Ruden T. (1993). Mechanical Properties of Energy Absorbing Magnesium Alloys.

[362] Alves H., Koster U., Aghion E., Eliezer D. (2001). Environmental Behavior of Magnesium and Magnesium Alloys. Mater. Technol..

[363] Magnesium Semisolid Forming Equipment. SSD-Magnesium. http://www.ssd-magnesium.com/product/I8rC1o.html.

[364] Blanchard P.J., Bretz G.T., Subramanian S. (2005). The Application of Magnesium Die Casting to Vehicle Closures.

[365] Hubbert T., Chen X., Li N., Pineo S. (2004). 2005 Ford GT Magnesium I/P Structure.

[366] Fan S., Wang X., Wang G.G., Weiller J.P., Tański T.A., Cesarz-Andraczke K., Jonda E. (2023). Applications of High-Pressure Die-Casting (HPDC) Magnesium Alloys in Industry. Magnessium Alloys—Processing, Potential and Applications.

[367] Gerken R.T., Ghaffari B., Sachdev A.K., Mehta M., Carter J.T. (2023). Low-cost magnesium alloy sheet component development and demonstration project. SAE Int. J. Adv. Curr. Prac. Mobil..

[368] Sadayappan K., Vassos M. (2010). Evaluation of a Thixomolded Magnesium Alloy Component for Automotive Application.

[369] Logan S., Kizyma A., Patterson C., Rama S. (2006). Lightweight Magnesium Intensive Body Structure. SAE Trans..

[370] Magnesium Alloy Parts Application Case Studies (Client Provides Up to 5). https://www.yiruimetalmg.com/magnesium-alloy-parts-application-case-studies-client-provides-up-to-5.html.

[371] Westengen H., Bakke P. (2003). Magnesium Die Casting Alloys for Use in Applications Exposed to Elevated Temperatures: Can They Compete with Aluminium?. Mater. Sci. Forum.

[372] Pekguleryuz M.O., Baril E., Mathaudhu S.N., Luo A.A., Neelameggham N.R., Nyberg E.A., Sillekens W.H. (2016). Development of Creep Resistant Mg-Al-Sr alloys. Essential Readings in Magnesium Technology.

[373] Verma R., Carter J.T. (2006). Quick Plastic Forming of a Decklid Inner Panel with Commercial AZ31 Magnesium Sheet.

[374] Carter J.T., Krajewski P.E., Verma R. (2008). The Hot Blow Forming of AZ31 Mg Sheet: Formability Assessment and Application Development. JOM.

[375] Luo A.A., Forsmark J., Sun X., Shook S. (2010). Mechanical and Thermophysical Properties of Magnesium Alloy Extrusions.

[376] Wickberg A., Ericsson R. (1985). Magnesium in the Volvo LCP 2000.

[377] Jekl J., Auld J., Sweet C., Carter J., Resch S., Klarner A., Brevick J., Luo A. (2015). Development of a Thin-Wall Magnesium Side Door Inner Panel for Automobiles.

[378] Hawke D., Gaw K. (1992). Effects of Chemical Surface Treatments on the Performance of an Automotive Paint System on Die Cast Magnesium.

[379] Grebetz J.C. (1993). A Comparison of the Impact Characteristics of Several Magnesium Die Casting Alloys.

[380] Murray R.W., Hillis J.E. (1990). Magnesium Finishing: Chemical Treatment and Coating Practices.

[381] Annamalai S., Periyakgoundar S., Paramasivam K., Selvaraj A.B. (2020). Investigation of Bending, Sound Absorption, and Damping Properties of AZ91D-Swivel Plate. Adv. Mater. Sci. Eng..

[382] Aune T.K., Westengen H., Ruden T. (1994). The effects of varying aluminum and rare-earth content on the mechanical properties of die cast magnesium alloys. SAE Trans..

[383] Durairaj S.R.N., Ganesan T., Rao P.C. (2017). Vibration Analysis on Magnesium Alloy Housing and Analysis of Resonant Frequency on the Housing between Magnesium and Aluminium Alloy.

[384] Froes F.H., Eliezer D., Aghion E. (1998). The science, technology, and applications of magnesium. JOM.

[385] Luo A.A. (2013). Magnesium casting technology for structural applications. J. Magn. Alloys.

[386] Riopelle L. Magnesium Applications. Proceedings of the International Magnesium Association (IMA) Annual Magnesium in Automotive Seminar.

[387] An Assessment of Mass Reduction Opportunities for a 2017–2020 Model Year Vehicle Program. http://theicct.org/sites/default/files/publications/Mass_reduction_final_2010.pdf.

[388] Weiler J.P. (2019). A review of magnesium die-castings for closure applications. J. Magnes. Alloys.

[389] US, Chinese Automakers Increasingly Use Magnesium as Vehicle Body Material. https://aaa.fourin.com/reports/8fb96120-2860-11ea-8162-d9787bdc6011/us-chinese-automakers-increasingly-use-magnesium-as-vehicle-body-material.

[390] Wang J., Pang X., Jahed H. (2019). Surface protection of Mg alloys in automotive applications: A review. AIMS Mater. Sci..

[391] Ahmad H., Markina A.A., Porotnikov M.V., Ahmad F. (2020). A review of carbon fiber materials in automotive industry. IOP Conf. Ser. Mater. Sci. Eng..

[392] Wazeer A., Das A., Abeykoon C., Sinha A., Karmakar A. (2023). Composites for electric vehicles and automotive sector: A review. Green Energy Intell. Transp..

[393] Wan Y., Takahashi J. (2021). Development of Carbon Fiber-Reinforced Thermoplastics for Mass-Produced Automotive Applications in Japan. J. Compos. Sci..

[394] Battaglia M., Sellitto A., Giamundo A., Visone M., Riccio A. (2023). Shape Memory Alloys Applied to Automotive Adaptive Aerodynamics. Materials.

[395] Riccio A., Sellitto A., Ameduri S., Concilio A., Arena M. (2021). Shape memory alloys (SMA) for automotive applications and challenges. Shape Memory Alloy Engineering.

[396] Jani J.M., Leary M., Subic A. (2014). Shape Memory Alloys in Automotive Applications. Appl. Mech. Mater..

[397] Tuazon B.J., Custodio N.A.V., Basuel R.B., Delos Reyes L.A., Dizon J.R.C. (2022). 3D Printing Technology and Materials for Automotive Application: A Mini-Review. Key Eng. Mater..

[398] Shahrubudin N., Lee T.C., Ramlan R. (2019). An Overview on 3D Printing Technology: Technological, Materials, and Applications. Procedia Manuf..

[399] Elakkad A.S. (2019). 3D Technology in the Automotive Industry. Int. J. Eng. Res..

[400] Gechev T. (2021). A short review of 3D printing methods used in the automotive industry. Bulg. J. Eng. Des..

